# Disruption of CFAP418 interaction with lipids causes widespread abnormal membrane-associated cellular processes in retinal degenerations

**DOI:** 10.1172/jci.insight.162621

**Published:** 2024-01-09

**Authors:** Anna M. Clark, Dongmei Yu, Grace Neiswanger, Daniel Zhu, Junhuang Zou, J. Alan Maschek, Thomas Burgoyne, Jun Yang

**Affiliations:** 1 Department of Ophthalmology and Visual Sciences, Moran Eye Center, and; 2 Department of Nutrition and Integrative Physiology, University of Utah, Salt Lake City, Utah, USA.; 3 UCL Institute of Ophthalmology, University College of London, London, United Kingdom.; 4 Department of Otolaryngology, and; 5 Department of Neurobiology, University of Utah, Salt Lake City, Utah, USA.

**Keywords:** Cell Biology, Ophthalmology, Protein traffic, Proteomics, Retinopathy

## Abstract

Syndromic ciliopathies and retinal degenerations are large heterogeneous groups of genetic diseases. Pathogenic variants in the *CFAP418* gene may cause both disorders, and its protein sequence is evolutionarily conserved. However, the disease mechanism underlying *CFAP418* mutations has not been explored. Here, we apply quantitative lipidomic, proteomic, and phosphoproteomic profiling and affinity purification coupled with mass spectrometry to address the molecular function of *CFAP418* in the retina. We show that CFAP418 protein binds to the lipid metabolism precursor phosphatidic acid (PA) and mitochondrion-specific lipid cardiolipin but does not form a tight and static complex with proteins. Loss of *Cfap418* in mice disturbs membrane lipid homeostasis and membrane-protein associations, which subsequently causes mitochondrial defects and membrane-remodeling abnormalities across multiple vesicular trafficking pathways in photoreceptors, especially the endosomal sorting complexes required for transport (ESCRT) pathway. Ablation of *Cfap418* also increases the activity of PA-binding protein kinase Cα in the retina. Overall, our results indicate that membrane lipid imbalance is a pathological mechanism underlying syndromic ciliopathies and retinal degenerations which is associated with other known causative genes of these diseases.

## Abstract 2

Membrane lipid imbalance and abnormal membrane-associated cellular processes underlie CFAP418-associated retinal degeneration.

## Graphical abstract

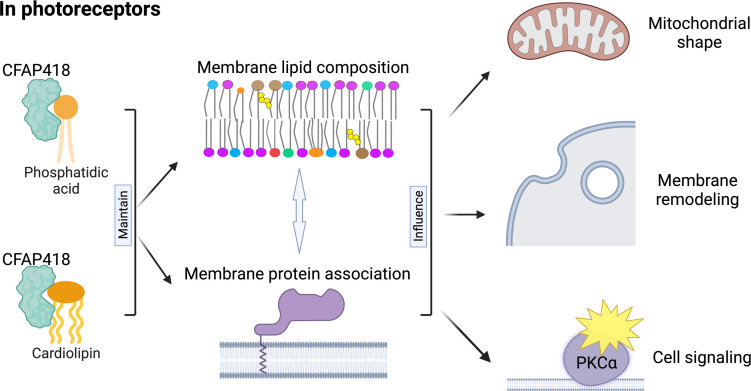

## Introduction

Eukaryotic cells have subcellular organelles that conduct specialized activities essential for cell growth, differentiation, homeostasis, survival, and function. Most organelles are separated from the cytoplasm by lipid bilayer membranes and exchange materials through intracellular trafficking to coordinate their activities and enable cells to act as an entity. During membrane intracellular trafficking, vesicles are budded at specific sites of donor organelles, travel through the cytoplasm, and dock at and fuse with the membranes of target organelles. These processes are mediated by various membrane proteins and lipids (1, 2). The membrane lipids consist of numerous glycerophospholipids, sphingolipids, and sterols. The composition and distribution of these lipids determine the membrane’s biophysical and biochemical properties and membrane associations with proteins. However, because of the substantial complexity and diversity of membrane lipids and the limitations of available tools to study lipid molecules and membrane properties, the mechanisms by which membrane lipids maintain cell organelle integrity and participate in membrane remodeling during vesicular transport remain understudied (3, 4).

**Figure 1:**
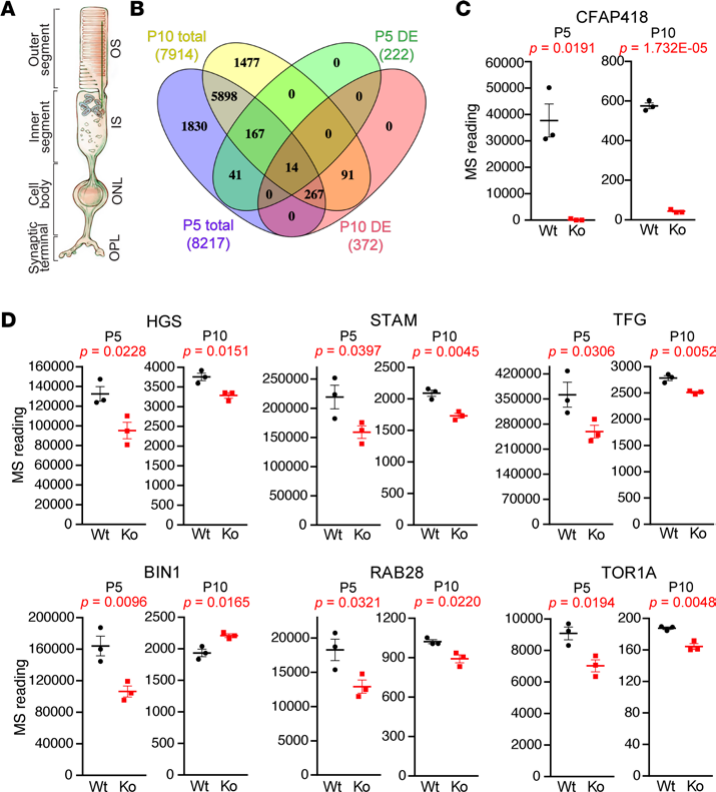
Membrane remodeling–associated proteins are differentially expressed at the onset of *Cfap418^–/–^* retinal phenotypes. (**A**) Schematic of photoreceptor subcellular compartments, which occupy the outer segment (OS), inner segment (IS), outer nuclear layer (ONL), and outer plexiform layer (OPL) in the retina. This schematic was adapted from Mathur and Yang (92). (**B**) Venn diagram showing the total proteins and differentially expressed proteins in P5 and P10 retinas detected by quantitative proteomics. (**C**) Scarcity of CFAP418 protein in *Cfap418^–/–^* (Ko) retinas validates our quantitative proteomic study. (**D**) Six membrane remodeling–associated proteins are differentially expressed in both P5 and P10 *Cfap418^–/–^* retinas, detected by label-free and TMT-labeling quantitative MS, respectively. Dot plots are represented as data from individual mice and mean ± SEM (2-tailed Student’s *t* test).

Photoreceptors are highly specialized neurons and are an excellent cell model for studying membrane lipid homeostasis and intracellular vesicular trafficking (5, 6). From the apex to the base, photoreceptors are composed of a modified primary cilium known as the outer segment (OS), which is connected to the inner segment (IS), progressing through to the cell body within the outer nuclear layer (ONL), and finally the synaptic terminus within the outer plexiform layer (OPL) of the retina (Figure 1A). The IS is the cell compartment for most protein and membrane lipid biosynthesis. The synthesized proteins and membrane lipids are transported to other photoreceptor regions to generate different cell compartments during retinal development and to maintain cell homeostasis within mature retinas. The OS contains many tightly stacked membrane disks, where phototransduction occurs. These disks rapidly renew every 10 days in mammals as a result of phagocytosis of the OS by the retinal pigment epithelium (RPE) to overcome light-induced oxidative damage. Mutations in genes functioning in photoreceptor vesicular transport lead to photoreceptor cell death in a large heterogeneous group of inherited retinal degenerations (IRDs). The molecular basis of the intracellular vesicular transport in photoreceptors, however, has not been well elucidated.

Cilia- and flagella-associated protein 418 (*CFAP418*) is a causative gene for retinitis pigmentosa (7–9), cone-rod dystrophy (9–11), Bardet-Biedl syndrome (BBS) (12, 13), and combined retinal dystrophy and macular atrophy (14). While all these IRDs affect photoreceptors, BBS is a syndromic ciliopathy and affects ciliated cells in multiple tissues. Patients with BBS caused by *CFAP418* mutations exhibit overweight, postaxial polydactyly, horseshoe kidney, and mild learning difficulty, in addition to vision problems (12, 13). CFAP418 protein is 207 amino acids long in humans and has no known functional domains. This protein sequence is highly conserved among species, including those in the lower-eukaryote genus *Chlamydomonas* (Supplemental Figure 1A; supplemental material available online with this article; https://doi.org/10.1172/jci.insight.162621DS1) and thus may play a fundamental role in eukaryotic cells. In zebrafish, *cfap418* knockdown leads to embryonic Kupffer’s vesicle defects, retrograde melanosome transport delay, and visual impairment (12). We previously generated *Cfap418*-knockout (*Cfap418^–/–^*) mouse models with different mutations (15). These *Cfap418^–/–^* mice display reduced electroretinogram responses, followed by photoreceptor cell death. *Cfap418^–/–^* photoreceptors exhibit extensive OS disk misalignment and OS membrane protein reduction, although CFAP418 is localized to the IS. The photoreceptor phenotypes emerge at postnatal day 5 (P5) during ciliogenesis and become evident after P10 when OS grows robustly during development. Using *Cfap418^–/–^* tissues as a control, we found that CFAP418 protein is expressed in many mouse tissues, including the brain, heart, kidney, trachea, lung, testis, and spleen. The exact molecular function of CFAP418 in photoreceptor ciliogenesis and OS growth remains unexplored.

Here, we applied unbiased omics approaches, including affinity purification coupled with mass spectrometry (AP-MS) and quantitative MS to investigate CFAP418-interacting proteins and the effects of *Cfap418* knockout on protein expression, phosphorylation, and membrane lipid composition in the retina. We unexpectedly discovered that CFAP418 exhibits preferential binding to the membrane lipid precursor phosphatidic acid (PA) and mitochondrion-specific lipid cardiolipin (CL), rather than to protein partners. Through these lipid interactions, CFAP418 maintains membrane lipid homeostasis, which is crucial for multiple membrane-associated cellular processes. This function of CFAP418 likely occurs in both ciliated and nonciliated cells. Our study also reveals that disturbance of membrane lipid homeostasis represents a pathological mechanism underlying ciliopathies and IRDs.

## Results

### Membrane remodeling–associated proteins are altered in Cfap418^–/–^ photoreceptor IS during ciliogenesis.

To identify the early-onset primary defects in *Cfap418^–/–^* photoreceptors with consideration that photoreceptors account for approximately 80% of all mouse retinal cells (16), we surveyed the protein expression profiles in wild-type (WT) and *Cfap418^–/–^* littermate retinas in an unbiased manner using proteome-wide quantitative MS at P5 and P10. At P5, label-free quantitative MS detected 193 downregulated and 29 upregulated proteins (*P* < 0.05; Supplemental Table 2) from a total of 8,217 detected proteins in *Cfap418^–/–^* retinas (Figure 1B and Supplemental Table 1). At P10, tandem mass tag (TMT) quantitative MS identified 233 downregulated and 139 upregulated proteins (*P* < 0.05; Supplemental Table 2) from a total of 7,914 detected proteins in *Cfap418^–/–^* retinas (Figure 1B and Supplemental Table 1). As expected, *Cfap418* knockout led to barely detectable CFAP418 protein expression in both P5 and P10 retinas (Figure 1C).

Thirteen differentially expressed (DE) proteins were identified in both the P5 and P10 quantitative MS experiments (Supplemental Table 3). Six of them were associated with membrane remodeling in vesicular trafficking pathways. They were hepatocyte growth factor-regulated tyrosine kinase substrate (HGS), signal transducing adaptor molecule (STAM), Trk-fused gene (TFG), bridging integrator-1 (BIN1), torsin-1A (TOR1A), and RAB28 (Figure 1D). These proteins are involved in the endosomal sorting complex required for transport (ESCRT) pathway (RAB28, HGS, and STAM), BBSome-mediated ciliary transport (RAB28), ER to Golgi (TFG) and ER to nuclear envelop (TOR1A) trafficking, endosomal (BIN1) processes, and ciliary extracellular vesicle and photoreceptor OS shedding (RAB28) (17–26) (Supplemental Table 3). These proteins were reduced in *Cfap418^–/–^* retinas at both P5 and P10, except that BIN1 was increased at P10 (Figure 1D). Although the magnitude of these proteins’ fold changes (FCs, ratio of *Cfap418^–/–^* to WT MS value) was moderate, in the range of 13%–35%, the MS values of these DE proteins, except TOR1A at P10, were all above or around the median MS values in each animal and thus were reliable. Additionally, our quantitative MS experiments were conducted on independent sets of retinas at P5 and P10 using 2 completely different protocols (label-free and TMT labeling) by 2 different proteomics core facilities. We considered the DE proteins identified from both the P5 and P10 MS experiments to be cross-confirmed. Because the DE proteins were identified in *Cfap418^–/–^* retinas as early as P5 during the photoreceptor ciliogenesis, when the photoreceptor OS is not formed (27), the membrane-remodeling defects should occur in the photoreceptor IS, where CFAP418 is located (15).

**Figure 2:**
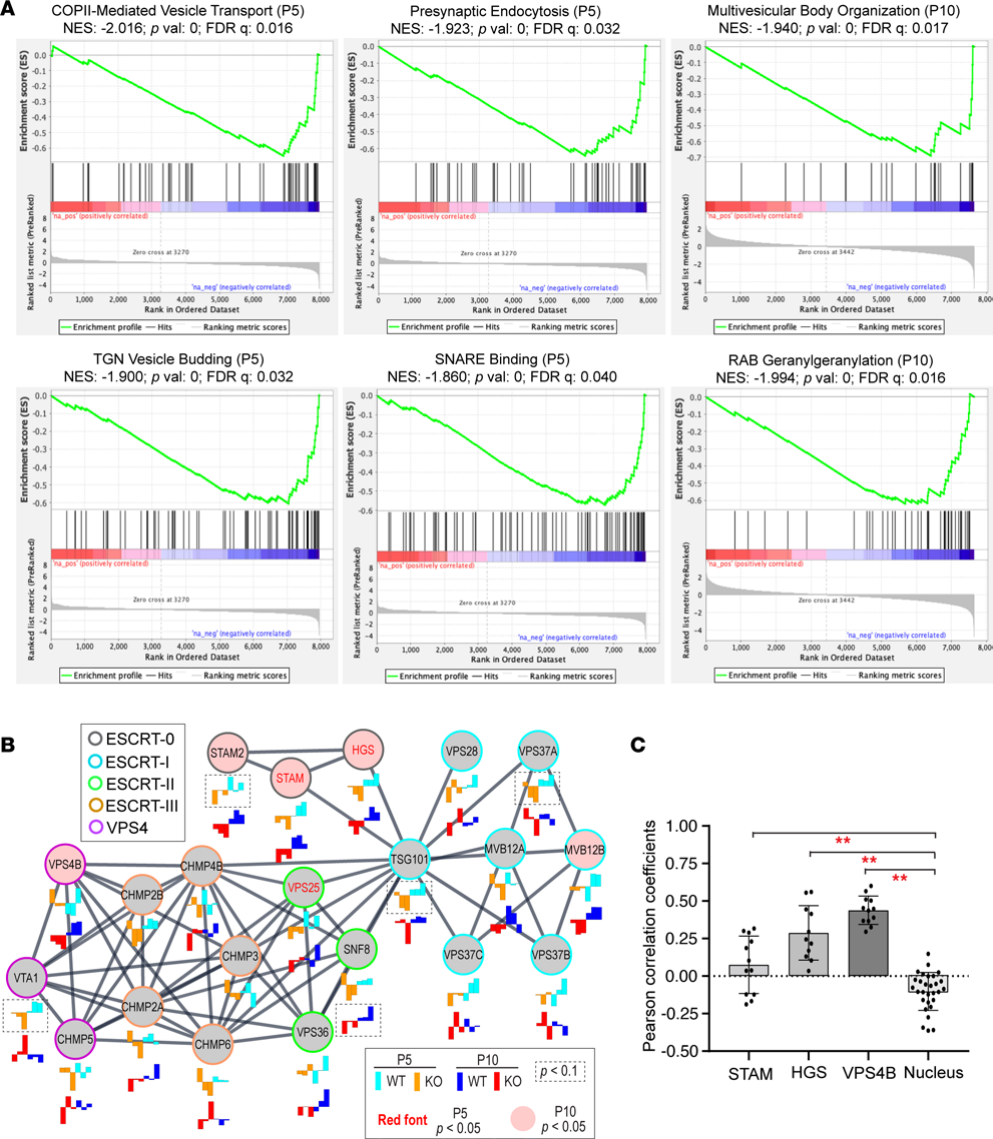
CFAP418 functions in membrane remodeling–associated pathways. (**A**) GSEA reveals that vesicular trafficking processes are negatively affected in P5 and P10 *Cfap418^–/–^* retinas. (**B**) Expression of ESCRT complex components in *Cfap418^+/+^* and *Cfap418^–/–^* littermate retinas at P5 and P10 (2-tailed Student’s *t* test). The expression levels of 3 individual mice per genotype are shown as bar charts below each node. Lines between nodes represent associations between nodes annotated by STRING/Cytoscape 3.8.1 (93, 94). (**C**) Pearson’s correlation coefficients of FLAG-CFAP418 with endogenous STAM, HGS, and VPS4B in COS-7 cells. The bar plot represents data from individual cells and mean ± SEM. **P < 0.01 (1-way ANOVA with Tukey’s multiple-comparison test).

We then conducted gene set enrichment analysis (GSEA) on P5 and P10 retinal proteomes to investigate which cellular pathways and organelles were abnormal in *Cfap418^–/–^* retinas. Consistent with the phenotypes we previously observed in mature *Cfap418^–/–^* retinas (15), GSEA identified downregulated gene sets of photoreceptor IS proteins (*P* = 0.002; false discovery rate [FDR] *q* = 0.045) and phototransduction proteins (*P* = 0, FDR *q* = 0.067) at P5 and a downregulated gene set of ciliary membrane proteins that includes photoreceptor OS membrane proteins at P10 (*P* = 0, FDR *q* = 0.013) (Supplemental Figure 2 and Supplemental Table 4). GSEA also identified many downregulated gene sets involved in vesicle budding, transport, targeting, and tethering, including the COPII-mediated vesicle transport and SNARE binding gene sets at P5 and multivesicular body (MVB) organization and RAB geranylgeranylation gene sets at P10 (Figure 2A and Supplemental Table 4). These findings were supported by our previous observation of MVB accumulation in *Cfap418^–/–^* photoreceptor IS (15). Therefore, multiple membrane remodeling–related proteins and cellular pathways were altered in *Cfap418^–/–^* photoreceptors. Furthermore, we observed that overexpression of FLAG- and GFP-CFAP418, but not GFP, in COS-7 cells resulted in large vacuole accumulation in the cytoplasm (arrows; Supplemental Figure 3, Supplemental Figure 4B, and Figure 8B). Taken together, our data from CFAP418 ablation in photoreceptors and CFAP418 overexpression in mammalian cultured cells indicate that CFAP418 plays a role in membrane remodeling.

To further investigate the role of CFAP418 in membrane remodeling, we focused on ESCRT proteins in the P5 and P10 retinal proteomes. Besides HGS and STAM, other ESCRT-0 (STAM2), ESCRT-I (MVB12B, VPS37A, and TSG101), ESCRT-II (VPS25 and SNF8), and VPS4 (VPS4B and VTA1) proteins were also reduced (*P* < 0.05) or had a trend of reduction (*P* < 0.1) in P5 or P10 *Cfap418^–/–^* retinas (Figure 2B). Additionally, we found that CFAP418 was partially colocalized with endogenous VPS4B, HGS, and STAM in FLAG-CFAP418–transfected COS-7 cells (Figure 2C and Supplemental Figure 4A).

**Figure 3:**
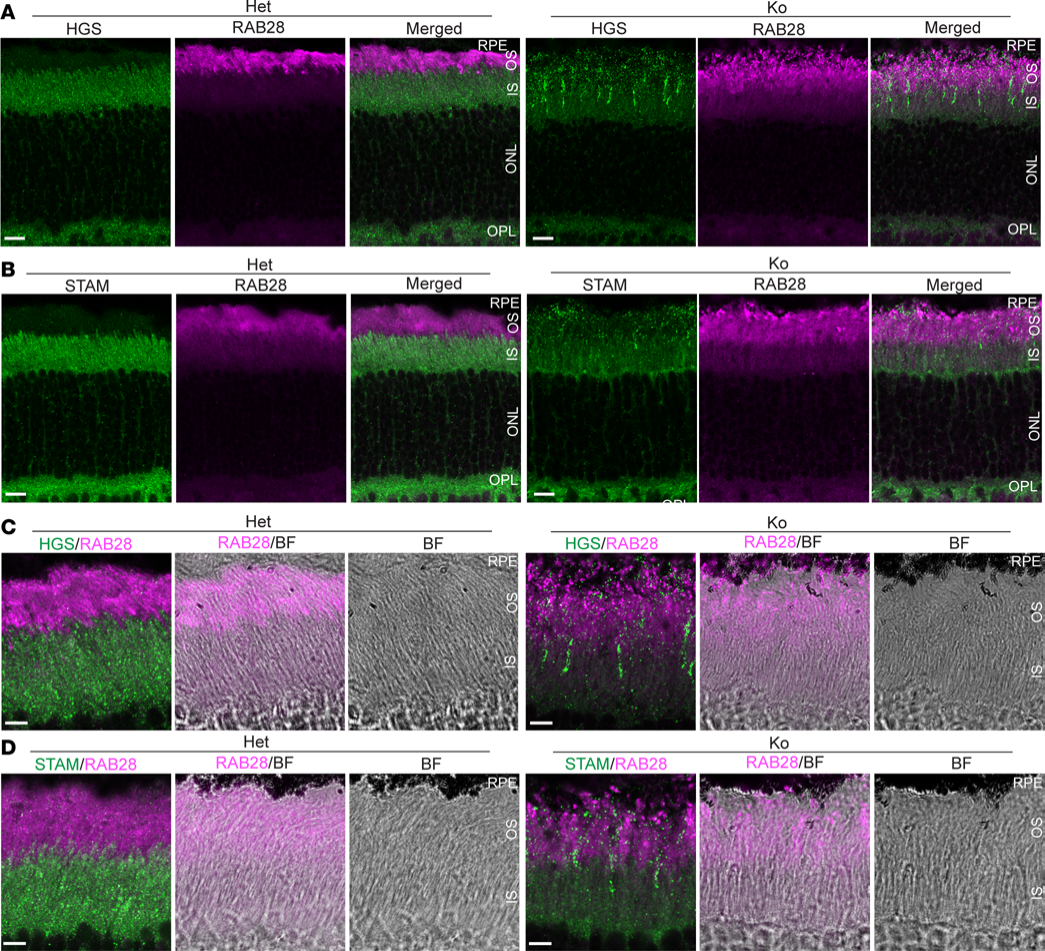
Abnormal HGS, STAM, and RAB28 distributions in *Cfap418^–/–^* photoreceptors. (**A** and **B**) Compared with heterozygous (Het, *Cfap418^+/–^*) littermate photoreceptors, HGS (**A**) and STAM (**B**) are mislocalized from the IS to the OS, and RAB28 signal shows an abnormal punctate pattern at the OS and RPE junction in P21 *Cfap418^–/–^* (Ko) photoreceptors. RAB28 signal shows an abnormal punctate pattern at the OS and RPE junction in P21 *Cfap418^–/–^* retinas. (**C** and **D**) Enlarged view of HGS (**C**), STAM (**D**), and RAB28 immunostaining signals in the OS and IS regions of P21 *Cfap418^+/–^* and *Cfap418^–/–^* photoreceptors. BF, bright-field. Scale bars: 10 mm (**A** and **B**) and 5 mm (**C** and **D**).

We then examined the distributions of ESCRT, endosomal, RAB28, and TFG proteins in *Cfap418^–/–^* photoreceptors by immunostaining. All of these proteins were localized normally at P5 (not shown). At P10, STAM and HGS appeared as large puncta primarily in *Cfap418^–/–^* OS, while they were present as fine puncta in *Cfap418^+/–^* IS and OS (Supplemental Figure 5, A and B). At P21, STAM and HGS displayed reduced IS signals and prominent punctate OS signals in *Cfap418^–/–^* photoreceptors, while these 2 proteins were diffused in the IS of *Cfap418^+/-^* photoreceptors (Figure 3). The distributions of STAM and HGS were, however, normal in *Cfap418^–/–^* photoreceptor OPL at P10 and P21 (Figure 3 and Supplemental Figure 5, A and B). At these 2 time points, RAB28 immunoreactivity was diffusely distributed mostly in *Cfap418^+/–^* OS (Figure 3 and Supplemental Figure 5). However, it became fragmented at the OS and RPE junction in *Cfap418^–/–^* photoreceptors at P21 (Figure 3). The immunoreactive signal patterns of TFG, VPS4B, EEA1, RAB5, RAB7, and RAB11 appeared relatively normal in *Cfap418^–/–^* photoreceptors at P10 and/or P21 (Supplemental Figure 6). In summary, our data indicate that *Cfap418* deficiency alters the expression of proteins related to cell membrane remodeling across multiple vesicular membrane trafficking pathways in photoreceptor IS during ciliogenesis, which eventually causes mislocalization of ESCRT-0 proteins to the OS and abnormal distribution of RAB28 protein at the OS apex in photoreceptors.

### Cfap418^–/–^ photoreceptors exhibit defects in mitochondrial protein expression and morphology.

**Figure 4:**
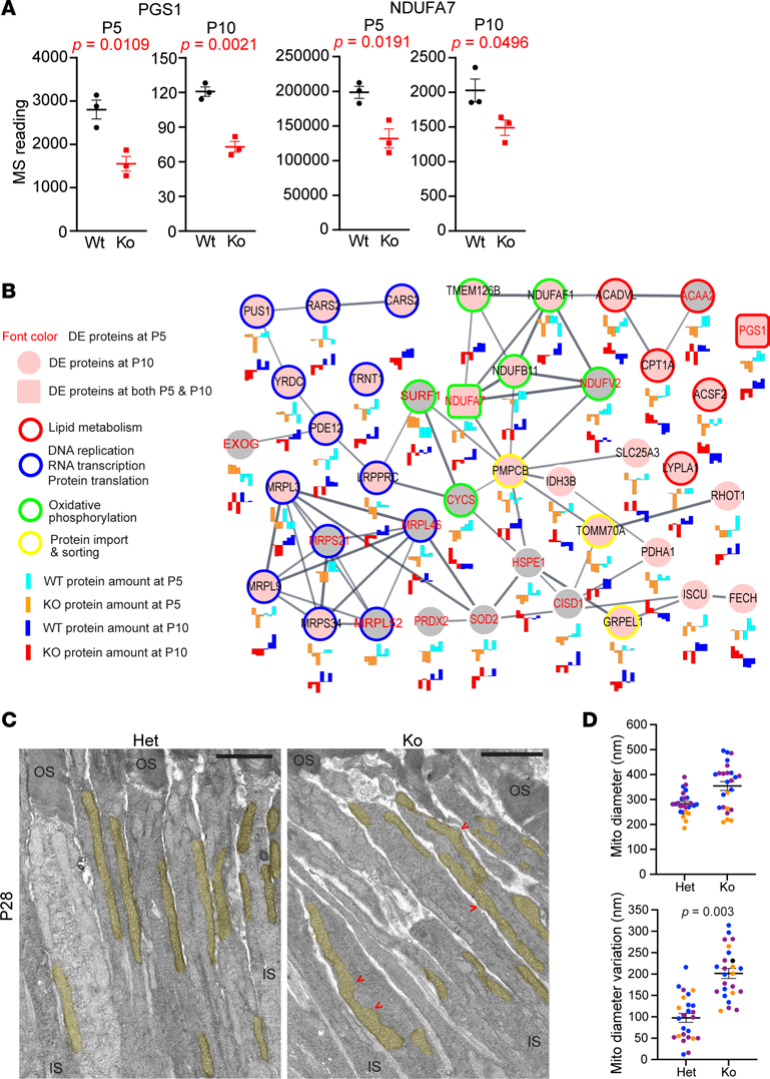
Mitochondrial protein expression and morphology are defective in *Cfap418^–/–^* photoreceptors. (**A**) Quantitative proteomic study reveals reductions in mitochondrial PGS1 and NDUFA7 proteins in both P5 and P10 *Cfap418^–/–^* retinas. Data are presented as individual mice, mean, and SEM. (**B**) The abundances of mitochondrial proteins in central dogma, oxidative phosphorylation, lipid metabolism, and protein import/sorting are altered in *Cfap418^–/–^* retinas at P5 or P10. (**C**) A longitudinal view of *Cfap418^–/–^* photoreceptors shows uneven diameters along the length of mitochondria at P28, compared with the smooth, straight, long bar-shaped mitochondria in *Cfap418^+/–^* littermate photoreceptors. Mitochondria are highlighted in yellow. Red arrows point to the abnormal constrictions and protruding bumps of the mitochondria. Scale bars: 1.5 mm. (**D**) Quantification of photoreceptor mitochondrial diameter and diameter variation at P28–P30. Data are presented as individual mitochondria (dots), mice (color), mean, and SEM. Two-tailed Student’s *t* test was conducted on averages from different mice between genotypes (*n* = 3 mice for each genotype).

Among the 13 DE proteins identified in both P5 and P10 *Cfap418^–/–^* retinas from our quantitative proteomic experiments, phosphatidylglycerophosphate synthase (PGS1) and NADH:ubiquinone oxidoreductase subunit 7 (NDUFA7) were mitochondrial proteins (Figure 4A and Supplemental Table 3). PGS1 is the enzyme responsible for the first step of the synthesis of mitochondria-specific glycerophospholipid CL (28, 29). The PGS1 level was reduced by approximately 45% and 40% in P5 and P10 *Cfap418^–/–^* retinas, respectively (Figure 4A). NDUFA7 is a complex I subunit in the electron transport chain (30) whose protein level was reduced by 34% and 27% in P5 and P10 *Cfap418^–/–^* retinas, respectively (Figure 4A). These data suggest that the mitochondria might be defective in developing *Cfap418^–/–^* retinas. Consistently, GSEA identified that proteins in the mitochondrial translation pathway were reduced in P10 *Cfap418^–/–^* retinas (Supplemental Figure 7A and Supplemental Table 4).

We subsequently analyzed the expression levels of all known mitochondrial proteins in P5 and P10 *Cfap418^–/–^* retinal proteomes, according to the mammalian mitochondrial protein database MitoCarta 3.0 (31). Sixty-six of the mitochondrial proteins were DE in *Cfap418^–/–^* retinas at either P5 or P10 (*P* < 0.05). Proteins that are involved in mitochondrial central dogma (DNA replication, RNA transcription, and protein translation), oxidative phosphorylation, and protein import and sorting were reduced in *Cfap418^–/–^* retinas (Figure 4B). By contrast, proteins involved in lipid metabolism displayed mixed changes (Figure 4B). For example, levels of CPT1A and ACADVL that are involved in the uptake of fatty acids and the first step of fatty acid β-oxidation, respectively, were increased, whereas ACAA2, responsible for the last step of fatty acid β-oxidation, was reduced, indicating a disruption in fatty acid β-oxidation. In summary, these data support the notion that mitochondrial protein translation, oxidative phosphorylation, lipid metabolism, and protein import/sorting are altered in *Cfap418^–/–^* retinas.

We further examined the mitochondrial morphology and structure in *Cfap418^–/–^* photoreceptors using transmission electron microscopy (TEM). At P10, the mitochondria showed a dynamic polymorphous shape in photoreceptors, which seemed slightly more irregular in *Cfap418^–/–^* photoreceptors than in *Cfap418^+/–^* photoreceptors (data not shown). This phenotype became obvious in mature photoreceptors at P21, P28, and P60 (Figure 4C and Supplemental Figure 7B). At these time points, most mitochondria had a straight, long cylindrical shape and were located immediately parallel to the plasma membrane of *Cfap418^+/–^* photoreceptor IS. In *Cfap418^–/–^* littermate photoreceptors, the mitochondria were located normally, and the size and number of the cristae within mitochondria appeared normal (data not shown). However, the mitochondria displayed an irregular cylindrical shape, with bumps and constrictions along their longitudinal axis. Consistently, quantitative analyses of 5–10 photoreceptors in each of the 3 pairs of *Cfap418^+/–^* and *Cfap418^–/–^* littermates at P28–P30 demonstrated that the *Cfap418^–/–^* mitochondria had a large variation in diameter (Figure 4D). The mitochondrial respiration, however, was normal as assessed by the oxygen consumption rate (OCR) in the retinal punches from 10 pairs of *Cfap418^+/+^* and *Cfap418^–/–^* littermate mice at 3–5 weeks of age (Supplemental Figure 7C). Taken together, our results reveal abnormal protein expression in mitochondrial lipid metabolism, oxidative phosphorylation, and protein translation as well as distorted mitochondrial morphology in *Cfap418^–/–^* photoreceptors. Although *Cfap418^–/–^* mitochondrial ultrastructure, cellular location, and function are relatively normal, they may deteriorate with age. Based on the early reductions observed in PGS1 and NDUFA7 at P5, the mitochondrial phenotype is likely another primary defect in *Cfap418^–/–^* photoreceptors.

### Ciliary transport proteins and symporters are altered in Cfap418^–/–^ photoreceptors during OS growth.

**Figure 5:**
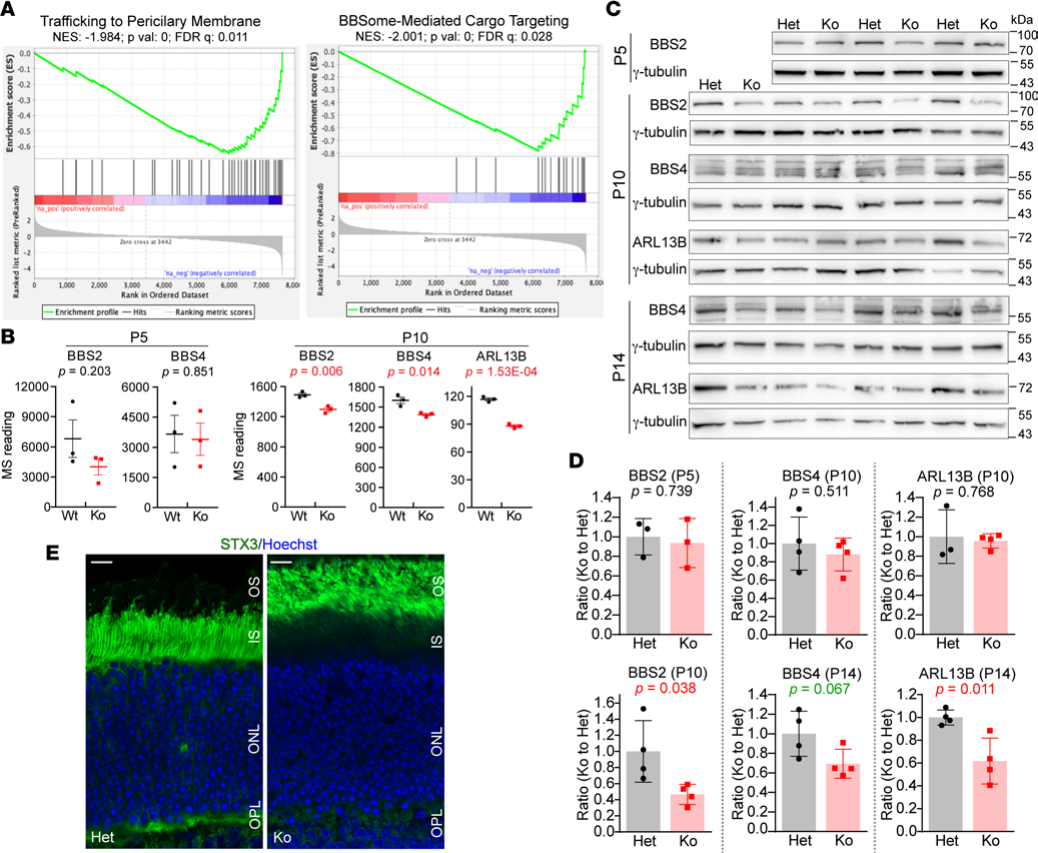
Ciliary transport proteins are affected during *Cfap418^–/–^* OS growth. (**A**) Proteins in ciliary transport pathways are reduced in P10 *Cfap418^–/–^* retinas. (**B**) Quantitative MS data show normal BBS2, BBS4, and undetectable ARL13B (not shown) protein expression in P5 *Cfap418^–/–^* (Ko) retinas and their reduced expression in P10 *Cfap418^–/–^* retinas. (**C**) Semiquantitative immunoblots for BBS2, BBS4, and ARL13B in *Cfap418^+/–^* and *Cfap418^–/–^* littermate retinas at different time points. The corresponding γ-tubulin immunoblots are loading controls. (**D**) Quantification of the semiquantitative immunoblots reveals BBS2 and ARL13B reductions in P10 and P14 *Cfap418^–/–^* retinas, respectively, and a trend of BBS4 reduction in P14 *Cfap418^–/–^* retinas. (**E**) Mislocalization of STX3 from the IS and OPL to the OS in P21 *Cfap418^–/–^* photoreceptors. Scale bars: 10 mm. Data from individual mice and mean ± SEM are shown in **B** and **D** (2-tailed Student’s *t* test). See complete unedited blots in the supplemental material.

During photoreceptor development, the specialized cilium OS grows from the cell body IS from P7 to P21 after ciliogenesis completion (27). In P10 but not P5 *Cfap418^–/–^* retinas, GSEA identified several unique downregulated pathways that are related to OS growth, such as trafficking to the periciliary membrane, ciliary membrane components, and BBSome-mediated ciliary trafficking pathways (Figure 5A and Supplemental Table 4). BBSome components (BBS proteins) and ARL13B protein were involved in these cellular pathways. Quantitative MS data showed that BBS proteins were normal at P5, but BBS2, BBS4, BBS5, BBS7, and ARL13B were reduced at a range of 13% to 25% in *Cfap418^–/–^* retinas at P10 (Figure 5B and Supplemental Table 2). Given the involvement of CFAP418 in BBS pathogenesis (9, 12, 13), we performed semiquantitative immunoblot analysis for BBS2, BBS4, and ARL13B, for which commercial antibodies are available. The BBS2 level was normal at P5 but reduced by approximately 55% at P10 in *Cfap418^–/–^* retinas (Figure 5, C and D). The expression of BBS4 and ARL13B was normal at P10 in *Cfap418^–/–^* retinas, but at P14, ARL13B was reduced by approximately 40%, and BBS4 had a trend of reduction (Figure 5, C and D). Therefore, the results from our quantitative MS and semiquantitative immunoblot analyses were consistent, and the quantitative MS turned out to be more sensitive in detecting protein expression changes. The BBSome is known to mediate the retrograde transport of syntaxin 3 (STX3) from the OS to IS in photoreceptors (32–34). We found that STX3 was mislocalized from the IS and OPL to the OS in *Cfap418^–/–^* photoreceptors at P21 (Figure 5E). Together, our studies showed that the levels of BBS and ARL13B proteins are reduced and the retrograde transport function of the BBSome is affected in *Cfap418^–/–^* retinas during OS growth.

Symporter activity pathway was in a cluster of 45 overlapping upregulated pathways in P10 *Cfap418^–/–^* retinas, revealed by GSEA and EnrichmentMap (35) (data not shown). This pathway had the highest normalized enrichment score (NES) and a close to zero FDR *q* value (Supplemental Figure 8A and Supplemental Table 4). Symporters are transmembrane proteins and cotransport 2 or more ions, amino acids, sugars, lipids, or neurotransmitters in the same direction across cell membranes (36). Forty-six symporters were detected in our P5 and P10 proteomes. Their expression was normal in P5 *Cfap418^–/–^* retinas. However, 11 symporters were increased at P10 (Supplemental Figure 8B). Among them, SLC1A7 and SLC12A2 were previously reported to localize in photoreceptor synaptic terminals (37, 38). Immunostaining for SLC1A1 showed that this symporter was localized in almost all photoreceptor layers at P10 and P21 (Supplemental Figure 8, C and D). SV2 and PMCA1 were used as OPL markers. There was no apparent difference in SLC1A1 distributions between *Cfap418^+/–^* and *Cfap418^–/–^* retinas at P10 and P21 (Supplemental Figure 8C and data not shown). Overall, our results suggest that the expression of some symporters is likely increased during photoreceptor OS growth.

### Synthesis, degradation, and folding of OS membrane proteins are normal in Cfap418^–/–^ photoreceptors.

Our quantitative MS studies demonstrated defects in membrane remodeling–related cell processes and mitochondrial function in *Cfap418^–/–^* photoreceptor IS during ciliogenesis at P5 and BBSome-mediated ciliary trafficking phenotypes during *Cfap418^–/–^* photoreceptor OS growth at P10. However, no clear evidence was found for defects in the machineries of membrane protein synthesis and degradation or endoplasmic reticulum (ER) stress due to protein misfolding in *Cfap418^–/–^* photoreceptors. Because of the widespread reductions in membrane proteins, especially the OS membrane proteins, discovered in *Cfap418^–/–^* photoreceptors (15), we decided to perform pulse and chase experiments to specifically examine OS membrane protein synthesis and degradation, respectively. Pulse labeling using [^35^S] methionine for up to 2 hours revealed no significant reduction of newly synthesized rhodopsin in *Cfap418^–/–^* retinas at P12 compared with a negative control cytoplasmic protein DPYSL2 (Supplemental Figure 9A). Chase labeling using [^35^S] methionine in retinas for 2.5 hours, 3 hours, and overnight showed no evident changes in degradation of rhodopsin or transducin α subunit (GNAT1) at P8 and P10 (Supplemental Figure 9B). Therefore, these pulse-chase results indicate that CFAP418 does not play an essential role in OS membrane protein synthesis or degradation in photoreceptors.

To test whether misfolding of OS membrane proteins occurred and induced ER stress and an unfolded protein response (UPR) in *Cfap418^–/–^* retinas (39), we conducted quantitative reverse transcription PCR (RT-qPCR) and found no changes in the mRNA expression of the 3 UPR pathway markers, ATF6, PERK, and IRE1α, in *Cfap418^–/–^* retinas at P15 and P30 (Supplemental Figure 9C), when the OS membrane protein reduction is robust (15). We then tested whether ATF6 and IRE1α were activated using immunoblot analysis. We observed no cleavage of ATF6 into a 50-kDa fragment, which is the activated form of ATF6, and no elevation of phosphorylated IRE1α, the active form of IRE1α, in *Cfap418^–/–^* retinas at P16 and P30 (Supplemental Figure 9D). Our data suggest that the OS membrane protein reduction in *Cfap418^–/–^* retinas is not due to protein misfolding. Together, our studies specifically on the OS membrane protein synthesis, degradation, and folding using pulse-chase, RT-qPCR, and immunoblot analyses revealed no obvious role of CFAP418 in these processes and corroborated our quantitative MS findings.

### Phosphorylation of proteins, including protein kinase Cα, is altered in developing Cfap418^–/–^ retinas.

**Figure 6:**
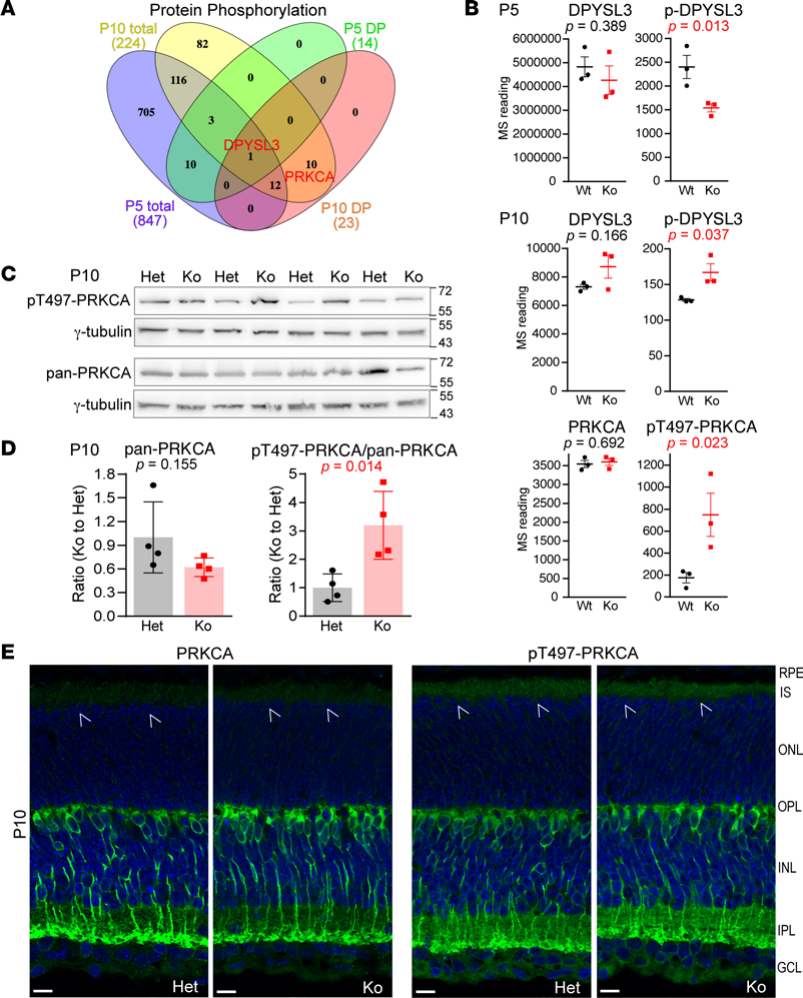
Protein phosphorylation is altered in developing *Cfap418*^–/–^ photoreceptors. (**A**) DPYSL3 is the only differentially phosphorylated protein identified in both P5 and P10 *Cfap418^–/–^* retinas, while PRKCA is a differentially phosphorylated protein identified in P10 *Cfap418^–/–^* retinas. (**B**) Quantitative MS demonstrates that DPYSL3 phosphorylation is reduced in P5 *Cfap418^–/–^* (Ko) retinas, and DPYSL3 and PRKCA phosphorylation is increased in P10 *Cfap418^–/–^* retinas. (**C**) Semiquantitative immunoblots for pan- and pT497-PRKCA in retinas from 4 pairs of P10 *Cfap418^+/–^* and *Cfap418^–/–^* littermate mice. γ-Tubulin is a loading control. (**D**) Quantification of the semiquantitative immunoblots for pan- and pT497-PRKCA signals. (**E**) Immunostaining displays similar pan- and pT497-PRKCA signal patterns between P10 *Cfap418^+/–^* and *Cfap418^–/–^* littermate retinas. The pT497-PRKCA signal is stronger in photoreceptors than the pan-PRKCA signal (arrows). Scale bars: 10 mm. Data are presented as individual mice and mean ± SEM in **B** and **D** (2-tailed Student’s *t* test). See complete unedited blots in the supplemental material.

To thoroughly characterize the molecular phenotypes in *Cfap418^–/–^* retinas during development, we also analyzed protein phosphorylation in our P5 and P10 quantitative proteomic MS data (Supplemental Tables 1 and 2). Ddihydropyrimidinase-related protein 3 (DPYSL3) was the only protein that was differentially phosphorylated (DP) in *Cfap418^–/–^* retinas discovered at both P5 and P10 (Figure 6A). Compared with *Cfap418^+/+^* retinas, DPYSL3 phosphorylation was reduced by approximately 35% at P5 but increased by approximately 30% at P10, while the total DPYSL3 expression level was normal at both P5 and P10 (Figure 6B). The DP site of DPYSL3 at P10 was serine 101 (NP_001278384), which had not been well characterized so far.

At P10, the phosphorylation of protein kinase Cα at threonine 497 (pT497-PRKCA) was increased 4.3-fold in *Cfap418^–/–^* retinas, while the total PRKCA expression was unchanged, compared with *Cfap418^+/+^* littermate retinas (Figure 6B). This phosphorylation site is critical for PRKCA activation (40) and was the only DP site that had an antibody commercially available among all the identified DP proteins/sites at P5 and P10. Semiquantitative immunoblot analysis was conducted. After normalization by loading control signals from γ-tubulin, the ratio of pT497-PRKCA to pan-PRKCA signal was increased 3.2-fold in *Cfap418^–/–^* retinas compared with *Cfap418^+/–^* retinas (Figure 6, C and D). Because PRKCA was localized only to the rod bipolar cells in mature rodent retinas (Supplemental Figure 10) (41, 42), we examined PRKCA distribution in P5 and P10 mouse retinas by immunostaining. Both PRKCA and pT497-PRKCA were localized throughout different retinal layers at P5 (Supplemental Figure 10). At P10, PRKCA and especially pT497-PRKCA were present in photoreceptor IS and rod bipolar cells (Figure 6E). Their signal patterns were the same between *Cfap418^+/–^* and *Cfap418^–/–^* retinas (Figure 6E). Therefore, PRKCA’s activity, indicated by its phosphorylation at T497, is enhanced probably in both *Cfap418^–/–^* photoreceptors and rod bipolar cells during development. Because PRKCA signaling occurs at the cellular membranes (43), our findings suggest that CFAP418 deficiency affects membrane-associated cell signaling in the retina.

### CFAP418 interacts weakly with RAB28 in the retina.

**Figure 7:**
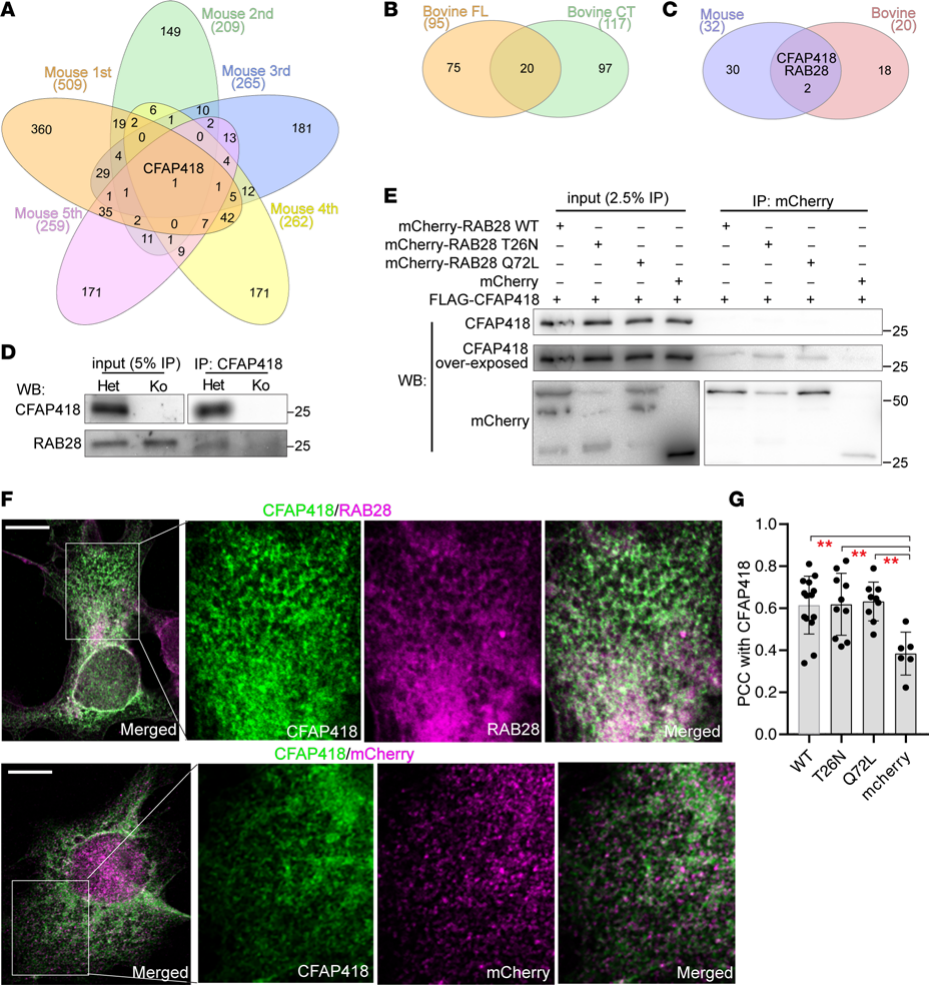
CFAP418 interacts transiently with RAB28 in retinas. (**A**) Overlap of proteins identified from 5 AP-MS experiments using a CFAP418 antibody and mouse retinas at P30. (**B**) Overlap of proteins identified from 2 AP-MS experiments using CFAP418 recombinant proteins and adult bovine retinas. (**C**) Overlap of potential CFAP418-interacting proteins identified from mouse and bovine retinas. (**D**) RAB28 was coimmunoprecipitated with CFAP418 from mouse retinas at 1 month of age. (**E**) mCherry-RAB28 proteins, but not mCherry, pulled down a small fraction of FLAG-CFAP418 in HEK293 cells. Note that FLAG-CFAP418 was only seen in the overexposed immunoblot. (**F**) FLAG-CFAP418 was colocalized with mCherry-RAB28 but not with mCherry. Framed regions are amplified and shown on the right. Scale bars: 10 mm. (**G**) The Pearson’s correlation coefficients (PCCs) of FLAG-CFAP418 with mCherry-RAB28 WT, T26N, Q72L, and mCherry alone. Data are represented as individual cells, mean, and SEM. **P < 0.01 (1-way ANOVA with Tukey’s multiple-comparison test). See complete unedited blots in the supplemental material.

To understand the molecular mechanism underlying the CFAP418 function and the phenotypes in *Cfap418^–/–^* retinas, we attempted to identify CFAP418-interacting proteins using AP-MS (Supplemental Tables 5 and 6). Hundreds of proteins were coimmunoprecipitated with CFAP418 from *Cfap418^+/–^* mouse retinas in each of the 5 replicate experiments (Figure 7A). Non-immunoglobin and *Cfap418^–/–^* littermate retinas were included in these experiments as negative controls. However, no protein was coimmunoprecipitated with CFAP418 in all 5 biological replicates, indicating that CFAP418 may form a transient and weak interaction with proteins. We also performed GST pulldown experiments from bovine retinal lysates using GST-tagged mouse CFAP418 full-length (FL) and C-terminal (CT) baits. The CT bait (169–209 aa, NP_080281), encoded by exon 6, is evolutionarily conserved in sequence and contains many pathogenic mutations identified in patients (Supplemental Figure 1A) (12). Twenty proteins were pulled down by both CFAP418 FL and CT baits, but not by GST (Figure 7B). We compared these 20 proteins with the 32 proteins that were coimmunoprecipitated with CFAP418 at least 3 times from mouse retinas (Figure 7C). Only 2 proteins were shared between the 2 pools, CFAP418 and RAB28.

RAB28 was found to decrease in both P5 and P10 *Cfap418^–/–^* retinas by our quantitative proteomic studies (Figure 1D). Additionally, mutations in *CFAP418* (10, 44, 45) and *RAB28* (46–48) both lead to cone-rod dystrophy in patients. We thus investigated the potential interaction between CFAP418 and RAB28. Consistent with our AP-MS results, we detected RAB28 in the CFAP418 immunoprecipitate from mouse retinas in 1 of 3 coimmunoprecipitation experiments (Figure 7D). We then tested the direct interaction between RAB28 and CFAP418 and assessed whether this interaction depended on the GTP/GDP-binding status of RAB28. We cotransfected HEK293 cells with FLAG-CFAP418 and mCherry-RAB28 WT, RAB28 T26N (GDP-bound), or RAB28 Q72L (GTP-bound). All WT and mutant mCherry-RAB28 proteins, but not mCherry protein (negative control), pulled down a small fraction of CFAP418 (~0.1%), and GDP-RAB28 appeared to pull down more CFAP418 (Figure 7E) than other RAB28 proteins. Considering that CFAP418 and a small fraction of RAB28 are present in photoreceptor IS (15, 17), these 2 proteins probably interact in this photoreceptor compartment. Because our CFAP418 antibodies could not detect the endogenous CFAP418 protein in photoreceptors by immunostaining and the volume of mouse photoreceptor IS is too small to examine detailed protein distribution, we examined the colocalization between CFAP418 and RAB28 in double-transfected COS-7 cells (Figure 7F and Supplemental Figure 4B). Pearson’s correlation coefficients (PCCs) between CFAP418 and RAB28 WT, T26N, and Q72L were similar to each other but significantly higher than the PCC between CFAP418 and mCherry (Figure 7G). The significant colocalization and weak interaction between CFAP418 and RAB28 suggest an indirect or transient association between these 2 proteins in photoreceptors.

### CFAP418 binds to PA and CL in cell membranes.

**Figure 8:**
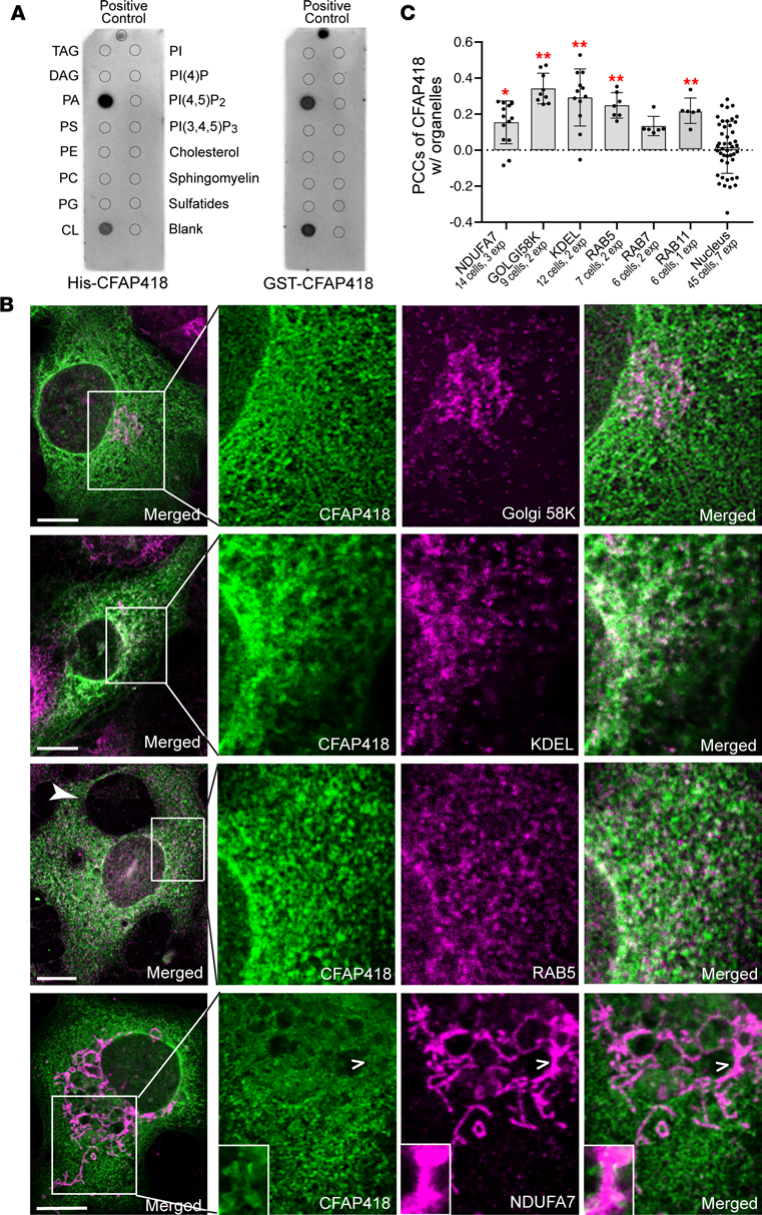
CFAP418 binds to PA and CL in various cell membranes. (**A**) Purified recombinant His- and GST-CFAP418 proteins bind directly to PA and CL on membrane strips. The lipid arrangements on the 2 membrane strips are the same. Refer to the full lipid names in the Results section. (**B**) Representative immunostaining results for FLAG-CFAP418 and various cell organelle markers in COS-7 cells. The filled arrowhead denotes a large vacuole formed in the transfected cell. The open arrowheads point to the position where the amplified insets are located. The insets show CFAP418 is present at mitochondrial edges. Scale bars: 10 mm. (**C**) The Pearson’s correlation coefficients (PCCs) of CFAP418 with different cell organelle markers. Nuclear dye Hoechst 33342 was used as a negative control. Data are represented as individual cells, mean, and SEM. *P < 0.05, **P < 0.01 (1-way ANOVA with Tukey’s multiple-comparison test versus the nucleus group).

The scarcity of strong and stable interactions between CFAP418 and proteins and the abundance of cell membrane–associated phenotypes caused by *Cfap418* deficiency prompted us to investigate the potential interactions of CFAP418 with membrane lipids. Both His- and GST-tagged mouse CFAP418 proteins were found to bind to PA and CL on membrane strips (Figure 8A and Supplemental Figure 11A). His-CFAP418 also bound weakly to lysophosphatidic acid (LPA) (Supplemental Figure 11A). These bindings were specific, because His- and GST-CFAP418 did not bind to other glycerophospholipids (phosphatidylcholine [PC], lysophosphatidylcholine [LPC], phosphatidylethanolamine [PE], phosphatidylserine [PS], phosphatidylglycerol [PG], phosphatidylinositol [PI], and phosphoinositides [PIPs]), glycerolipids (triglyceride [TAG] and diacylglycerol [DAG]), sterol (cholesterol), or sphingolipids (sphingomyelin, sphingosine-1 phosphate, and sulfatide) on the strips (Figure 8A and Supplemental Figure 11A).

**Figure 9:**
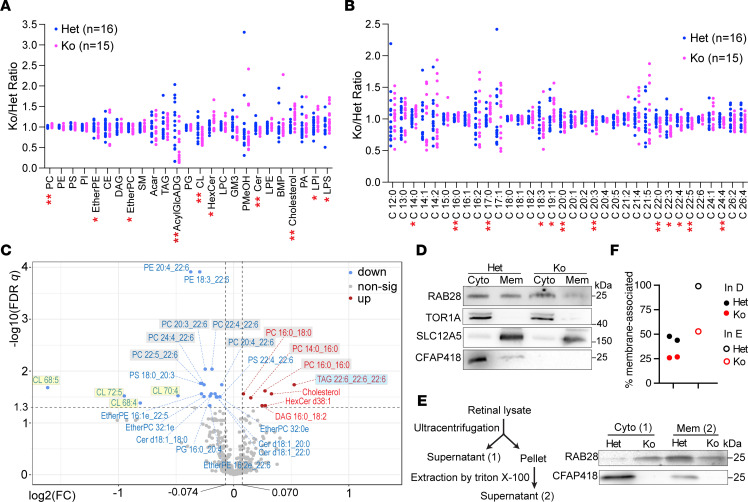
Abnormal membrane lipid composition and membrane-protein association in developing *Cfap418^–/–^* retinas. (**A**) Membrane lipid categories affected in P10 *Cfap418^–/–^* (Ko) retinas. (**B**) Acyl chains affected in P10 *Cfap418^–/–^* retinal membrane lipids. (**C**) Volcano plot showing fold changes of individual lipid species between *Cfap418^+/–^* and *Cfap418^–/–^* retinas at P10. (**D** and **E**) RAB28 is increased in the cytosol (Cyto) and decreased in the membrane (Mem) in *Cfap418^–/–^* retinas, compared with *Cfap418^+/–^* retinas at P21 (using a commercial membrane protein extraction kit, **D**) and P30 (using a Triton X-100 protocol, **E**). TOR1A and SLC12A5 blots were used to verify the separation between cytosolic and membrane fractions. (**F**) Quantification of the percentage of RAB28 present in the membrane fraction. Dot plots in **A** and **B** show data from individual mice. *P < 0.05, **P < 0.01 (2-tailed Student’s *t* test). The dot plot in **F** shows data from independent experiments. See complete unedited blots in the supplemental material.

PA is present in most cell membranes, and CL is exclusively found in mitochondrial membranes (3, 49). During our separation of cytosolic and membrane-bound proteins, we observed a small amount of CFAP418 existing in the membrane fraction from *Cfap418*^+/–^ retinas (Figure 9, D and E). Additionally, FLAG- and GFP-CFAP418 proteins appeared on the intracellular membranes and plasma membrane when transfected in COS-7 and HEK293 cells (Figure 8B and data not shown). To investigate which intracellular membranes CFAP418 associated with, we compared the distribution of transfected FLAG-CFAP418 with those of ER (KDEL), Golgi (Golgi 58K), mitochondrial (NDUFA7), and endosomal (RAB5, RAB7, and RAB11) markers in subconfluent COS-7 cells. The PCCs of CFAP418 with ER and Golgi apparatus markers were higher than those with endosomal and mitochondrial markers (Figure 8, B and C). Although the PCC between CFAP418 and mitochondrial marker NDUFA7 was relatively low, we detected an overlap between CFAP418 and NDUFA7 signals at the edge of mitochondria (open arrowheads and insets in Figure 8B), implying CFAP418 may interact with CL at the mitochondrial outer membrane where approximately 3% of CL exists (49, 50). As a control, the PCC of CFAP418 with a nuclear dye was around zero, consistent with our previous observation that CFAP418 is absent in the nucleus (15). Together, our data demonstrate that CFAP418 binds to PA and CL in various cell membranes, including the ER, Golgi, endosome, and mitochondrial outer membranes.

### Cfap418 deletion disrupts the membrane lipid composition in developing retinas.

To examine whether and what membrane lipids were affected in *Cfap418^–/–^* retinas, we performed a quantitative untargeted lipidomic analysis in the retinas of 16 *Cfap418^+/–^* and 16 *Cfap418^–/–^* littermate mice at P10. In total, 408 lipid species from 25 categories were detected (Supplemental Table 1). The lipid composition in *Cfap418^+/–^* retinas was consistent with that previously reported in vertebrate retinas (Supplemental Figure 11, C and D) (51). In *Cfap418^–/–^* retinas, we observed enrichment of aggregated PC, lysophosphatidylserine (LPS), lysophosphatidylinositol (LPI), hexosylceramides (HexCer), and cholesterol (Figure 9A). In contrast, aggregated EtherPC, EtherPE, acylglucuronosyldiacylglycerols (AcylGlcADG), CL, and ceramides (Cer) were depleted (Figure 9A). Cholesterol was the most increased, with a ratio of *Cfap418^–/–^* to *Cfap418^+/–^* abundance (FC) at 1.26 ± 0.06 (mean ± SEM, *P* = 0.00086). CL, as a group of CFAP418-binding lipids, was the second most reduced lipid category (FC = 0.75 ± 0.03, *P* = 0.0010), which was consistent with the PGS1 expression reduction in *Cfap418^–/–^* retinas at P5 and P10. Within the CL category, CL 68:4, CL 68:5, CL 70:4, and CL 72:5 were significantly reduced (FC < 0.95, FDR *q* < 0.05; Figure 9C and Supplemental Table 2, the X:Y in the lipid nomenclature indicates the carbon and double bond numbers in the acyl chains, respectively). Metabolite set enrichment analysis (MSEA) on the quantitative lipidomic profiles without any cutoff thresholds similarly found that the lipid changes were enriched in the CL (FDR = 0.0117), cholesterol (FDR = 0.0117), and PC (FDR = 0.0117) categories in *Cfap418^–/–^* retinas.

Further analysis revealed that shorter and more saturated acyl chains were increased (i.e., C14:0, C16:0, C17:0, and C19:1) and longer and more unsaturated acyl chains were reduced (i.e., C18:3, C20:0, C20:3, C22:0, C22:3, C22:4, C22:5, and C24:4) in *Cfap418^–/–^* membrane lipids (Figure 9B). This phenotype was more evident in the altered PC species in *Cfap418^–/–^* retinas (Figure 9C and Supplemental Figure 11E). PC 14:0_16:0, PC 16:0_16:0, and PC 16:0_18:0 were increased, while PC 20:4_22:6, PC 22:4_22:6, PC 22:5_22:6, and PC 24:4_22:6 were decreased. In addition to the affected CL, PC, and cholesterol species mentioned above, 6 other glycerophospholipids, 4 ether glycerophospholipids, and 3 sphingolipids were reduced, and 2 glycerolipids and 1 sphingolipid were increased in *Cfap418^–/–^* retinas (Figure 9C and Supplemental Table 2). Because PA is rare in membrane lipids (3, 52), we detected only 1 PA species in our lipidomic study, which was unaffected in *Cfap418^–/–^* retinas (Figure 9A). Altogether, these data demonstrate a widespread change in membrane lipid composition associated with the disruption of CFAP418 interactions with PA and CL in developing retinas.

### Lipid metabolic enzyme and transporter expressions are altered in developing Cfap418^–/–^ retinas.

To investigate how disruption of CFAP418’s interactions with PA and CL resulted in widespread changes in different membrane lipid categories and acyl chains, we surveyed the expression of membrane lipid metabolic enzymes and transporters. One hundred eighty-six of these proteins (4) were detected in our P5 and P10 retinal proteomes. In P5 *Cfap418^–/–^* retinas, no membrane lipid metabolic enzymes or transporters showed changes in expression, except the above-mentioned mitochondrial enzyme PGS1 (Figure 4A). However, at P10, besides PGS1, 12 additional membrane lipid metabolic enzymes and transporters were DE (Supplemental Figure 12). Some of the changes may explain the membrane lipid changes we observed. For example, ACSF2 and SLC27A3 are medium-chain and very-long-chain acyl-CoA synthetases in mitochondria, respectively (53, 54). They function in the de novo synthesis and remodeling of acyl chains in glycerophospholipids (4, 55). The ACSF2 increase and the SLC27A3 reduction may lead to the opposite changes in short and long acyl chains in *Cfap418^–/–^* glycerophospholipids. PLA2G15 functions as both phospholipase A2 and ceramide acyltransferase toward PC, PE, PS, and PG (56, 57). Its increase may explain the LPS and LPI increases and the subsequent acyl chain remodeling. The increases in TAG transporter LPL (58) and TAG synthetase DGAT1 (59, 60) may contribute to the increase in TAG 22:6_22:6_22:6 in *Cfap418^–/–^* retinas. The reduction in SMPD2, a sphingomyelinase for ceramide generation (61), may be responsible for the ceramide reduction in *Cfap418^–/–^* retinas. Furthermore, MTMR2, PLCD1, and PIK3CA participate in PIP metabolism and signaling (62–65). Their changes may affect the PIP abundances in *Cfap418^–/–^* retinas, which were not detected in our lipidomic study due to the extremely low PIP abundance in retinal tissues (66). Because most DE lipid metabolic enzymes and transporters were affected at P10 but not P5, their expression changes are likely secondary to the disrupted CFAP418 lipid binding and may contribute to the widespread membrane lipid changes in *Cfap418^–/–^* retinas.

### Cfap418 deficiency affects the association of proteins with cell membranes.

To study whether the disruption of CFAP418 lipid binding and membrane lipid composition affected membrane-protein associations, we focused on RAB28, which was identified as a weak CFAP418-interacting partner and a DE protein in *Cfap418^–/–^* retinas at both P5 and P10 (Figure 1D and Figure 7, D–G). RAB28 is known to associate with membranes through prenylation (67). We separated proteins in retinal cytosol and membranes using a commercial membrane protein extraction kit at P21 (Figure 9D) and a Triton X-100 extraction protocol at P30 (Figure 9E). RAB28 protein was present in both retinal cytosol and membrane fractions in these 2 experiments. Compared with *Cfap418*^+/–^ littermate retinas, the amount of RAB28 protein in membranes was reduced by approximately 50% in *Cfap418^–/–^* retinas (Figure 9F). This result suggests that CFAP418 lipid binding and its maintenance of membrane lipid homeostasis are important for regulating the membrane-protein associations in photoreceptors.

## Discussion

Using multiple unbiased omics approaches combined with traditional biological methodology, we unexpectedly discovered that CFAP418 binds prominently to PA and CL on cell membranes but not strongly to specific proteins. The disruption of CFAP418 lipid binding results in extensive changes in membrane lipid composition and weakens RAB28 membrane association in the retina. During ciliogenesis in photoreceptors, *Cfap418* knockout reduces CL synthase PGS1 and membrane remodeling–associated proteins, which is followed by compromised mitochondrial morphology and mislocalization of ESCRT-0 proteins. During photoreceptor OS growth, *Cfap418* knockout reduces ciliary transport BBS and ARL13B proteins, disturbs BBSome function, increases transmembrane symporters, and activates PA-binding signaling protein PRKCA. Although CFAP418 is considered a cilia- and flagella-associated protein, our data indicate that this protein may contribute indirectly to ciliogenesis through exerting functions in nonciliary cellular compartments. This study sheds light on the multiple roles of membrane lipid homeostasis in cells, which has been severely understudied, and demonstrates that disruption of the membrane lipid homeostasis is a pathological mechanism underlying IRDs and syndromic ciliopathies. We further show that the application of complementary omics approaches with targeted in vivo phenotypic characterization is an effective way to interrogate the molecular function of poorly characterized disease-causing genes, especially the genes that are related to membrane lipid homeostasis. Our findings open an avenue to further mechanistic studies on membrane lipid biology, ciliogenesis, ciliopathies, and other lipid-related genetic diseases.

PA, as a CFAP418-interacting lipid, is a precursor of many glycerophospholipids in membranes and glycerolipids in lipid droplets inside cells (52). Thus, PA is a switch point between membrane biogenesis and lipid storage (68). The reduction in many glycerophospholipids and enrichment in some glycerolipids observed in *Cfap418^–/–^* retinas (Figure 9C) suggest an imbalance between membrane biogenesis and lipid storage. The widespread changes across different lipid categories in *Cfap418^–/–^* retinas are probably due to the interconnection of the metabolic pathways of different lipid categories (4). These changes are expected to affect the membrane biophysical and biochemical properties and the abundance and function of many integral and peripheral membrane proteins. This notion is supported by our findings of decreased RAB28 membrane association and altered HGS, symporter, and photoreceptor OS membrane protein expression in *Cfap418^–/–^* retinas. The cone-shaped PA has a small head group and actively participates in membrane fission and fusion through induction of a negative membrane curvature (3). Consistently, we observed membrane remodeling phenotypes in both CFAP418-overexpressing cells and CFAP418-knockout photoreceptors. In line with previous knowledge that PA directly interacts with proteins to regulate protein conformation and function (3, 69), we discovered changes in the activities of PA-binding BBSome and PRKCA in *Cfap418^–/–^* retinas. Additionally, CL, another CFAP418-binding lipid, is present only in mitochondrial membranes, where it is synthesized from and hydrolyzed to PA (52). It is known that both PA and CL play a role in mitochondrial dynamics and function (49, 70). In agreement with this information, we found that disruption of the interactions between CFAP418 and PA/CL causes mitochondrial morphological and protein expression defects in *Cfap418^–/–^* photoreceptors, although the mitochondrial OCR, structure, and cellular location are normal at an early stage. Therefore, our findings in this study support the hypothesis that CFAP418 binds to membrane lipids and plays an essential role in membrane lipid homeostasis.

Mechanistically, CFAP418 could sequester PA and CL in specific membrane domains, regulate PA and CL synthesis and their product generation, and/or modulate the PA and CL roles in maintaining protein conformations and activities. There is no consensus protein sequence for binding PA or CL, although hydrophobic and positively charged protein residues are proposed to participate in PA and CL binding (3, 69, 71, 72). A hydrogen bond switch model has been proposed for PA binding (3). CFAP418 is enriched with positively charged residues. But the mechanisms and modes of CFAP418 binding to PA and CL are unknown. The binding of CFAP418 to PA and CL could be independent or positively or negatively cooperative. It is also possible that CFAP418 binds only to some specific PA and CL species. Future studies to address these questions will be key to the in-depth mechanistic understanding of the CFAP418 function.

Based on our observations that CFAP418 interacts more favorably with membrane lipids than proteins, we propose that the membrane lipid defects occur upstream of the protein expression and phosphorylation changes in *Cfap418^–/–^* retinas. To support this idea, we found that the expression of lipid metabolic enzymes and transporter proteins is normal during ciliogenesis and becomes altered later at P10 in *Cfap418^–/–^* retinas. Additionally, although some membrane remodeling–related and mitochondrial proteins are reduced as early as P5, no evidence in our multiple AP-MS experiments suggests that CFAP418 interacts with these proteins (Supplemental Tables 3 and 5). The partial colocalization of CFAP418 with VPS4B, HGS, and STAM shown in this study could be mediated by membrane lipids, such as PA and CL. Therefore, the expression changes in membrane remodeling–related and mitochondrial proteins during ciliogenesis in *Cfap418^–/–^* retinas are likely secondary to the membrane lipid defects. To corroborate our hypothesis, an examination on the membrane lipid composition in *Cfap418^–/–^* retinas at early time points is necessary.

The photoreceptor OS phenotypes are the most evident in mature *Cfap418^–/–^* retinas (15). Here, we discovered several defects during OS growth that contribute to the phenotypes. The BBSome complex and ARL13B protein are known to transport membrane proteins into and out of the photoreceptor OS (33, 73–76). We found reductions in BBS and ARL13B proteins and dysfunction of the BBSome in developing *Cfap418^–/–^* photoreceptors. RAB28 and STX3 are 2 BBSome cargos and play a role in cone OS shedding and OS membrane protein trafficking, respectively (17–19, 32, 77). We observed a reduction in RAB28 expression and membrane association and the mislocalization of STX3 in developing *Cfap418^–/–^* photoreceptors. Furthermore, the endosomal pathway was recently reported to be implicated in the transport of photoreceptor OS proteins (78). In particular, HGS participates in the OS trafficking of peripherin in cones (78). PRKCA is known to participate in endocytic membrane trafficking and can be regulated by membrane lipids other than DAG (43, 79). Therefore, the combined defects in the BBS, ARL13B, RAB28, STX3, HGS, STAM, and PRKCA proteins during OS growth could contribute to the OS membrane alignment and protein expression phenotypes in mature *Cfap418^–/–^* photoreceptors (15). However, the function of most DE symporters in developing photoreceptors has not been studied and the physiological relevance of their changes in *Cfap418^–/–^* photoreceptors remains to be elucidated.

The removal of docosahexaenoic acid (DHA, 22:6) in glycerophospholipids was previously shown to disrupt photoreceptor OS disk alignment (80), a phenotype similar to what we saw in *Cfap418^–/–^* photoreceptors (15). DHA also affects STX3 conformation and function in photoreceptors (81). Moreover, the center and rim of photoreceptor OS disks are differentially enriched in PC and PE and short saturated and long polyunsaturated acyl chains (82). Therefore, the reductions in many DHA-containing glycerophospholipids, the changes in various PC and PE species, and the abnormal ratio of shorter saturated to longer polyunsaturated acyl chains could contribute directly to the OS membrane disk phenotypes in *Cfap418^–/–^* photoreceptors. Furthermore, some lipid metabolic defects (Supplemental Figure 12) may explain the protein expression and activity changes in *Cfap418^–/–^* photoreceptors. For example, HGS binds to PI(3)P via its FYVE domain (22). In *Cfap418^–/–^* retinas, the reduction in PIK3CA, which phosphorylates PI to generate PI(3)P, and the increase in MTMR2, which dephosphorylates PI(3)P to generate PI, are expected to decrease PI(3)P perhaps in photoreceptor IS. These changes may lead to the HGS mislocalization to photoreceptor OS, where the disks are enriched with PI(3)P (83). The increase in PRKCA activity may be related to the increases in PLCD1 expression and DAG abundance in *Cfap418^–/–^* retinas. While these connections between lipid and protein defects need to be further verified in *Cfap418^–/–^* photoreceptors, it would be interesting to understand how the affected lipids and proteins coordinate and function together to ensure the proper vesicular trafficking, especially the ciliary trafficking of OS integral and peripheral membrane proteins, in healthy photoreceptors.

The combined application of quantitative lipidomic, proteomic, phosphoproteomic, and bioinformatic approaches (35, 84–87) enabled us to systematically integrate the *Cfap418^–/–^* phenotypes from different angles. The use of a large sample size in our untargeted lipidomic study substantially increased the sensitivity to detect changes in lipid species. Some of these changes can be explained by the altered expression of membrane lipid metabolic enzymes and transporters revealed by our proteomic data, although the activity changes of these proteins need to be further analyzed. Supportively, the increase in cholesterol and decrease in DHA-containing PC discovered in our *Cfap418^–/–^* retinas were also found in the photoreceptor OS of 5-month-old *bbs1^–/–^* zebrafish (88), a BBS model at a later disease progression time point. In addition to the reliability, objectivity, and broad coverage, our quantitative proteomics approaches are more sensitive than traditional semiquantitative immunoblot analyses and can detect moderate protein expression and phosphorylation changes. These changes generally complement each other and some of them are confirmed by independent techniques or highly consistent with known *Cfap418^–/–^* phenotypes. Besides the 8 DE proteins mentioned in this report, 5 additional DE proteins are shared in P5 and P10 proteomes (Supplemental Table 3). The membrane association of these proteins and the physiological significance of their changes remain unclear. Furthermore, hundreds of other DE proteins, tens of DP proteins, and tens of altered molecular functions and cellular pathways were identified in this study (Supplemental Tables 2 and 4). A detailed investigation of these alterations will enable us to depict a more complete portrait of the function of CFAP418 and membrane lipid homeostasis in cell membrane and vesicular transport pathways. Among the known proteins associated with IRD diseases (RetNet; https://web.sph.uth.edu/RetNet/), 25 DE proteins were found in P5 and/or P10 *Cfap418^–/–^* retinas (Supplemental Figure 13). Therefore, our omics data are valuable resources to generate hypotheses for proteins and cellular pathways related to CFAP418 in human physiological and pathological conditions. However, because rod photoreceptors are dominant in mouse retinas, compared with cones and other retinal neurons, our multiomics approach may miss changes occurring in those cells.

In summary, our findings demonstrate that CFAP418 is a lipid-binding protein involved in maintaining lipid homeostasis in many cellular membranes during development and that the integrity of cellular membranes is essential for mitochondrial morphology and perhaps function, nonciliary and ciliary vesicular trafficking pathways, and membrane-associated signaling. Our studies reveal the pathogenic mechanism underlying the defects in photoreceptors and perhaps other ciliated cells caused by *CFAP418* mutations and suggest that this mechanism is partially shared with other IRD genes, e.g., the BBS and RAB28 genes. Considering its high evolutionary conservation, expression across many developing and mature tissues (9, 12), involvement in melanosome transport in nonciliated zebrafish melanophores (12), and BBS phenotypes in multiple organ systems (9, 12, 13), the role of CFAP418 in lipid binding, homeostasis, and membrane remodeling is likely conserved and important for cell survival and function across tissues and species.

## Methods

### Mice and cell lines.

*Cfap418^–/–^* mice with a mixed CBA/C57BL6 genetic background have been described previously (15). Mice of both sexes were randomly assigned to experimental groups at various time points. Littermate *Cfap418*^+/+^ or *Cfap418*^+/–^ mice were included as controls. HEK293 (CRL-10852) and COS-7 (CRL-1651) cell lines were purchased from ATCC (https://www.atcc.org). These cells were cultured in Dulbecco’s modified Eagle’s medium (DMEM) supplemented with 10% (v/v) fetal bovine serum, 50 U/mL penicillin, and 50 mg/mL streptomycin (Thermo Fisher Scientific). The One Shot BL21 Star (DE3) cell line was purchased from Thermo Fisher Scientific (C601003) and cultured according to the manufacturer’s protocol.

### Label-free and TMT-labeling quantitative MS, AP-MS, and lipidomic MS.

These experiments were conducted by the Mass Spectrometry Proteomics Cores using standard protocols at Baylor College of Medicine, Harvard Medical School, and the University of Utah, respectively. Details are described in the Supplemental Methods.

### Other molecular, cellular, biochemical, TEM, and Seahorse methods.

Experiments using these methods followed either our previously described protocols (15, 89) or the manufacturers’ protocols. For detailed descriptions, please see the Supplemental Methods.

### Statistics.

Protein and phosphoprotein levels generated from label-free and TMT-labeling MS were processed using the Statistical Analysis module in MetaboAnalyst 5.0 (87, 90). The 2-tailed Student’s *t* test in the univariate analysis category was conducted to identify DE and DP proteins. We defined DE and DP proteins as those with raw *P* values smaller than 0.05. There was no FC cutoff for DE proteins at P5 or DP proteins at P5 or P10, while the FC cutoff was 1.1 for the DE proteins at P10. Lipidomic data were also processed and analyzed in MetaboAnalyst 5.0 (87, 90). PCA and heatmap hierarchical clustering detected sample 14 in the *Cfap418^–/–^* group as an outlier (Supplemental Figure 11B and data not shown). This sample was excluded from downstream analyses. Differentially abundant lipid species were identified by Student’s *t* test with an FDR value smaller than 0.05 and a ratio of *Cfap418^–/–^* to *Cfap418^+/–^* of larger than 1.05 or smaller than 0.95. The results of RT-qPCR and semiquantitative immunoblot analyses, the abundances of aggregated lipid categories and acyl chains, and mitochondrial diameter and diameter variation were compared between *Cfap418^–/–^* and control (*Cfap418^+/+^* or *Cfap418^+/–^*) groups using Student’s *t* test in Microsoft Excel. Details of statistical analyses are described in the Supplemental Methods.

### Study approval.

All mice were maintained, cared for, and examined according to the protocol approved by the Institutional Animal Care and Use Committee at the University of Utah.

### Data availability.

The MS proteomic and phosphoproteomic data have been deposited in the ProteomeXchange Consortium (http://www.proteomexchange.org) via the MASSIVE repository with the data set identifiers MSV000089334 (P5) and MSV000089424 (P10). The MS lipidomic data have been deposited in the Metabolomics workbench (https://www.metabolomicsworkbench.org) (91) with the study identifier ST002162. All other data are available in the Supporting Data Values XLS file. No code was generated in this study.

## Author contributions

AMC and DY designed and performed the experiments. DZ, GN, JZ, JAM, and TB performed the experiments. JY designed the experiments, analyzed the data, and wrote the manuscript.

## Supplementary Material

Supplemental data

Supplemental table 1

Supplemental table 2

Supplemental table 3

Supplemental table 4

Supplemental table 5

Supporting data values

## Version 1 11/16/2023

In-Press Preview

## Version 2 01/09/2024

Electronic publication
