# Supplementary material for: Disruption of CFAP418 interaction with lipids causes widespread abnormal membrane-associated cellular processes in retinal degenerations
Source: JCI Insight. 2024 Jan 9;9(1):e162621. doi: 10.1172/jci.insight.162621 (PMC10906455; doi:10.1172/jci.insight.162621)

Supplemental Material

KEY RESOURCES TABLE

REAGENT OR RESOURCE	SOURCE	IDENTIFIER
**Antibodies**		
Rhodopsin (1D4)	Robert Molday, University of British Columbia, Vancouver, Canada (1)	
DPYSL2	This paper	
GNAT1	Santa Cruz	sc-389; RRID:AB_2294749
ATF6	NovusBiologicals	70B1413.1
Phospho-IRE1α	NovusBiologicals	NB100-2323; RRID:AB_10145203
γ-tubulin	Sigma-Aldrich	T6557; RRID:AB_477584
RAB28	Biorbyt	Orb11632
CFAP418	Custom-made (2)	
FLAG	Sigma-Aldrich	F1804; RRID:AB_262044
KDEL	Abcam	ab12223; RRID:AB_298945
Golgi58K	Abcam	ab6284; RRID:AB2041471
RAB5A	CellSignalingTech	46449; RRID:AB_2799303
RAB7	CellSignalingTech	9367; RRID:AB_1904103
RAB11	CellSignalingTech	5589; RRID:AB_10693925

EEA1	ThermoFisher	MA5-14794;<br>RRID:AB_10985824
NDUFA7	NovusBiologicals	NBP3-03240
STAM	Proteintech	12434-1-AP;<br>RRID:AB_2199965
HGS	ThermoFisher	PA5-27491;<br>RRID:AB_2544967
VPS4B	Proteintech	17673-1-AP;<br>RRID:AB_2215504
BBS2	Santa Cruz	Sc-365355;<br>RRID:AB_10846953
BBS4	Proteintech	12766-1-AP<br>RRID:AB_10596774
ARL13B	Proteintech	17711-1-AP;<br>RRID:AB_2060867
STX3	Proteintech	15556-1-AP;<br>RRID:AB_2198667
SLC1A1/EAAC1	Proteintech	12686-1-AP;<br>RRID:AB_2188001
PRKCA	AVIVA	OAAN00067
Phospho-PRKCA	AVIVA	OAAJ02486

TOR1A	ThermoFisher	PA5-65651; RRID:AB_2662036
PMCA	EMDMillipore	MABN1802
mCherry	Rockland	200-301-379; RRID:AB_2611063
TFG	Proteintech	11571-1-AP; RRID:AB_2203102
SV2	DSHB	SV2; RRID:AB-2315387
Goat Anti-Mouse secondary antibody, HRP	Thermofisher	31432; RRID:AB_228302
Goat Anti-Rabbit secondary antibody, HRP	Thermofisher	G-21234; RRID:AB_1500696
Donkey Anti-Goat secondary antibody, HRP	Thermofisher	PA1-28664; RRID:AB_10990162
AlexaFluor A488, Goat Anti-Rabbit secondary antibody	Thermofisher	A11034; RRID:AB_2576217
AlexaFluor A488, Goat Anti-Mouse secondary antibody	Thermofisher	A11029; RRID:AB_2534088
AlexaFluor A568, Goat Anti-Rabbit secondary antibody	Thermofisher	A11036; RRID:AB_10563566
AlexaFluor A568, Donkey Anti-Goat secondary antibody	Thermofisher	A11057; RRID:AB_2534104

Hoechst 33342	BD Biosciences	561908; RRID:AB_2869394
**Oligonucleotides**		
PERK-forward:<br>CAC AGG GAC CTC AAG<br>CCT TCC	This paper	N/A
PERK-reverse:<br>GGA CTC GTT CCA TCT<br>GGG TGC	This paper	N/A
IRE1α-forward:<br>GAC GAC<br>GTGGACTACAAGATG	This paper	N/A
IRE1α-reverse:<br>GGC GTT AGC TTG CTC<br>TTG GCC	This paper	N/A
ATF6-forward:<br>GGG TTC GGA TAT CGC<br>TGT GCT G	This paper	N/A
ATF6-reverse:<br>GCT CAT GGG CCC ATA<br>GTT CAG C	This paper	N/A
**DNA plasmids**		
His-CFAP418	(2)	N/A

GST-CFAP418 FL	(2)	N/A
GST-CFAP418 CT	(2)	N/A
FLAG-CFAP418	(2)	N/A
EGFP-CFAP418	(2)	N/A
mCherry-RAB28	(3)	N/A
mCherry-RAB28 T26N	Guoxin Ying, University of Utah, Salt Lake Ciity, Utah, USA	N/A
mCherry-RAB28 Q72L	This paper	N/A

METHOD DETAILS

<u>Pulse-chase experiment</u>: Pulse-chase experiments were conducted at 37°C in a humidified 5% CO_2 incubator. Four sets of three retinas per genotype were incubated in DMEM without methionine or cysteine (ThermoFisher Scientific. cat# 21013) but supplemented with 200 mM cysteine (SigmaAldrich C7352) for 30 minutes. The medium was then changed to DMEM with cysteine and 0.05 µCi/µl ^{35}S methionine (PerkinElmer, cat. NEG009A). Pulse labeling was done for 30, 60, 90, or 120 minutes. For the chase experiment, the radioactive medium was replaced with cold DMEM, and incubation was carried out for 3 hours or overnight. The radiolabeled retinas were stored at -80°C. The frozen retinas were subsequently homogenized by repetitive pipetting on ice in PBS with 0.5% TritonX-100 and cOmplete protease inhibitors (Roche). The homogenates were precleared by incubation with protein G Sepharose (GE Healthcare, cat#17-0618-02) for 1 hour at 4°C. After centrifugation, the supernatants were incubated overnight at 4°C with Protein G-bound antibodies, which were generated by mixing antibodies with Protein G

in phosphate-buffered saline (PBS) and 1% TritonX-100 for 2 hours at 4°C. Following the incubation, samples were washed three times with wash buffer (50 mM Tris pH7.5, 0.3 M NaCl, 0.1% TritonX-100, 5 mM EDTA) and once with PBS. The final pellets were extracted by adding protein gel loading buffer, run on a 10% SDS-polyacrylamide gel, dried, exposed to a storage phosphor screen, and scanned by an S35 Typhoon Trio Phosphoimager (GE Healthcare).

RT-qPCR: RT-qPCR was conducted as previously described (2). Briefly, retinal total RNA was isolated using the SurePrep RNA purification kit (Fisher Scientific, Hampton, NH) and reverse transcribed using the ThermoScript RT-PCR system (ThermoFisher Scientific, Waltham, MA). qPCR was performed using the SYBR Premix Ex Taq kit (TaKaRa Bio USA, San Jose, CA) and the CFX Connect real-time PCR machine (Bio-Rad, Hercules, CA). The primer information is shown in the Key Resources Table. The PCR condition was 95°C for 2 minutes followed by 45 cycles of 95°C for 10 sec and 55°C for 30 sec (60°C for IRE1α). *C*q was determined using the single threshold mode of the CFX Manager software.

Lipid binding experiment: His- and GST-tagged CFAP418 proteins were expressed in One Shot™ BL21 Star™ (DE3) cells (ThermoFisher, Waltham, MA) and purified using HisPur™ Ni-NTA resin and Pierce™ Glutathione Superflow Agarose, respectively (ThermoFisher, Waltham, MA). After spotting 1 ng of the purified recombinant proteins on the membrane lipid and PIP strips (P-6002 and P-6001, respectively, Echelon Biosciences, Salt Lake City, UT) as positive controls, the strips were blocked in 3% bovine serum albumin in PBS-T buffer for 1 hour and then incubated in the same buffer with the recombinant proteins at a concentration of 1 μg/ml. The bound CFAP418 protein was detected by sequential incubations with CFAP418 antibody (2) and a horseradish peroxidase-conjugated secondary antibody. The enhanced chemiluminescence

was scanned by a FluorChem Q machine (Cell Biosciences, Inc., Santa Clara, CA) or an iBright™ CL750 imaging system (ThermoFisher, Waltham, MA).

Transmission electron microscopy: Mouse eyeballs were enucleated and briefly fixed in 1% formaldehyde and 2.5% glutaraldehyde in 0.1 M cacodylate buffer, pH 7.5. The cornea and lens were then removed, and the obtained eye cups were continued to be fixed overnight. The subsequent post-fixation with 2% osmium tetroxide, dehydration with a graded alcohol series, embedding in Epon, sectioning with ultramicrotome, and post-staining with lead citrate were performed as described previously (2). The transmission electron micrographs were taken using a JEOL JEM-1400 TEM equipped with a Gatan 4000 camera or a Gatan Orius SC1000B camera. Measurement was conducted in the longitudinal view of photoreceptors using Viking (https://connectomes.utah.edu). Mitochondrial diameter was calculated by averaging the thickness measurements from the top, middle, and bottom of the same mitochondria. The mitochondrial diameter variation was calculated as the difference between the smallest and largest thickness of the same mitochondria.

Oxygen consumption rate measurement: Mouse retinas were dissected free of RPE and flattened. Two 1-mm punches were collected surrounding the optic nerve head per retina using a biopsy puncher (Integra Miltex, Mansfield, MA). Each punch was placed with ganglion cell layer facing up in 180 µl medium (DMEM, 100 mM glucose, 2 mM glutamine, and 1 mM sodium pyruvate) in each well of the Seahorse Xfe96 Spheroid Microplate (102959-100). The OCR was measured using the Seahorse XF Cell Mito Stress test kit according to the manufacturer's protocol. After the samples were incubated at 37 degrees for about 20 minutes, basal respiration was measured for 6 cycles. Then, 8, 3, and 5 cycles were measured after

oligomycin (2 μm, 20 μl), FCCP (0.5 μm, 22 μl), and rotenone/antimycin A (2 μm, 24 μl) were injected, respectively. Medium without retinal punches was included as negative controls. The average of four retinal punches from one mouse was considered as an independent data point. Data was analyzed and presented using Wave 2.6.1 and Prism GraphPad 10.0.3.

Immunoblotting, immunoprecipitation, and immunostaining: Immunoblotting, immunoprecipitation, and immunostaining procedures were described previously with minor changes (4). Briefly, mouse retinas were homogenized by pipetting up and down using a wide-opening pipet tip and lysed at 4°C for 30 minutes in a solubilization buffer (50 mM Tris-HCl, pH 7.5, 100 mM NaCl, 5 mM EDTA, 1% Triton X-100, 0.05% SDS, 2.5% glycerol, and 1.0 mM phenylmethylsulphonyl fluoride). The retinal lysates were centrifuged at 14,000 x g for 12 minutes. The SDS sample loading buffer was added to the supernatants, which were run on a 10% or 12% SDS-PAGE. To separate retinal cytosol and membrane fractions, the retinal lysate, cleared by centrifugation at 14,000 x g for 10 minutes, was ultracentrifuged at 100,000 x g for 30 minutes. The membrane proteins were extracted from the pellet using lysis buffer containing 1% Triton X-100. Alternatively, the retinal cytosol and membrane proteins were separated using the Mem-PER™Plus membrane protein extraction kit (Cat #: 89842, ThermoFisher Scientific, Waltham, MA). The SDS sample loading buffer was added to the cytosol and membrane fractions, which were run on a 10% SDS-PAGE. The immunoblot signals were developed using ProSignal® Pico ECL Reagent (Genesee Scientific, San Diego, CA), scanned by a FluorChem Q machine (Cell Biosciences, Inc., Santa Clara, CA) or an Invitrogen™ iBright™ CL750 machine (ThermoFisher Scientific, Waltham, MA), and quantified using ImageJ. The intensity of the immunoblot signals was normalized by loading control γ-tubulin signals in

the same lanes. The immunoprecipitation procedure was described in the affinity purification section. For cultured cells, immunoprecipitation was performed at 24 hours post-transfection.

For immunostaining, mouse eyeballs were enucleated and fixed briefly in 4% formaldehyde in PBS. After removal of the cornea and lens, the eyecups were continued to be fixed for 1 hour before cryosection at 12 μm using Leica CM3050S cryostat. COS-7 and HEK293 cells were transfected with DNA plasmids using PEI (Polysciences, Inc., Warrington, PA). At 24 hours post-transfection, cells were fixed by methanol:acetone (19:1) at -20°C for 10 minutes. The fixed retinal sections and transfected cells were then subjected to standard immunostaining procedures, i.e., primary antibody incubation at 4ºC overnight and secondary antibody incubation at room temperature for 1 hour. The immunofluorescence signals were captured using a Leica SP8 confocal microscope with a HC PL APO 63X1.40 OIL CS2 objective. The colocalization of proteins in cultured cells was analyzed using the Coloc module of Imaris 9.7.2 and 9.8.2 (Oxford Instruments, Concord, MA). To quantify the vacuole accumulation phenotype, transfections of COS-7 cells with GFP-CFAP418 and GFP were conducted four times. Cells with vacuole accumulation were counted from 135 to 360 transfected cells in each experiment by a person blind to treatment. The dilution ratios of primary and secondary antibodies for immunoblotting, immunoprecipitation, and immunostaining were based on manufacturers' suggestions. The source of the antibodies can be found in the Key Resource Table.

Label-free quantitative MS: This experiment was conducted by the Mass Spectrometry Proteomics Core at Baylor College of Medicine.

MS analysis: The retinal sample preparation and high pH C18 reverse phase sample preparation were performed as described previously (5). Briefly, the tissue lysate was digested using trypsin enzyme followed by an offline high pH C18 reverse phase fractionation. The peptides were eluted in a stepwise gradient of acetonitrile (2%, 4%, 6%, 8%, 10%, 12%, 14%, 16%, 18%, 20%, 22%, 24%, 26%, 28% and 30%) and pooled into five fractions (2+12+22; 4+14+24; 6+16+26; 8+18+28; 10+20+30). These fractions were then subjected to the second dimensional low pH reversed-phase chromatography. The fractionated peptides were loaded onto a Reprosil-Pur Basic C18 (1.9 µm, Dr.Maisch GmbH, Germany) precolumn of 2 cm X 100 µm size. The precolumn was switched in-line with an in-housed 50 mm x 150 µm analytical column packed with Reprosil-Pur Basic C18 equilibrated in 0.1% formic acid/water. The peptides were analyzed using a nano-LC 1200 system (Thermo Fisher Scientific, San Jose, CA) coupled to Orbitrap Fusion™ Lumos ETD (Thermo Fisher Scientific, San Jose, CA) mass spectrometer operated in the data-dependent acquisition mode acquiring fragmentation spectra of the top 30 strongest ions and under the direct control of Xcalibur software (Thermo Scientific).

Protein identification and quantification: Raw files from Orbitrap Fusion Lumos were searched with Mascot algorithm (Mascot 2.4, Matrix Science) with percolator against the mouse NCBI refseq protein database (updated on 2019/01/14) in the Proteome Discoverer

(PD2.1, Thermo Fisher). Dynamic modification of Oxidation, protein N-terminal Acetylation, Phosphorylation on serine, tyrosine and threonine, Deamidation on asparagine and glutamine was allowed. The precursor mass tolerance was confined within 20 ppm with fragment mass tolerance of 0.5 dalton and a maximum of two missed cleavage was allowed. The Peptide Spectrum Matches (PSMs) output from PD2.1 was grouped into gene products by 'gpGrouper' algorithm (6) and quantification was done using the label-free, intensity-based absolute quantification (iBAQ) approach and then normalized to FOT (a fraction of the total protein iBAQ amount per experiment). FOT was defined as individual protein's iBAQ divided by the total iBAQ of all identified proteins within one experiment.

TMT-labeling quantitative MS: This experiment was conducted by the Thermo Fisher Center for Multiplexed Proteomics at the Harvard Medical School.

Preparation of samples for peptide isobaric labeling and MS: Retinal tissues were prepared for quantitative analysis, as previously described with some modifications (7). Tissue was lysed in 8M urea, 200 mM EPPS pH 8.0 with EDTA free cOmplete protease inhibitor cocktail and PhosSTOP phosphatase inhibitor (Roche). Total protein was quantified by micro-BCA assay (Pierce). Proteins were reduced with 5 mM TCEP and alkylated with 15 mM iodoacetamide. Total protein was precipitated with 20% trichloroacetic acid on ice, pelleted at 21,000 g, and washed twice with cold acetone. Proteins were resuspended in 200 mM EPPS, pH 8.0 and digested overnight with LysC (Wako, Japan) in a 1:50 enzyme:protein ratio followed by trypsin for 6 hours at 37°C (1:100 enzyme:protein ratio). Peptides were quantified by micro-

BCA assay and 75 μg of peptide per sample were labeled with TMT10 reagents (Thermo-Fisher) for 2 hours at room temperature. Labeling reactions were quenched with 0.5% hydroxylamine and acidified with trifluoroacetic acid. Acidified peptides were combined and desalted by Sep-Pak (Waters).

Basic pH reversed-phase separation: TMT labeled peptides were solubilized in 5% acetonitrile (ACN)/10 mM ammonium bicarbonate (pH 8.0), and 300 μg of TMT labeled peptides was separated by an Agilent 300 Extend C18 column (3.5 μm particles, 4.6 mm ID and 250 mm in length). An Agilent 1260 binary pump coupled with a photodiode array detector (Thermo Scientific) was used to separate the peptides. A 45-minute linear gradient from 10% to 40% ACN in 10 mM ammonium bicarbonate pH 8.0 (flow rate of 0.6 mL/min) separated the peptide mixtures into a total of 96 fractions (36 seconds). A total of 96 fractions were consolidated into 24 samples in a checkerboard fashion, acidified with 20 μL of 10% formic acid (FA), and vacuum dried to completion. Each sample was desalted via Stage Tips and re-dissolved in 5% FA/ 5% ACN for LC-MS3 analysis.

Liquid chromatography separation and tandem mass spectrometry (LC-MS3): Data were collected using an Orbitrap Fusion mass spectrometer (Thermo Fisher Scientific) coupled with a Proxeon EASY-nLC 1200 LC pump (Thermo Fisher Scientific). Peptides were separated on a 75-μm inner diameter microcapillary column packed with 35 cm of GP-18 resin (2.6 μm, 200 Å, Sepax, Newark, DE). For each sample, ~1.5 μg of peptides were

separated using a 3-hour gradient of 7–32% ACN in 0.125% FA with a flow rate of 500 nL/min.

Each analysis used an MS^3-based TMT method with real-time spectral searching as described previously (8). MS1 scans were acquired in the Orbitrap using a mass range of *m/z* 400 – 1500, resolution 120,000, AGC target 2.5 x 10^6, maximum injection time 50 ms, and dynamic exclusion of 180 seconds. Data dependent top 10 MS^2 spectra were acquired in the ion trap with a normalized collision energy set at 35%, AGC target set to 2.5 x 10^4 and a maximum injection time of 35 ms. MS2 spectra were searched in real time using a modified version of COMET against a mouse reference protein database (Uniprot) and common contaminants were filtered using a real-time FDR filter. Peptide spectral matches passing FDR filter and not matching to decoy peptides trigger SPS-MS3 scans collected in the Orbitrap.

Data analysis: A compendium of in-house developed software was used to convert mass spectrometric data (.RAW), file processing, monoisotopic peak correction, controlling peptide and protein level FDRs, assembling proteins from peptides, and protein quantification from peptides (9, 10). MS2 spectra were searched with COMET (11) against the mouse UniProt database (downloaded July 2014) appended with reversed protein sequences and known contaminants. COMET search parameters were 50 ppm precursor ion tolerance, 1.0005 fragment ion tolerance, fully tryptic with a maximum of 2 missed cleavage sites, Static modification of N termini and lysine residues by Ten-plex TMT tags (+ 229.162932 Da), static carboxyamidomethylation of cysteine residues (+57.02146 Da), and

differential oxidation of methionine residues (+ 15.99492 Da). Peptide and protein were filtered to an FDR of less than 1% by applying the target-decoy database search and linear discriminant analysis (9, 10).

Proteins were quantified as previously described (12). A 0.003 *m/z* window centered on the theoretical m/z value of each of the six reporter ions and the intensity of the signal closest to the theoretical m/z value was recorded. Reporter ion intensities were adjusted based on the overlap of isotopic envelopes of all reporter ions (as determined by the manufacturer). Only peptides with a total summed reporter ion signal to noise greater than 100 and MS2 isolation specificity greater than 0.5 were used for protein quantification.

Lipidomic MS: This experiment was conducted by the Metabolomics Core at the University of Utah.

Chemicals: LC-MS-grade solvents and mobile phase modifiers were obtained from Honeywell Burdick & Jackson, Morristown, NJ (ACN, isopropanol, FA), Fisher Scientific, Waltham, MA (methyl *tert*-butyl ether) and Sigma–Aldrich/Fluka, St. Louis, MO (ammonium formate).

Sample preparation: Extraction of lipids was carried out using a biphasic solvent system of cold methanol, methyl *tert*-butyl ether (MTBE), and water with some modifications (13). In a randomized sequence, tissue lipids were extracted in bead-mill tubes (ceramic 1.4 mm, Mo-Bio, Qiagen, Germantown, MD) containing a solution of 225 µL methanol, 750 µL MTBE,

and internal standards (Lipid standard Mouse SPLASH LipidoMix at 10 μL per sample, Avanti Polar Lipids, Alabaster, AL). Samples were homogenized in one 30-second cycle and rested on ice for 1 hour with occasional vortexing. Then, 188 μL of PBS was added followed by a brief vortex. Samples were centrifuged at 14,000 x *g* for 10 minutes at 4 °C, and the upper phases were collected. Another aliquot of 750 μL MTBE was added to the bottom aqueous layer followed by a brief vortex. Samples were then centrifuged at 14,000 x *g* for 10 minutes at 4 °C, the upper phases were combined and evaporated to dryness under speedvac. Lipid extracts were reconstituted in 250 μL of mobile phase B and transferred to an LC-MS vial for analysis. Concurrently, a process blank sample was prepared and then a pooled quality control (QC) sample was prepared by taking equal volumes (~50 μL) from each sample after final resuspension.

<u>LC-MS analysis:</u> Lipid extracts were separated on an Acquity UPLC CSH C18 column (2.1 x 100 mm; 1.7 μm) coupled to an Acquity UPLC CSH C18 VanGuard precolumn (5 × 2.1 mm; 1.7 μm) (Waters, Milford, MA) maintained at 65 °C connected to an Agilent HiP 1290 Sampler, Agilent 1290 Infinity pump, and Agilent 6545 Accurate Mass Q-TOF dual AJS-ESI mass spectrometer (Agilent Technologies, Santa Clara, CA). Samples were analyzed in a randomized order in both positive and negative ionization modes in separate experiments acquiring with the scan range m/z 100 – 1700. For positive mode, the source gas temperature was set to 225 °C, with a drying gas flow of 11 L/minute, nebulizer pressure of 40 psig, sheath gas temp of 350 °C and sheath gas flow of 11 L/minute. VCap voltage is set at 3500 V, nozzle

voltage 500V, fragmentor at 110 V, skimmer at 85 V and octopole RF peak at 750 V. For negative mode, the source gas temperature was set to 300 °C, with a drying gas flow of 11 L/minute, a nebulizer pressure of 30 psig, sheath gas temp of 350 °C and sheath gas flow 11 L/minute. VCap voltage was set at 3500 V, nozzle voltage 75 V, fragmentor at 175 V, skimmer at 75 V and octopole RF peak at 750 V. Mobile phase A consisted of ACN:H_2O (60:40 *v/v*) in 10 mM ammonium formate and 0.1% FA, and mobile phase B consisted of IPA:ACN:H_2O (90:9:1 *v/v/v*) in 10 mM ammonium formate and 0.1% FA. The chromatography gradient for both positive and negative modes started at 15% mobile phase B then increased to 30% B over 2.4 min. It sequentially increased to 48% B from 2.4 – 3.0 min, 82% B from 3 – 13.2 min, and 99% B from 13.2 – 13.8 min where it's held until 16.7 min and returned to the initial conditions and equilibrated for 5 min. Flow was 0.4 mL/minute throughout, with injection volumes of 2 μL for positive and 10 μL for negative mode, and iterative, tandem mass spectrometry was conducted using the same LC gradient at collision energies of 20 V and 27.5 V, respectively.

<u>LC-MS data processing:</u> For data processing, Agilent MassHunter (MH) Workstation and software packages MH Qualitiative and MH Quantitative were used. The pooled QC (n=8) and process blank (n=4) were injected throughout the sample queue to ensure the reliability of acquired lipidomics data. For lipid annotation, accurate mass and MS/MS matching was used with the Agilent Lipid Annotator library. Results from the positive and negative ionization modes from Lipid Annotator were merged based on the class of lipid identified.

Data exported from MH Quantitative was evaluated using Excel where initial lipid targets were parsed based on the following criteria. Only lipids with relative standard deviations less than 30% in QC samples were used for data analysis. Additionally, only lipids with background AUC counts in process blanks that were less than 30% of QC were used for data analysis. The parsed excel data tables were normalized based on the ratio to class-specific internal standards, then to sum prior to statistical analysis.

Affinity-purification coupled with mass spectrometry:

Affinity purification: Mouse and bovine retinas were homogenized and incubated in lysis buffer (200 mM NaCl, 20 mM Tris, 0.5% Triton X-100, 1 mM TCEP, protease inhibitor cocktail, pH8.0) for 30 minutes at 4°C. The homogenates were then centrifuged at 100,000 x g at 4°C for 30 minutes. For mouse retinas, the affinity purification was conducted by incubating the obtained supernatants with our custom-made CFAP418 antibody and subsequently protein G sepharose (Fisher scientific, Waltham, MA) for 30 minutes each or with the CFAP418 antibody cross-linked with protein G for 30 minutes total. The protein G beads were spun down at 3,500 x g for 30 seconds and washed four times with the lysis buffer. For bovine retinas, the supernatants were incubated with recombinant GST-tagged CFAP418 proteins expressed in One Shot™ BL21 Star™ (DE3) cells (ThermoFisher, Waltham, MA) and purified using Pierce™ Glutathione Superflow Agarose (ThermoFisher, Waltham, MA). The precipitated proteins were eluted from the protein G sepharose or Glutathione agarose beads using 2X SDS sample loading buffer and run on a 10% SDS-PAGE. Each protein lane was cut into 2-4 sections and submitted to the Taplin Mass Spectrometry Facility at Harvard Medical School for protein identification. More details are shown in Supplemental Table 6.

Gel band processing: Gel bands were cut into approximately 1-mm^3 pieces and subjected to a modified in-gel trypsin digestion procedure (14). Briefly, gel pieces were washed and dehydrated with ACN for 10 minutes. After ACN removal, pieces were completely dried in a speed-vac and rehydrated at 4ºC for 45 minutes with 50 mM ammonium bicarbonate solution containing 12.5 ng/µl modified sequencing-grade trypsin (Promega, Madison, WI). After removal of excess trypsin, replacement with 50 mM ammonium bicarbonate solution, and incubation at 37ºC overnight, peptides were extracted by removing the ammonium bicarbonate solution, one wash with a solution containing 50% ACN and 1% FA, and dried in a speed-vac for ~1 hour.

LC-MS/MS analysis: Gel extraction samples were reconstituted in 5 - 10 µl of HPLC solvent A (2.5% ACN and 0.1% FA). A reverse-phase HPLC capillary column was generated by packing 2.6 µm-C18 spherical silica beads into a fused silica capillary (100 µm inner diameter x ~30 cm length) (15). After equilibrating the column, samples were loaded through a Famos auto sampler (LC Packings, San Francisco CA). Peptides were eluted with increasing concentrations of solvent B (97.5% ACN and 0.1% FA). As peptides were eluted, they were subjected to electrospray ionization and entered into an LTQ Orbitrap Velos Pro ion-trap mass spectrometer (ThermoFisher Scientific, Waltham, MA). Peptides were detected, isolated, and fragmented to produce a tandem mass spectrum of specific fragment ions. Peptide sequences and protein identity were determined by matching protein databases with the acquired fragmentation pattern by Sequest (ThermoFisher Scientific, Waltham, MA) (16). All databases included a reversed version of all the sequences and the data were filtered between a 1 and 2% peptide FDR.

Quantitative proteomic, phosphoproteomic, lipidomic analyses and affinity purification-mass spectrometry: Proteins detected by label-free or TMT-labeling quantitative MS were ranked based on their values of -log(*p* value)*sign(log(FC)). For each protein, the *p* value was calculated

using the normalized MS values generated by MetaboAnalyst 5.0 (17). The normalization parameters in MetaboAnalyst were set as normalization to constant sum; log10 transformation, and mean centering. The ranked proteins were then analyzed using the Gene Set Enrichment Analysis software (GSEA desktop v4.1.0) and the gene set databases of c2.cp.reactome.v7.4.symbols.gmt and c5.go.v7.4.symbols.gmt. The Run GSEAPreranked tool with default basic and advanced parameters was applied except that the gene set size was set to 15-200 and the mouse gene symbols were remapped to human orthologs. At P5, 21 and 1 out of the 4,033 screened gene sets were down-regulated and up-regulated in *Cfap418*$^{-/-}$ retinas, respectively (FDR $q < 0.05$, Supplemental Table 4). At P10, 24 and 86 out of the 3988 screened gene sets were down-regulated and up-regulated in *Cfap418*$^{-/-}$ retinas, respectively (FDR $q < 0.05$, Supplemental Table 4). The up- and down-regulated gene sets were visualized and further analyzed by EnrichmentMap and AutoAnnotate using Cytoscape 3.8.1 (18). Proteins identified multiple times in one proteomic experiment were counted as one entry in the Venn Diagram analyses. The Venn Diagrams of the quantitative proteomic and phosphoproteomic MS data were drawn using VENNY 2.1 (https://bioinfogp.cnb.csic.es/tools/venny/). The Venn Diagrams of the AP-MS data were drawn using InteractiVenn (19).

Statistics: Protein and phosphoprotein levels generated from label-free and TMT-labeling MS were processed using the Statistical Analysis module in MetaboAnalyst 5.0 (17, 20). The wild-type and *Cfap418*$^{-/-}$ samples were normalized by their own sum, converted to an approximately normal distribution by logarithmic transformation (base 10), and scaled by mean centering. Principal component analysis (PCA) and heatmap hierarchical clustering with default parameters were implemented to examine the data quality and detect the

potential outliers. The t-test in the Univariate Analysis category was conducted to identify differentially expressed (DE) and phosphorylated (DP) proteins. Because very few proteins had an FDR value (adjusted *p* value for multiple comparisons) smaller than 0.05, we defined DE and DP proteins as long as their raw *p* values were smaller than 0.05. There was no fold change cutoff for DE proteins at P5 or DP proteins at P5 or P10, while the fold change cutoff was 1.1 for the DE proteins at P10. The fold change was calculated by the ratio of *Cfap418*$^{-/-}$ protein/phosphoprotein level to wild-type protein/phosphoprotein level.

Lipidomic data were also processed and analyzed in MetaboAnalyst 5.0 (17, 20). The *Cfap418*$^{+/-}$ and *Cfap418*$^{-/-}$ samples were normalized by their own sum and scaled by mean centering without any data transformation. PCA and heatmap hierarchical clustering detected sample 14 in the *Cfap418*$^{-/-}$ group as an outlier (Supplemental Figure 11B and not shown). This sample was excluded from downstream analyses. Differentially abundant lipid species were identified by t-test with an FDR value smaller than 0.05 and a ratio of *Cfap418*$^{-/-}$ to *Cfap418*$^{+/-}$ value larger than 1.05 or smaller than 0.95. Lipid enrichment analysis was implemented using the Quantitative Enrichment Analysis (QEA) algorithm offered by the Metabolite Set Enrichment Analysis (MSEA) module. The metabolite set library of 1072 sub-chemical class metabolite sets was selected.

The results of RT-qPCR and semi-quantitative immunoblot analyses, the abundances of aggregated lipid categories and acyl chains, and mitochondrial diameter and diameter variation were compared between *Cfap418*$^{-/-}$ and control (*Cfap418*$^{+/+}$ or *Cfap418*$^{+/-}$) groups using Student's t-test in Microsoft Excel.

Supplementary Information

Supplemental Table 1: Original data from quantitative proteomic, phosphoproteomic, and lipidomic studies. Sheet 1: Proteomic data at P5. Sheet 2: Phosphoproteomic data at P5. Sheet 3: Proteomic data at P10. Sheet 4: Phosphoproteomic data at P10. Sheet 5: lipidomic data at P10.

Supplemental Table 2: DE proteins, DP proteins, and altered lipid species identified in *Cfap418*$^{-/-}$ retinas by MetaboAnalyst. Reduced DE and DP proteins and lipids are highlighted in blue. Increased DE and DP proteins and lipids are highlighted in yellow. Other proteins and lipids have a p-value or an adjusted p-value between 0.05 and 0.1. Sheet 1: Proteomic data at P5. Sheet 2: Phosphoproteomic data at P5. Sheet 3: Proteomic data at P10. Sheet 4: Phosphoproteomic data at P10. Sheet 5: lipidomic data at P10.

Supplemental Table 3: DE and DP proteins identified in *Cfap418*$^{-/-}$ retinas at both P5 and P10. Sheet 1: the proteomic and phosphoproteomic data. Sheet 2: Known information and references of the shared DE proteins.

Supplemental Table 4: GSEA reports of biological pathways, molecular functions, and cellular compartments that are affected by *Cfap418* knockout at P5 (sheet 1: down-regulated; sheet 2: up-regulated) **and P10** (sheet 3: down-regulated; sheet 4: up-regulated)**.**

Supplemental Table 5: Proteins immunoprecipitated by CFAP418 antibody from mouse retinas and proteins pulled down by GST-CFAP418 baits from bovine retinas.

Supplemental Table 6: AP-MS experimental design, condition, and result.

Bait	Retina #	Control	Candidate #
GST-CFAP418 FL (50 μg)	3 (bovine)	GST (120 μg)	94
GST-CFAP418 CT (100 μg)	3 (bovine)	GST (120 μg)	116
CFAP418 antibody (50 μg) cross-linked with protein G	28 (*Cfap418*$^{+/-}$)	20 *Cfap418*$^{-/-}$ retinas	508
CFAP418 antibody (50 μg)	9 (*Cfap418*$^{+/-}$)	18 *Cfap418*$^{-/-}$ retinas &<br>9 *Cfap418*$^{+/-}$ retinas with IgG (50 μg)	208
CFAP418 antibody (50 μg)	11 (*Cfap418*$^{+/-}$)	22 *Cfap418*$^{-/-}$ retinas<br>11 *Cfap418*$^{+/-}$ retinas with IgG (50 μg)	264
CFAP418 antibody (50 μg)	40 (*Cfap418*$^{+/-}$)	40 *Cfap418*$^{-/-}$ retinas	261
CFAP418 antibody (50 μg)	40 (*Cfap418*$^{+/-}$)	40 *Cfap418*$^{-/-}$ retinas	258

Supplemental Figure 1: CFAP418 protein sequence alignment across different species. Red, blue, and black residues represent highly, mildly, and not conserved residues, respectively. Patient mutations are shown on the top of sequences. Splice site, nonsense, and frameshift mutations are denoted by vertical lines, and missense mutations are highlighted in grey.

Supplemental Figure 2: GSEA reveals a reduction of photoreceptor proteins and pathways in *Cfap418*$^{-/-}$ retinas. Photoreceptor IS and phototransduction proteins are down-regulated in P5 *Cfap418*$^{-/-}$ retinas. Ciliary membrane proteins including photoreceptor OS membrane proteins are down-regulated in P10 *Cfap418*$^{-/-}$ retinas.

Supplemental Figure 3: Vacuole accumulation in CFAP418-transfected cells. (**A**) Representative COS-7 cells transfected with FLAG- and GFP-CFAP418, but not GFP, accumulate large vacuoles (filled arrows). Open arrows indicate thin edges of vacuoles. n: nucleus. Scale bars: 10 μm. (**B**) Quantification of the vacuole accumulation phenotype in GFP-CFAP418- and GFP-transfected COS-7 cells. Dot plot represents data from individual experiments and mean ± SEM. **, $p < 0.01$ (Student's t-test).

Supplemental Figure 4: Colocalization analysis of CFAP418 with ESCRT and RAB28 proteins. (**A**) Representative COS-7 cells transfected with FLAG-CFAP418 and double-immunostained for FLAG and ESCRT proteins. (**B**) Representative COS-7 cells double-transfected with FLAG-CFAP418 and mCherry-RAB28 mutant proteins. Cells were immunostained using FLAG and mCherry antibodies. Arrows point to the abnormally accumulated vacuoles. Framed regions are amplified and shown on the right with merged and individual channels. The PCCs are shown as mean ± SEM. n, number of cells analyzed. Scale bars: 10 μm.

Supplemental Figure 5: Abnormal HGS and STAM but normal RAB28 distributions in P10 *Cfap418$^{-/-}$* photoreceptors. (**A**) HGS and (**B**) STAM, but not RAB28, signal patterns are different in P10 *Cfap418$^{+/-}$* and *Cfap418$^{-/-}$* photoreceptors. White lines outline the RPE and OS border and the IS and ONL border, based on the corresponding bright field channels. The zoomed images on the right are independent images from the ones on the left. (**C**) Double immunostaining for RAB28 and rhodopsin in P10 *Cfap418$^{+/-}$* and *Cfap418$^{-/-}$* photoreceptors. As reported previously (3), RAB28 is in photoreceptor OS, IS, and OPL. The strongest RAB28 signal is present in the OS, marked by rhodopsin. Scale bars: 2 μm (zoomed) and 10 μm (others).

Supplemental Figure 6: Localization of vesicular trafficking proteins in *Cfap418$^{-/-}$* photoreceptors. (**A**) TFG and VPS4B proteins are localized normally in *Cfap418$^{-/-}$* photoreceptors, compared with littermate *Cfap418$^{+/-}$* photoreceptors at P10. (**B**) Early endosomal proteins EEA1 and RAB5, late endosomal protein RAB7, and recycling endosomal protein RAB11 appear to be distributed normally in *Cfap418$^{-/-}$* photoreceptors, compared with littermate *Cfap418$^{+/-}$* photoreceptors at P21. Scale bars: 10 μm.

Supplemental Figure 7: Abnormal mitochondrial protein expression and morphology but normal mitochondrial respiration in *Cfap418$^{-/-}$* retinas. (**A**) GSEA shows mitochondrial translation proteins are enriched in the down-regulated proteins in P10 *Cfap418$^{-/-}$* retinas. (**B**) A longitudinal view of *Cfap418$^{-/-}$* photoreceptors shows uneven diameters along the length of mitochondria at P21 and P60, compared with the smooth straight long bar-shaped mitochondria in *Cfap418$^{+/-}$* littermate photoreceptors. Red arrows point to the abnormal constrictions and protruding bumps of the mitochondria. Scale bars, 1.5 μm. (**C**) OCR is comparable between *Cfap418$^{+/+}$* and *Cfap418$^{-/-}$* littermate retinas at 3-5 weeks of age. Each data point is the mean ±

SEM from 10 mice, and the datum of each mouse is the mean of 4 retinal punches from two eyes. Repeated measures two-way ANOVA was conducted with genotype as a between-subject variable and time as a within-subject variable.

Supplemental Figure 8: Symporter expression is increased in P10 *Cfap418-/-* retinas. (**A**) GSEA identified increased symporter activity in P10 *Cfap418*$^{-/-}$ retinas. (**B**) The expressions of leading symporters, revealed by GSEA, in *Cfap418*$^{+/+}$ and *Cfap418*$^{-/-}$ retinas at P5 and P10. Legends are the same as those in Figure 2B. (**C**) SLC1A1 is present in all photoreceptor layers at P10. SV2 labels the photoreceptor OPL. There is no significant difference in SLC1A1 distribution between P10 *Cfap418*$^{+/-}$ and *Cfap418*$^{-/-}$ photoreceptors. (**D**) SLC1A1 is present in OS apex, ONL, and OPL in P21 photoreceptors. OPL is marked by PMCA. Note that there is a tissue separation between OS and RPE in these images, as shown in the bright field (BF) channel. Scale bars, 10 μm.

Supplemental Figure 9: Evaluation of OS membrane protein synthesis, degradation, and ER stress in *Cfap418*$^{-/-}$ retinas. (**A**) Pulse labeling using [^{35}S] methionine for up to 2 hours shows no obvious reduction in newly synthesized rhodopsin (RHO) in *Cfap418*$^{-/-}$ retinas at P12, compared with *Cfap418*$^{+/-}$ littermate retinas. DPYSL2 is a cytoplasmic protein and was used as a negative control. Left, SDS-PAGE autoradiograms of pulse-labeled RHO and DPYSL2 proteins immunoprecipitated from retinal explants. Right, quantification of the RHO and DPYSL2 radioactivity from the autoradiograms. (**B**) Chase labeling of *Cfap418*$^{+/-}$ and *Cfap418*$^{-/-}$ littermate retinal explants for 3 hours at P12 and overnight at P8 using [^{35}S] methionine shows no obvious changes in the degradation of RHO or transducin α subunit (GNAT1). Left, representative SDS-PAGE autoradiograms of chase-labeled RHO and GNAT1 proteins immunoprecipitated from

retinal explants. Right, quantitation of radioactive RHO and GNAT1 signals. (**C**) RT-qPCR analysis shows no changes in the PERK, IRE1α, and ATF6 mRNAs in *Cfap418*$^{-/-}$ retinas at P15 and P30. Each dot represents the retinas of an individual mouse. Mean ± SEM is shown. (**D**) Immunoblot analysis demonstrates no cleavage of ATF6 into a 50-kDa fragment or increase of IRE1α phosphorylation in *Cfap418*$^{-/-}$ retinas at P16 and P30. γ-tubulin is a sample loading control.

Supplemental Figure 10: Distribution of PRKCA and phosphorylated PRKCA in developing and mature mouse retinas. Pan- and pT497-PRKCA immunoreactivities are present throughout the retina including various photoreceptor layers at P5 (left) and are mainly located in rod bipolar cells in the retina at P21 (right). Scale bars, 10 μm.

Supplemental Figure 11: Binding of CFAP418 to lipids and lipid changes in *Cfap418*$^{-/-}$ retinas. (**A**) Both His- and GST-tagged mouse CFAP418 proteins bind to phosphatidic acid (PA) on membrane lipid strips. His-CFAP418 also binds to lysophosphatidic acid (LPA) weakly. The lipid arrangements on the two strips are the same. (**B**) Heatmaps show the abundances of individual lipid species in each sample before and after the elimination of the outlier, sample 14. (**C**) Pareto abundance histograms of various lipid categories in *Cfap418*$^{+/-}$ and *Cfap418*$^{-/-}$ retinas at P10. Cumulative lines are shown. The numbers on top of each bin indicate the numbers of lipid species in the bin. Red and black underlines indicate the increased and reduced lipid categories in *Cfap418*$^{-/-}$ retinas. (**D**) Pareto abundance histograms of various acyl chains in *Cfap418*$^{+/-}$ and *Cfap418*$^{-/-}$ retinal membrane lipids at P10. Legends are the same as in **C**. (**E**) The seven PC species that are altered in P10 *Cfap418*$^{-/-}$ retinas. Data from individual mice and mean

± SEM are shown. * and **: $p < 0.05$ and 0.01, respectively, based on Student's t-test on the MetaboAnalyst processed data.

Supplemental Figure 12: Abnormal lipid metabolic enzyme and transporter expression in P10 *Cfap418*$^{-/-}$ retinas. Dot plots show the original MS reading data of individual mice and mean ± SEM. Student's t-test was conducted on the MetaboAnalyst processed data.

Supplemental Figure 13: Expressions of IRD-associated proteins in *Cfap418*$^{-/-}$ retinas. The expressions of proteins encoded by known retinal disease genes (RetNet) and detected in our P5 and P10 quantitative proteomic studies are shown. Totally, 25 proteins are differentially expressed in *Cfap418*$^{-/-}$ retinas. Among them, proteins in the BBSome, OS, and spliceosome tend to be more affected. Note that the clustering of proteins in this figure is based on the current knowledge of functional and direct protein associations. The associations of CFAP418 with BBSome and RAB28 revealed in this study are novel and not shown. The gradient from dark blue to dark red of nodes and node borders (P5 and P10, respectively) indicates the gradient from the most reduced to the most increased fold change in *Cfap418*$^{-/-}$ retinas. Dashed border and square-shaped node denote the statistical significance ($p < 0.05$) at P5 and P10, respectively. The red color of node labels depicts all the DE proteins found in P5 and P10 *Cfap418*$^{-/-}$ retinas. The cellular functions and compartments of protein clusters circled by red lines are labeled.

References:

1. Molday R. Monoclonal antibodies to rhodopsin and other proteins of rod outer segments. *Progress in Retinal Research.* 1988;8:173-209.
2. Sharif AS, et al. C8ORF37 Is Required for Photoreceptor Outer Segment Disc Morphogenesis by Maintaining Outer Segment Membrane Protein Homeostasis. *J Neurosci.* 2018;38(13):3160-76.

3. Ying G, et al. The small GTPase RAB28 is required for phagocytosis of cone outer segments by the murine retinal pigmented epithelium. *J Biol Chem.* 2018;293(45):17546-58.
4. Wang L, et al. Whirlin interacts with espin and modulates its actin-regulatory function: an insight into the mechanism of Usher syndrome type II. *Human Molecular Genetics.* 2012;21(3):692-710.
5. Jung SY, et al. An Anatomically Resolved Mouse Brain Proteome Reveals Parkinson Disease-relevant Pathways. *Molecular & cellular proteomics : MCP.* 2017;16(4):581-93.
6. Saltzman AB, et al. gpGrouper: A Peptide Grouping Algorithm for Gene-Centric Inference and Quantitation of Bottom-Up Proteomics Data. *Molecular & cellular proteomics : MCP.* 2018;17(11):2270-83.
7. Navarrete-Perea J, et al. Streamlined Tandem Mass Tag (SL-TMT) Protocol: An Efficient Strategy for Quantitative (Phospho)proteome Profiling Using Tandem Mass Tag-Synchronous Precursor Selection-MS3. *J Proteome Res.* 2018;17(6):2226-36.
8. Schweppe DK, et al. Full-Featured, Real-Time Database Searching Platform Enables Fast and Accurate Multiplexed Quantitative Proteomics. *J Proteome Res.* 2020;19(5):2026-34.
9. Elias JE, and Gygi SP. Target-decoy search strategy for increased confidence in large-scale protein identifications by mass spectrometry. *Nat Methods.* 2007;4(3):207-14.
10. Huttlin EL, et al. A tissue-specific atlas of mouse protein phosphorylation and expression. *Cell.* 2010;143(7):1174-89.
11. Eng JK, et al. Comet: an open-source MS/MS sequence database search tool. *Proteomics.* 2013;13(1):22-4.
12. McAlister GC, et al. MultiNotch MS3 enables accurate, sensitive, and multiplexed detection of differential expression across cancer cell line proteomes. *Anal Chem.* 2014;86(14):7150-8.
13. Matyash V, et al. Lipid extraction by methyl-tert-butyl ether for high-throughput lipidomics. *J Lipid Res.* 2008;49(5):1137-46.
14. Shevchenko A, et al. Mass spectrometric sequencing of proteins silver-stained polyacrylamide gels. *Anal Chem.* 1996;68(5):850-8.
15. Peng J, and Gygi SP. Proteomics: the move to mixtures. *J Mass Spectrom.* 2001;36(10):1083-91.
16. Eng JK, et al. An approach to correlate tandem mass spectral data of peptides with amino acid sequences in a protein database. *J Am Soc Mass Spectrom.* 1994;5(11):976-89.
17. Chong J, et al. Using MetaboAnalyst 4.0 for Comprehensive and Integrative Metabolomics Data Analysis. *Curr Protoc Bioinformatics.* 2019;68(1):e86.
18. Reimand J, et al. Pathway enrichment analysis and visualization of omics data using g:Profiler, GSEA, Cytoscape and EnrichmentMap. *Nat Protoc.* 2019;14(2):482-517.
19. Heberle H, et al. InteractiVenn: a web-based tool for the analysis of sets through Venn diagrams. *BMC Bioinformatics.* 2015;16:169.
20. Pang Z, et al. MetaboAnalyst 5.0: narrowing the gap between raw spectra and functional insights. *Nucleic Acids Res.* 2021;49(W1):W388-W96.

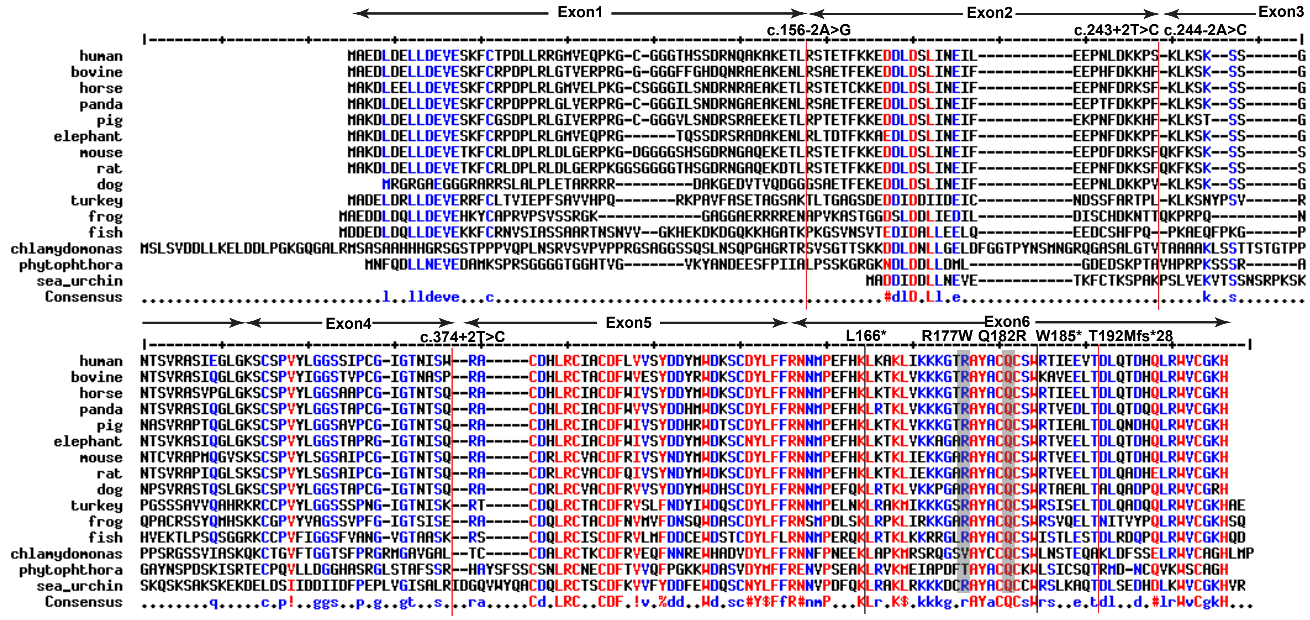

Figure S1: CFAP418 protein sequence alignment across different species. Red, blue, and black residues represent highly, mildly, and not conserved residues, respectively. Patient mutations are shown on the top of sequences. Splice site, nonsense, and frameshift mutations are denoted by vertical lines, and missense mutations are highlighted in grey.

GOCC: Photoreceptor IS (P5)

NES: -1.820; *p* val: 0.002; FDR q: 0.045

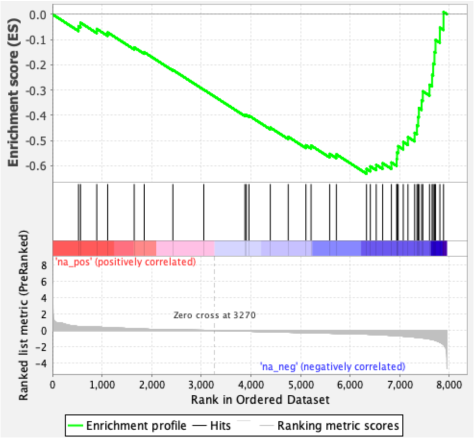

GOBP: Phototransduction (P5)

NES: -1.773; *p* val: 0; FDR q: 0.067

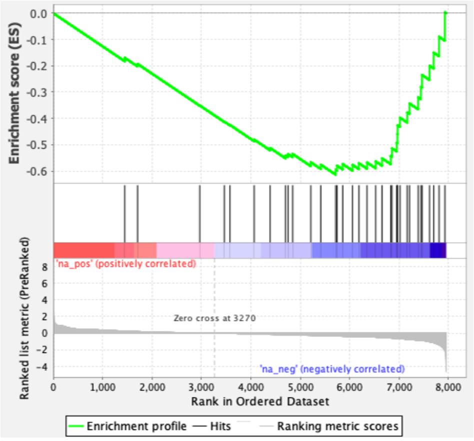

Ciliary Membrane (P10)

NES: -1.993; p val: 0; FDR q: 0.013

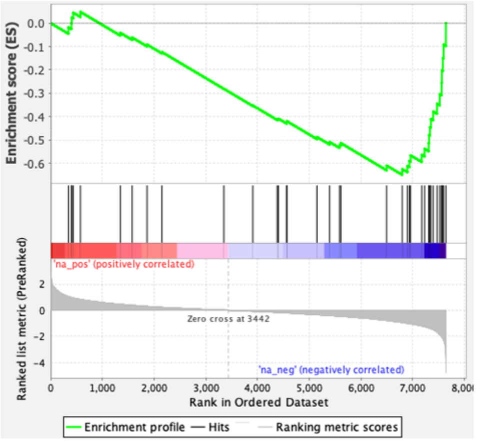

Figure S2: GSEA reveals a reduction of photoreceptor proteins and pathways in *Cfap418$^{-/-}$* retinas. Photoreceptor IS and phototransduction proteins are down-regulated in P5 *Cfap418$^{-/-}$* retinas. Ciliary membrane proteins including photoreceptor OS membrane proteins are down-regulated in P10 *Cfap418$^{-/-}$* retinas.

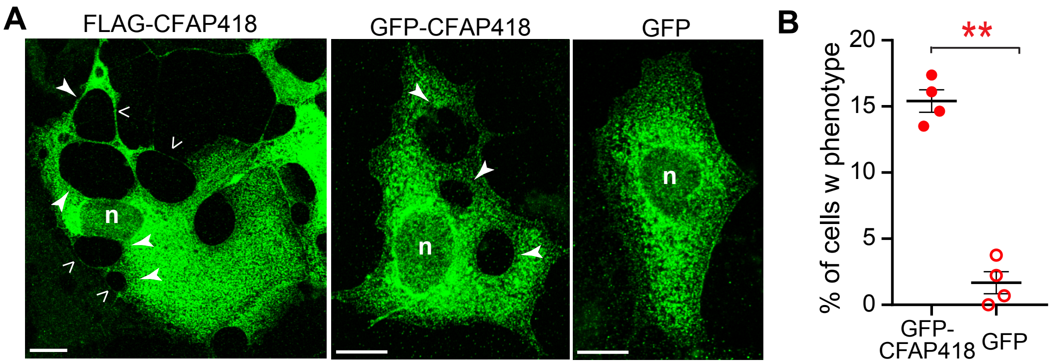

Figure S3: Vacuole accumulation in CFAP418-transfected cells. (**A**) Representative COS-7 cells transfected with FLAG- and GFP-CFAP418, but not GFP, accumulate large vacuoles (filled arrows). Open arrows indicate thin edges of vacuoles. n: nucleus. Scale bars: 10 μm. (**B**) Quantification of the vacuole accumulation phenotype in GFP-CFAP418- and GFP-transfected COS-7 cells. Dot plot represents data from individual experiments and mean ± SEM. **, $p < 0.01$ (Student's t-test).

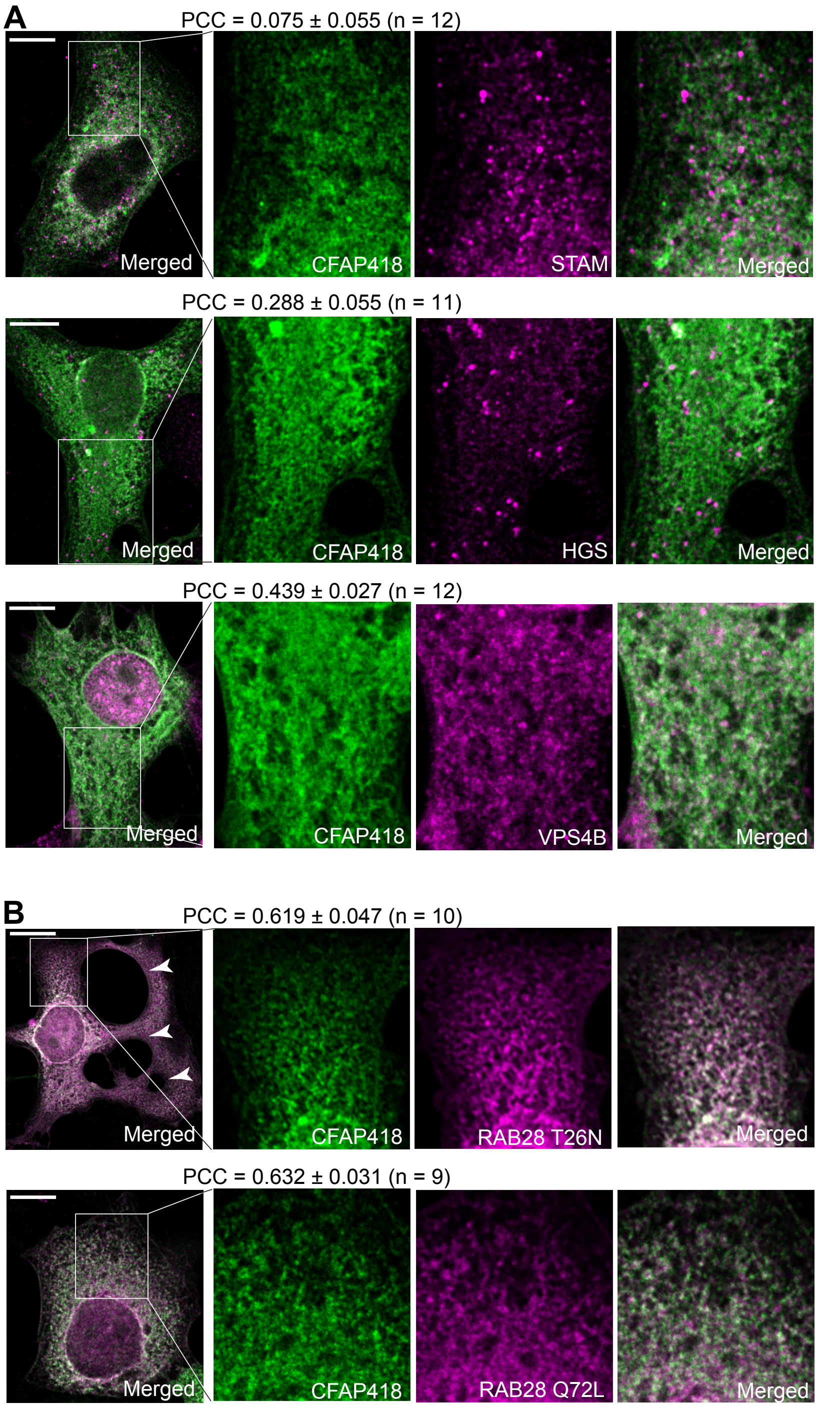

Figure S4: Colocalization analysis of CFAP418 with ESCRT and RAB28 proteins. (**A**) Representative COS-7 cells transfected with FLAG-CFAP418 and double-immunostained for FLAG and ESCRT proteins. (**B**) Representative COS-7 cells double-transfected with FLAG-CFAP418 and mCherry-RAB28 mutant proteins. Cells were immunostained using FLAG and mCherry antibodies. Arrows point to the abnormally accumulated vacuoles. Framed regions are amplified and shown on the right with merged and individual channels. The PCCs are shown as mean ± SEM. n, number of cells analyzed. Scale bars: 10 μm.

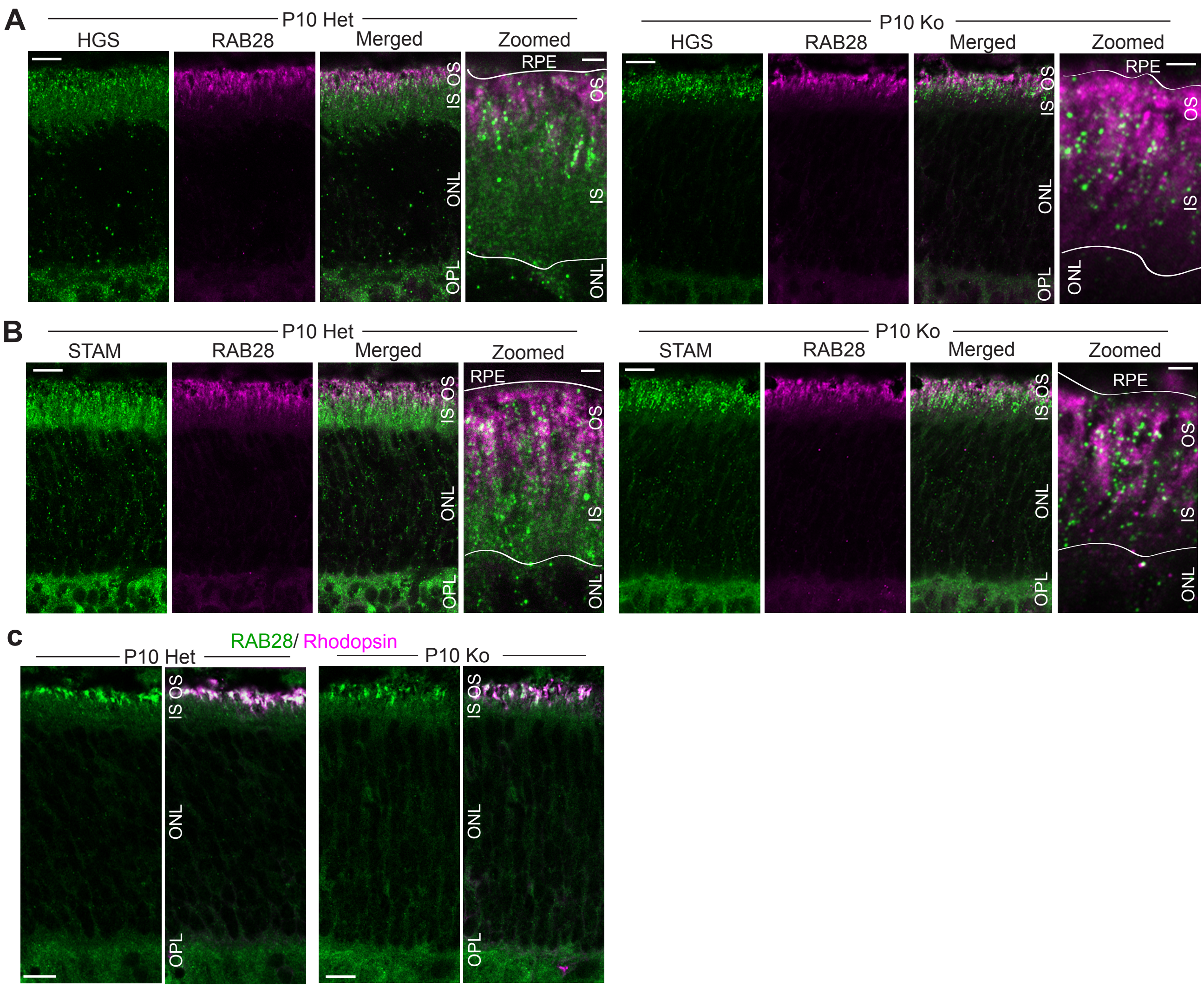

Figure S5: Abnormal HGS and STAM but normal RAB28 distributions in P10 *Cfap418*$^{-/-}$ photoreceptors. (**A**) HGS and (**B**) STAM, but not RAB28, signal patterns are different in P10 *Cfap418*$^{+/-}$ and *Cfap418*$^{-/-}$ photoreceptors. White lines outline the RPE and OS border and the IS and ONL border, based on the corresponding bright field channels. The zoomed images on the right are independent images from the ones on the left. (**C**) Double immunostaining for RAB28 and rhodopsin in P10 *Cfap418*$^{+/-}$ and *Cfap418*$^{-/-}$ photoreceptors. As reported previously (3), RAB28 is in photoreceptor OS, IS, and OPL. The strongest RAB28 signal is present in the OS, marked by rhodopsin. Scale bars: 2 μm (zoomed) and 10 μm (others).

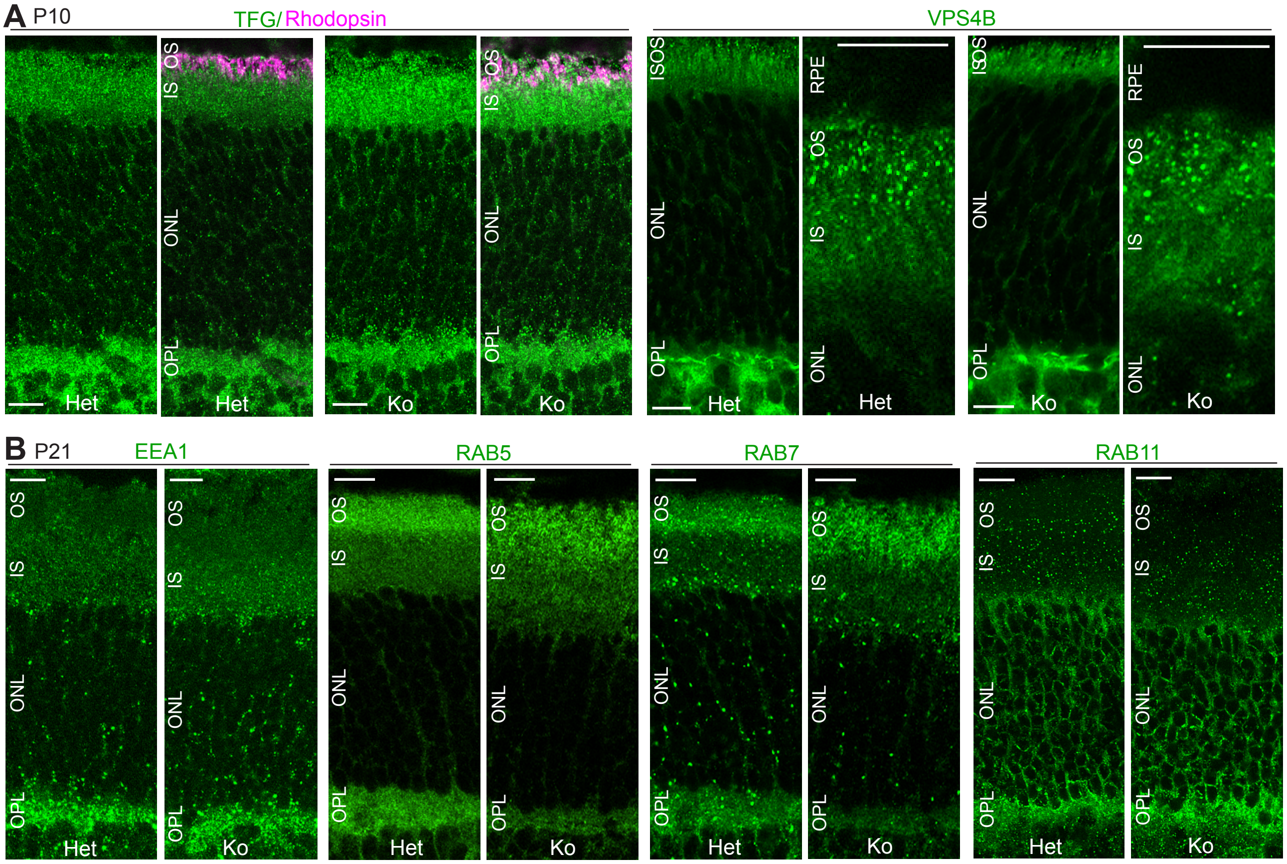

Figure S6: Localization of vesicular trafficking proteins in *Cfap418*$^{-/-}$ photoreceptors. (**A**) TFG and VPS4B proteins are localized normally in *Cfap418*$^{-/-}$ photoreceptors, compared with littermate *Cfap418*$^{+/-}$ photoreceptors at P10. (**B**) Early endosomal proteins EEA1 and RAB5, late endosomal protein RAB7, and recycling endosomal protein RAB11 appear to be distributed normally in *Cfap418*$^{-/-}$ photoreceptors, compared with littermate *Cfap418*$^{+/-}$ photoreceptors at P21. Scale bars: 10 μm.

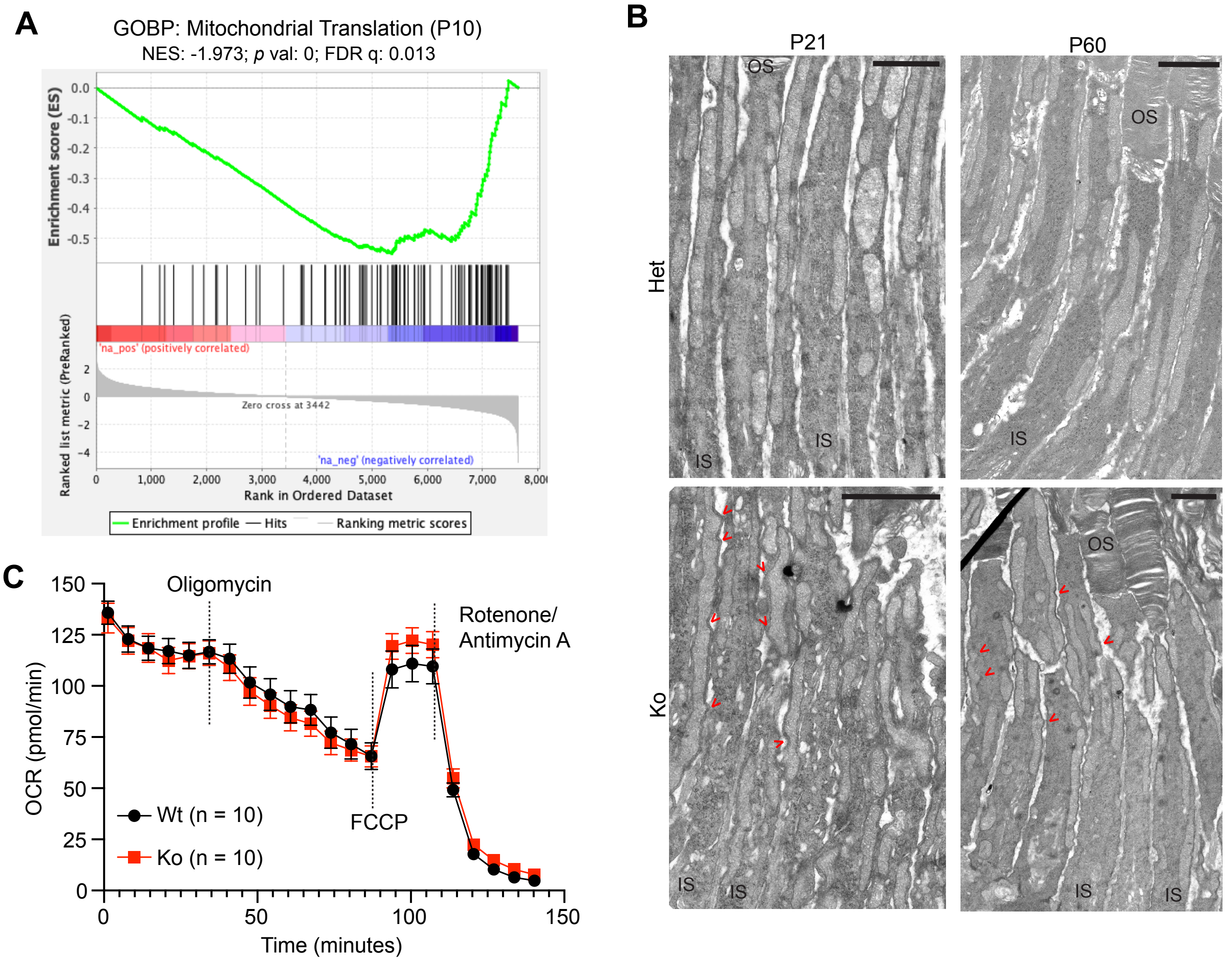

Figure S7: Abnormal mitochondrial protein expression and morphology but normal mitochondrial respiration in *Cfap418*$^{-/-}$ retinas. (**A**) GSEA shows mitochondrial translation proteins are enriched in the down-regulated proteins in P10 *Cfap418*$^{-/-}$ retinas.(**B**) A longitudinal view of *Cfap418*$^{-/-}$ photoreceptors shows uneven diameters along the length of mitochondria at P21 and P60, compared with the smooth straight long bar-shaped mitochondria in *Cfap418*$^{+/-}$ littermate photoreceptors. Red arrows point to the abnormal constrictions and protruding bumps of the mitochondria. Scale bars, 1.5 μm. (**C**) OCR is comparable between *Cfap418*$^{+/+}$ and *Cfap418*$^{-/-}$ littermate retinas at 3-5 weeks of age. Each data point is the mean ± SEM from 10 mice, and the datum of each mouse is the mean of 4 retinal punches from two eyes. Repeated measures two-way ANOVA was conducted with genotype as a between-subject variable and time as a within-subject variable.

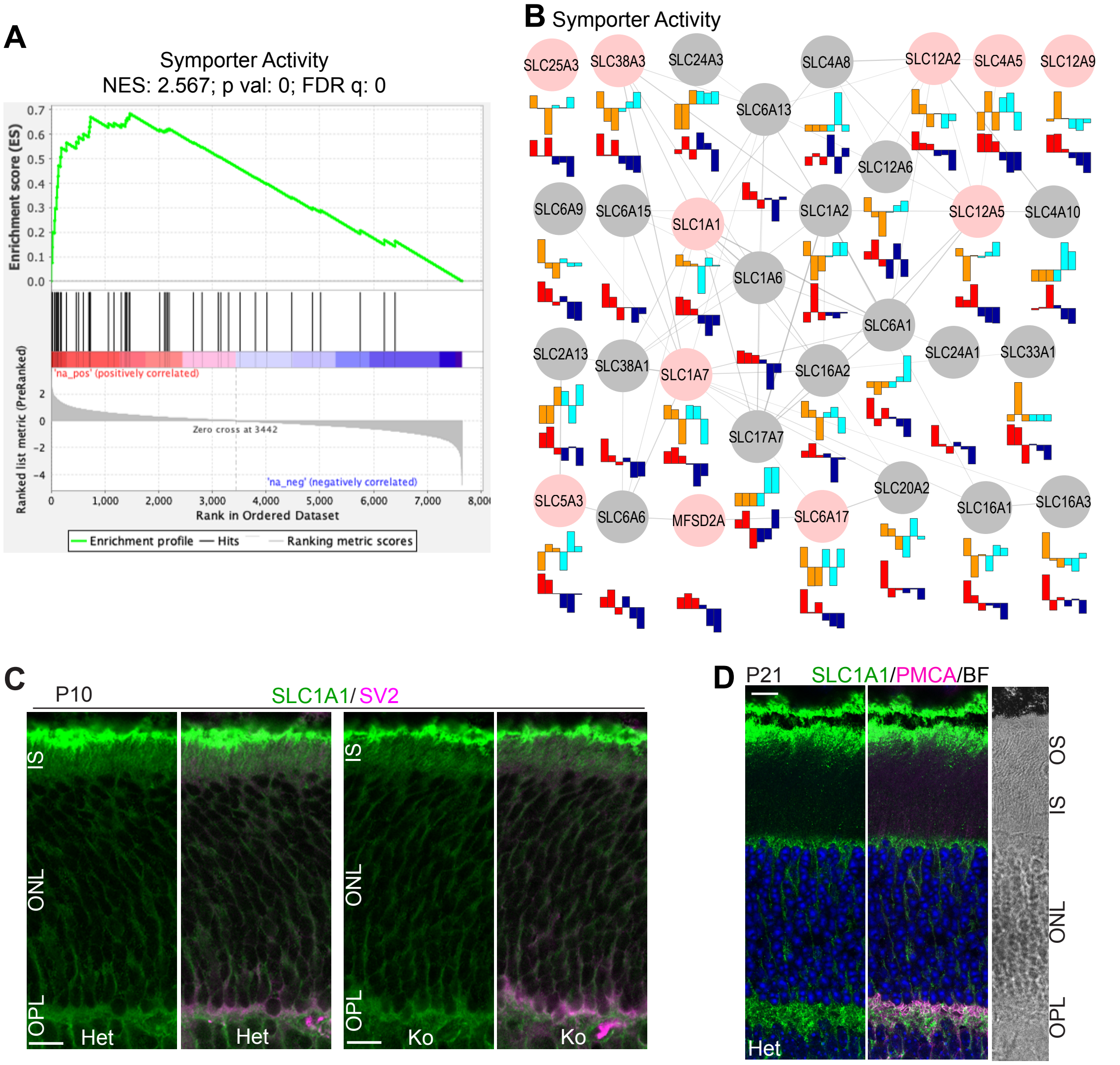

Figure S8: Symporter expression is increased in P10 *Cfap418*$^{-/-}$ retinas. (**A**) GSEA identified increased symporter activity in P10 *Cfap418*$^{-/-}$ retinas. (**B**) The expressions of leading symporters, revealed by GSEA, in *Cfap418*$^{+/+}$ and *Cfap418*$^{-/-}$ retinas at P5 and P10. Legends are the same as those in Figure 2B. (**C**) SLC1A1 is present in all photoreceptor layers at P10. SV2 labels the photoreceptor OPL. There is no significant difference in SLC1A1 distribution between P10 *Cfap418*$^{+/-}$ and *Cfap418*$^{-/-}$ photoreceptors. (**D**) SLC1A1 is present in OS apex, ONL, and OPL in P21 photoreceptors. OPL is marked by PMCA. Note that there is a tissue separation between OS and RPE in these images, as shown in the bright field (BF) channel. Scale bars, 10 μm.

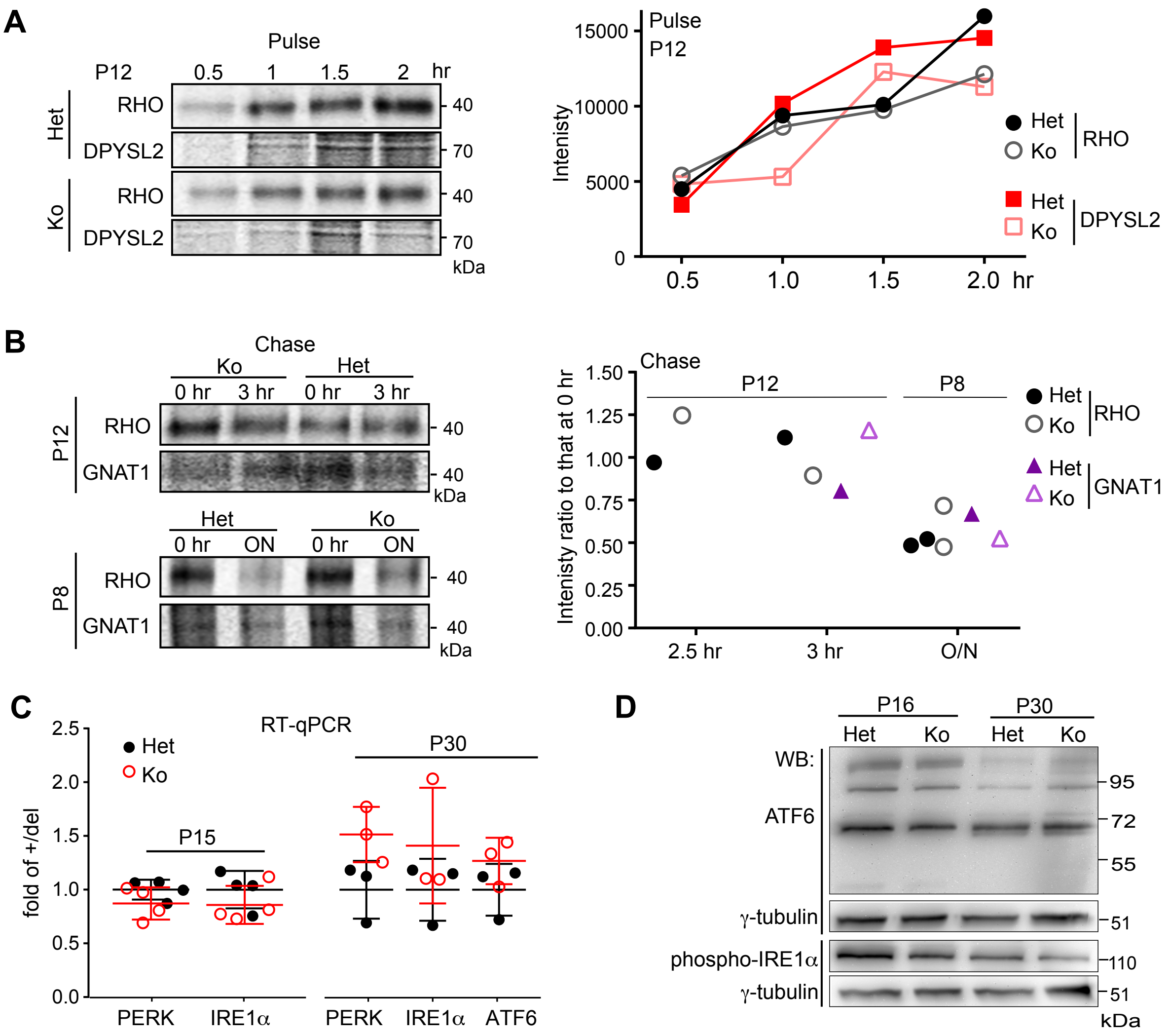

Figure S9: Evaluation of OS membrane protein synthesis, degradation, and ER stress in *Cfap418*$^{-/-}$ retinas. (**A**) Pulse labeling using [35S] methionine for up to 2 hours shows no obvious reduction in newly synthesized rhodopsin (RHO) in *Cfap418*$^{-/-}$ retinas at P12, compared with *Cfap418*$^{+/-}$ littermate retinas. DPYSL2 is a cytoplasmic protein and was used as a negative control. Left, SDS-PAGE autoradiograms of pulse-labeled RHO and DPYSL2 proteins immunoprecipitated from retinal explants. Right, quantification of the RHO and DPYSL2 radioactivity from the autoradiograms. (**B***)* Chase labeling of *Cfap418*$^{+/-}$ and *Cfap418*$^{-/-}$ littermate retinal explants for 3 hours at P12 and overnight at P8 using [35S] methionine shows no obvious changes in the degradation of RHO or transducin α subunit (GNAT1). Left, representative SDS-PAGE autoradiograms of chase-labeled RHO and GNAT1 proteins immunoprecipitated from retinal explants. Right, quantitation of radioactive RHO and GNAT1 signals. (**C**) RT-qPCR analysis shows no changes in the PERK, IRE1α, and ATF6 mRNAs in *Cfap418*$^{-/-}$ retinas at P15 and P30. Each dot represents the retinas of an individual mouse. Mean ± SEM is shown. (**D**) Immunoblot analysis demonstrates no cleavage of ATF6 into a 50-kDa fragment or increase of IRE1α phosphorylation in *Cfap418*$^{-/-}$ retinas at P16 and P30. γ-tubulin is a sample loading control.

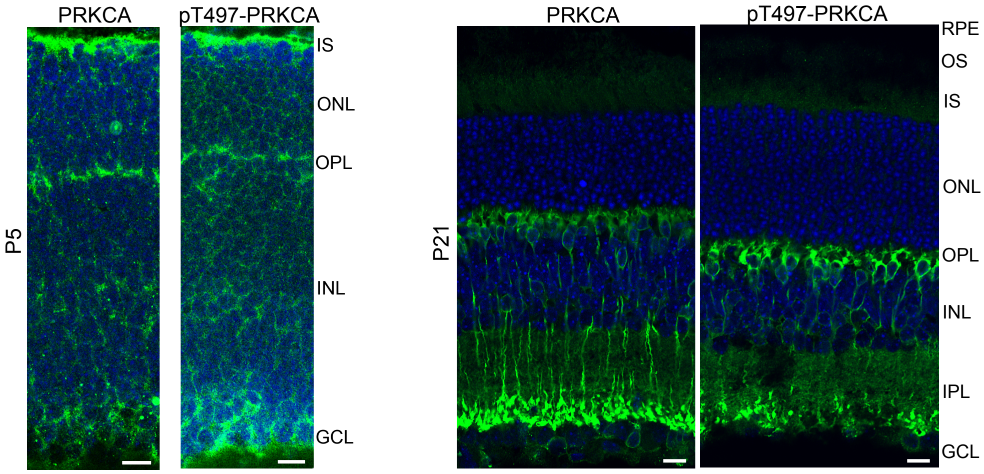

Figure S10: Distribution of PRKCA and phosphorylated PRKCA in developing and mature mouse retinas. Pan- and pT497-PRKCA immunoreactivities are present throughout the retina including various photoreceptor layers at P5 (left) and are mainly located in rod bipolar cells in the retina at P21 (right). Scale bars, 10 μm.

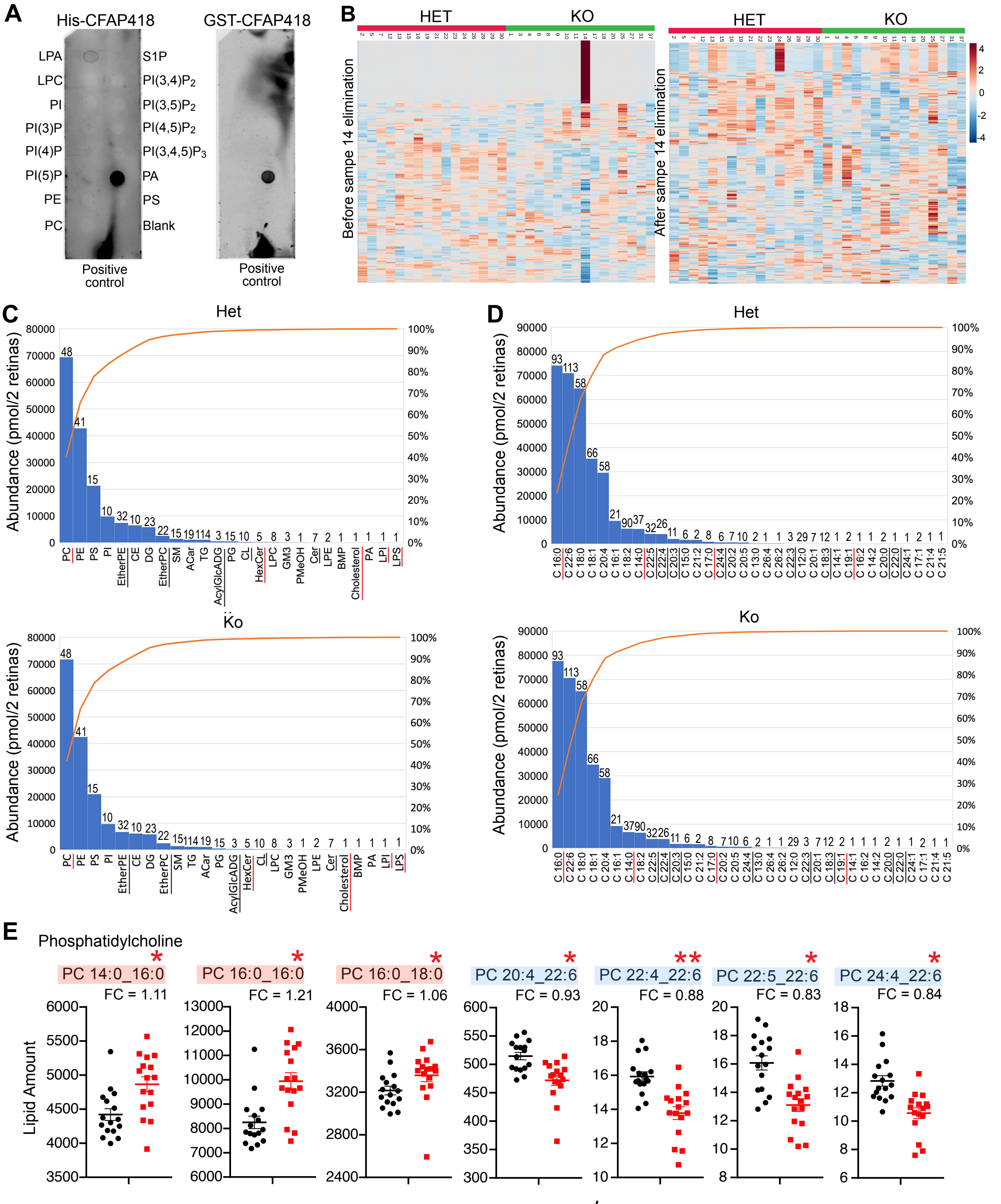

Figure S11: Binding of CFAP418 to lipids and lipid changes in *Cfap418*$^{-/-}$ retinas. (**A**) Both His- and GST-tagged mouse CFAP418 proteins bind to phosphatidic acid (PA) on membrane lipid strips. His-CFAP418 also binds to lysophosphatidic acid (LPA) weakly. The lipid arrangements on the two strips are the same. (**B**) Heatmaps show the abundances of individual lipid species in each sample before and after the elimination of the outlier, sample 14. (**C**) Pareto abundance histograms of various lipid categories in *Cfap418*$^{+/-}$ and *Cfap418*$^{-/-}$ retinas at P10. Cumulative lines are shown. The numbers on top of each bin indicate the numbers of lipid species in the bin. Red and black underlines indicate the increased and reduced lipid categories in *Cfap418*$^{-/-}$ retinas. (**D**) Pareto abundance histograms of various acyl chains in *Cfap418*$^{+/-}$ and *Cfap418*$^{-/-}$ retinal membrane lipids at P10. Legends are the same as in **C**. (**E**) The seven PC species that are altered in P10 *Cfap418*$^{-/-}$ retinas. Data from individual mice and mean ± SEM are shown. * and **: $p < 0.05$ and 0.01, respectively, based on Student's t-test on the MetaboAnalyst processed data.

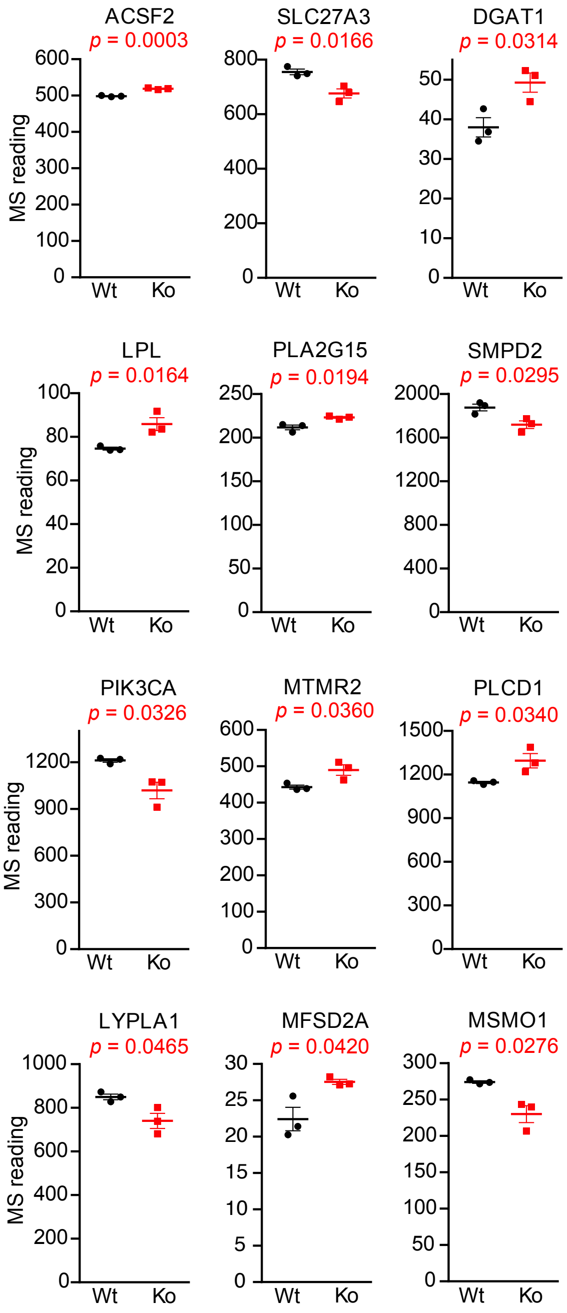

Figure S12: Abnormal lipid metabolic enzyme and transporter expression in P10 *Cfap418*$^{-/-}$ retinas. Dot plots show the origianl MS reading data of individual mice and mean ± SEM. Student's t-test was conducted on the MetaboAnalyst processed data.

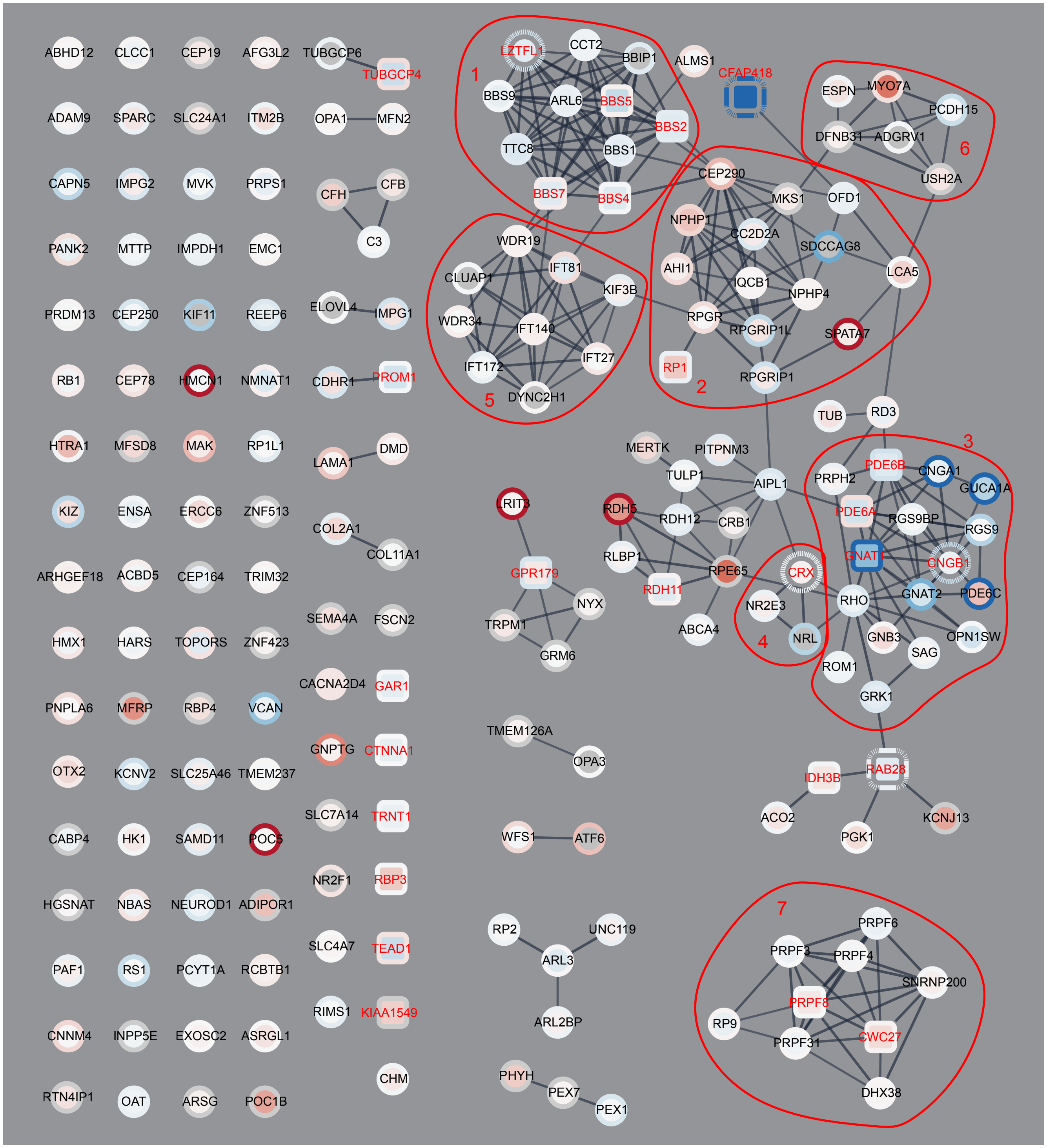

1. BBSome proteins
2. Connecting cilium proteins
3. Outer segment proteins
4. Transcription factors
5. Intraflagellar transport proteins
6. Usher proteins
7. Spliceosome proteins

Figure S13: Expressions of IRD-associated proteins in *Cfap418*$^{-/-}$ retinas. The expressions of proteins encoded by known retinal disease genes (RetNet) and detected in our P5 and P10 quantitative proteomic studies are shown. Totally, 25 proteins are differentially expressed in *Cfap418*$^{-/-}$ retinas. Among them, proteins in the BBSome, OS, and spliceosome tend to be more affected. Note that the clustering of proteins in this figure is based on the current knowledge of functional and direct protein associations. The associations of CFAP418 with BBSome and RAB28 revealed in this study are novel and not shown. The gradient from dark blue to dark red of nodes and node borders (P5 and P10, respectively) indicates the gradient from the most reduced to the most increased fold change in *Cfap418*$^{-/-}$ retinas. Dashed border and square-shaped node denote the statistical significance ($p < 0.05$) at P5 and P10, respectively. The red color of node labels depicts all the DE proteins found in P5 and P10 *Cfap418*$^{-/-}$ retinas. The cellular functions and compartments of protein clusters circled by red lines are labeled.

Full unedited blots for Figure 5C --BBS2

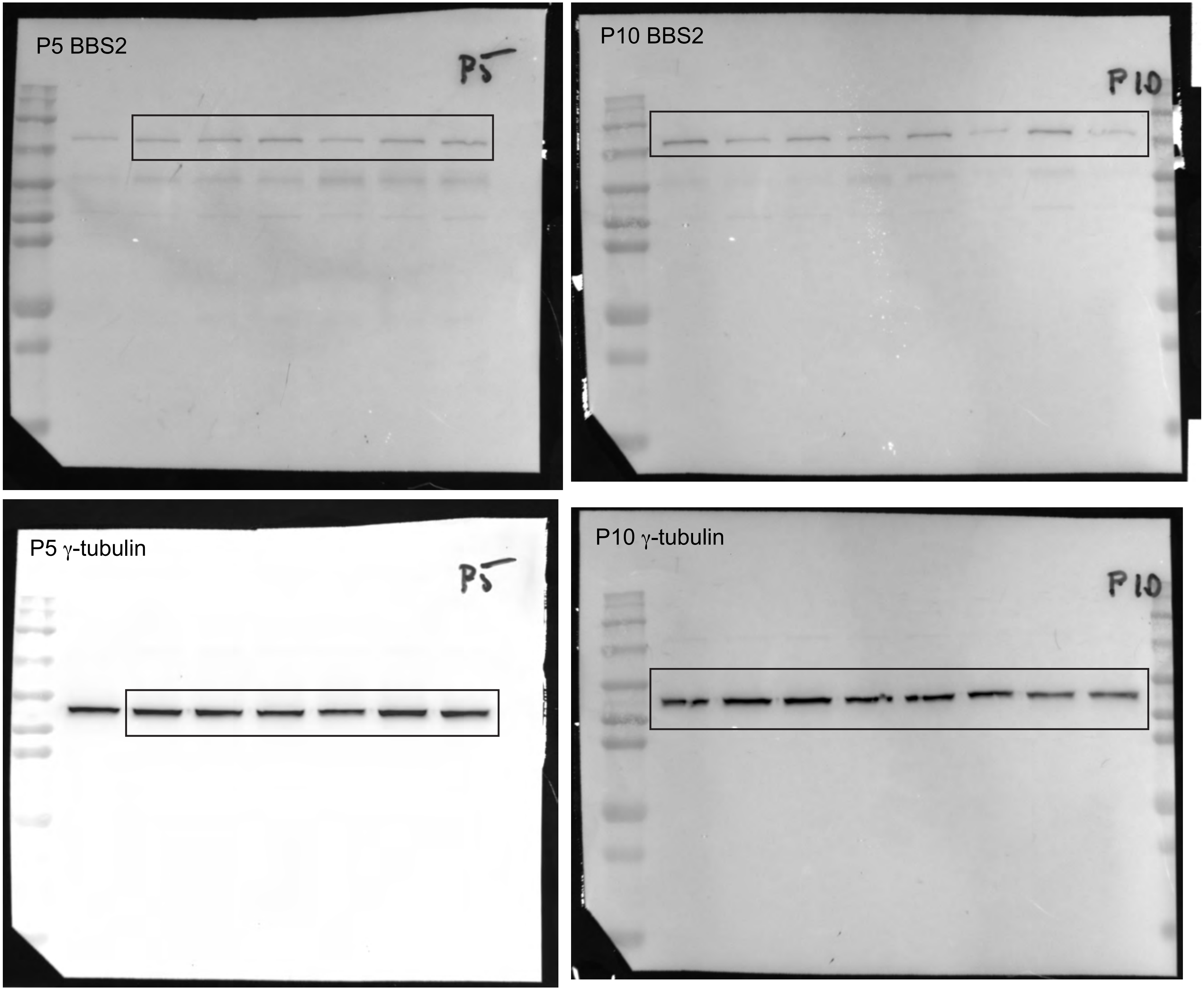

Full unedited blots for Figure 5C -- BBS4

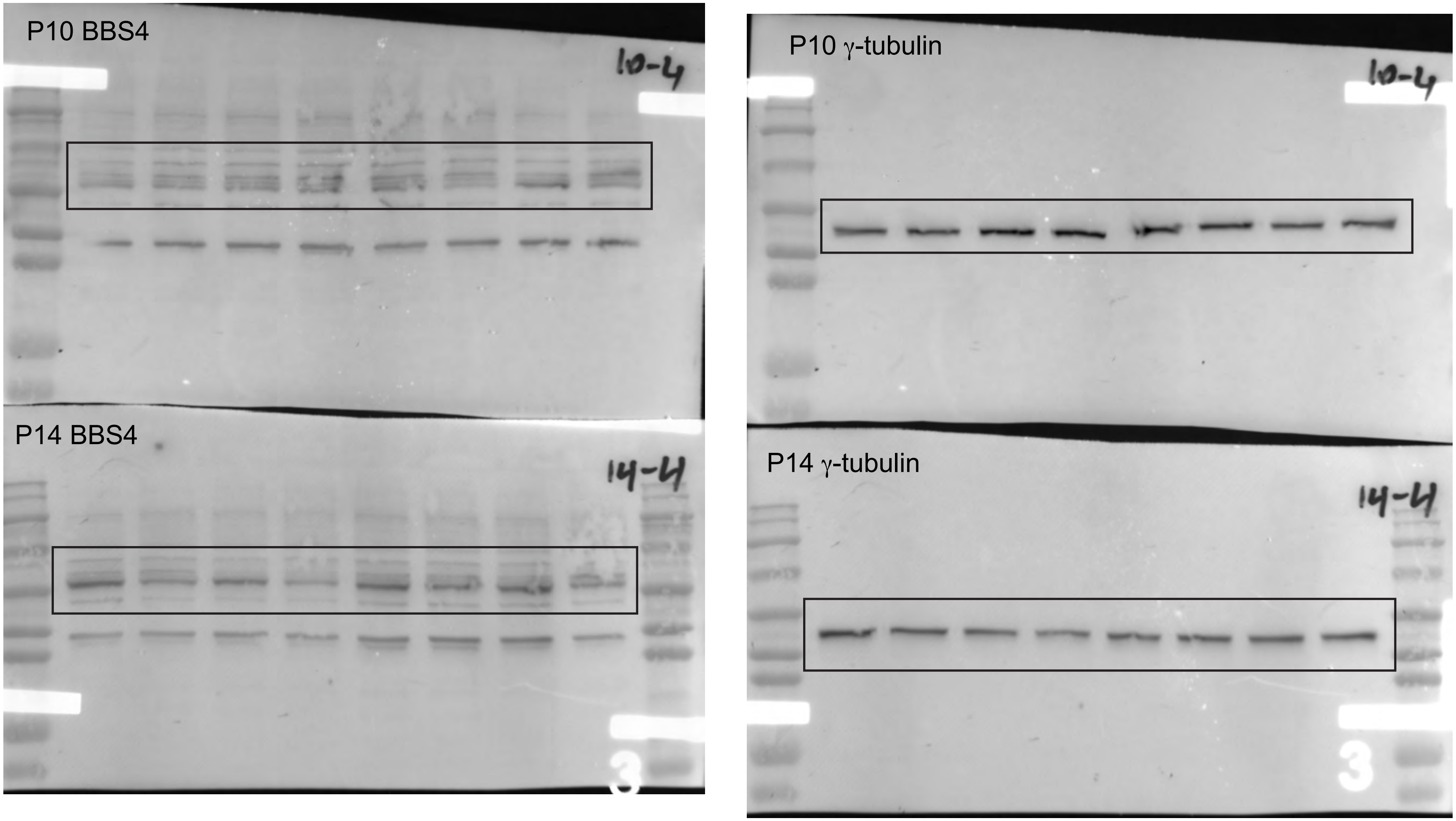

Full unedited blots for Figure 5C --ARL13B

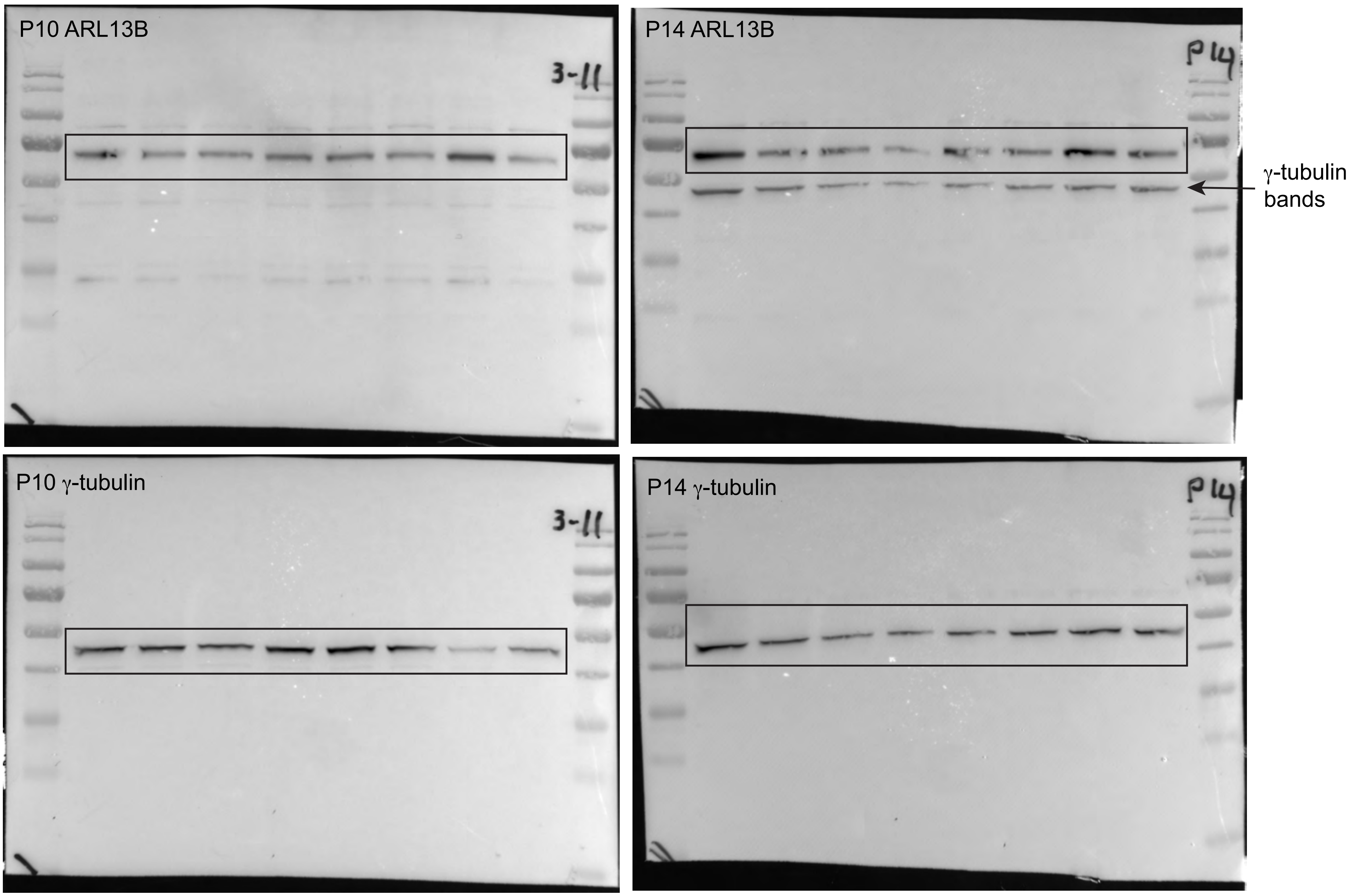

Full unedited blots for Figure 6C

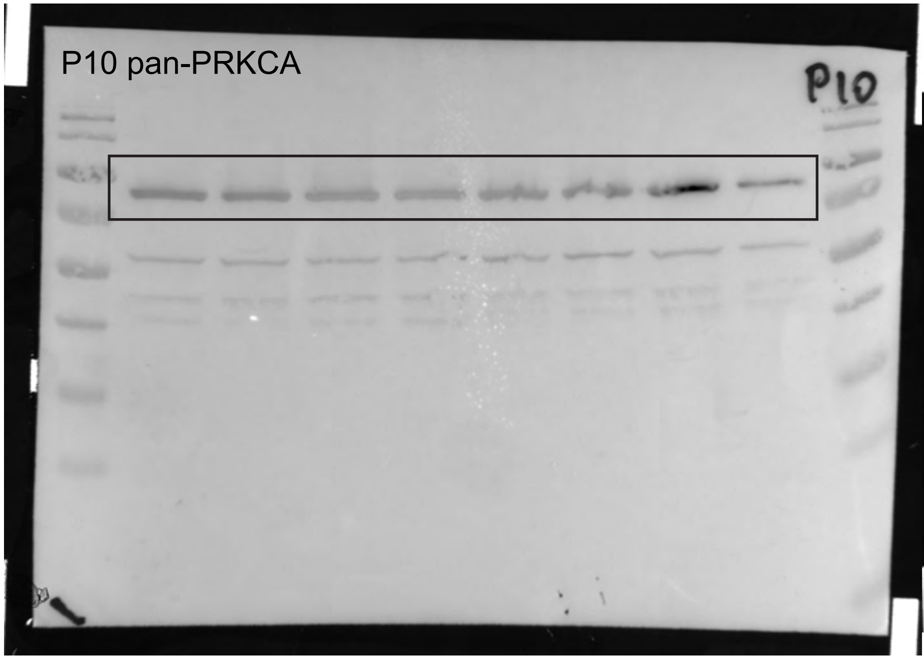

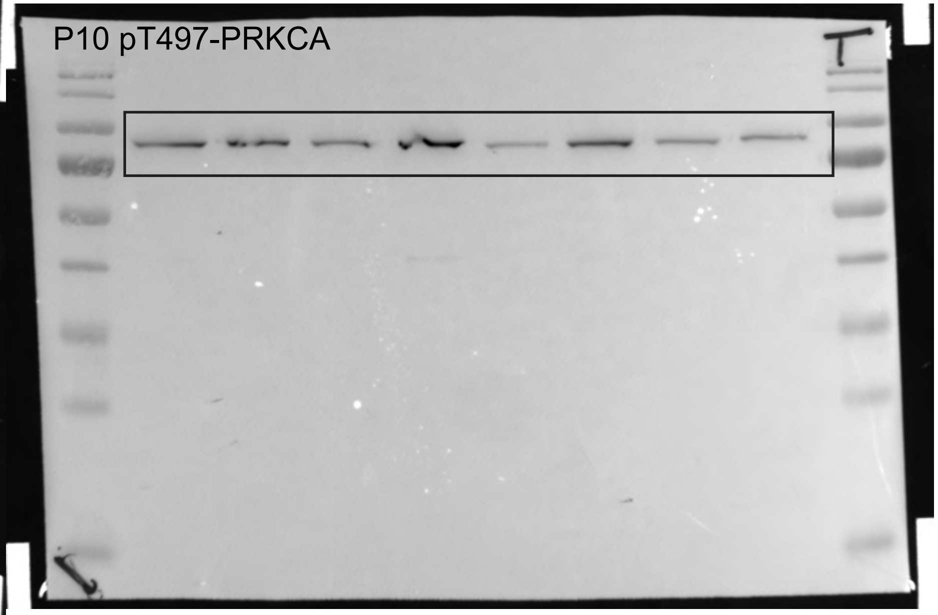

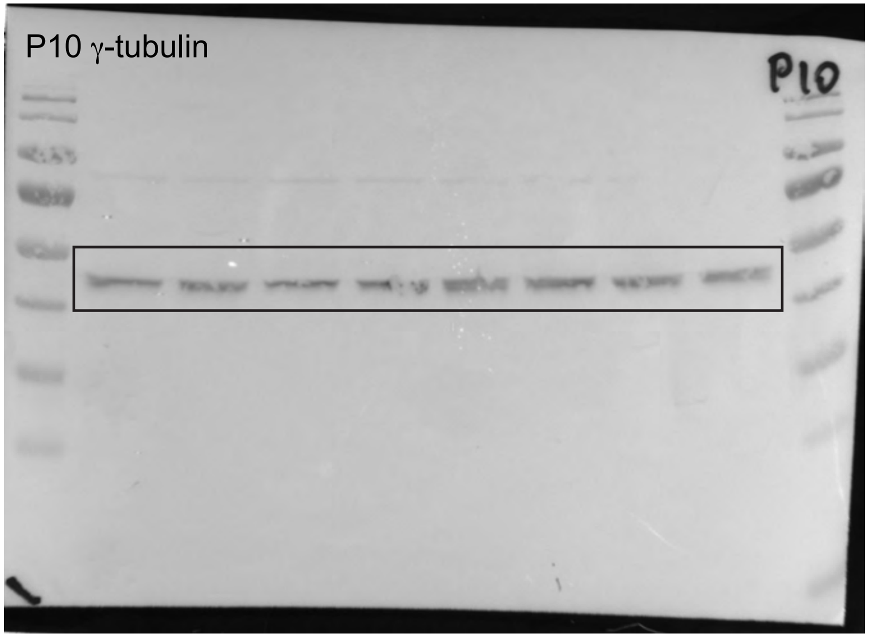

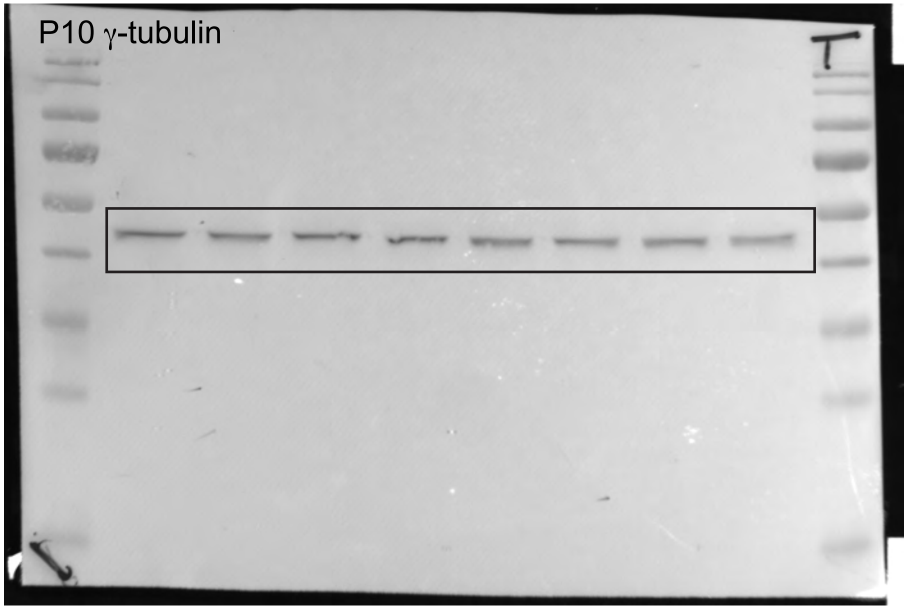

Full unedited blots for Figure 7D

CFAP418 Input

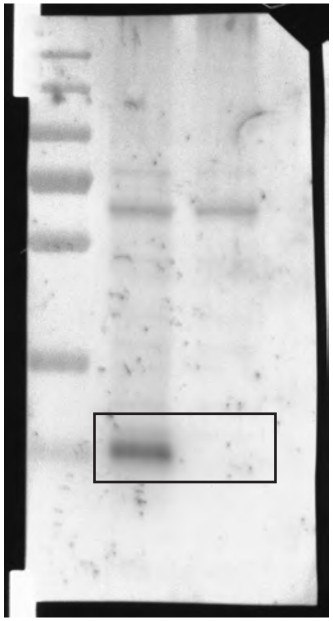

CFAP418 IP

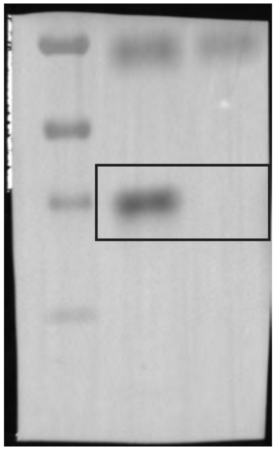

RAB28

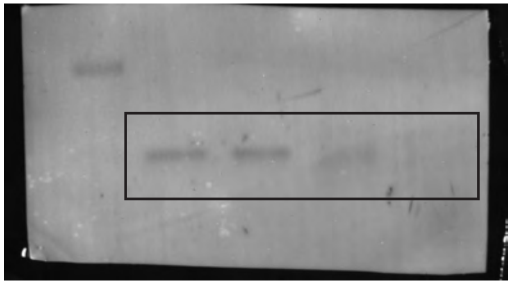

Full unedited blots for Figure 7E

CFAP418

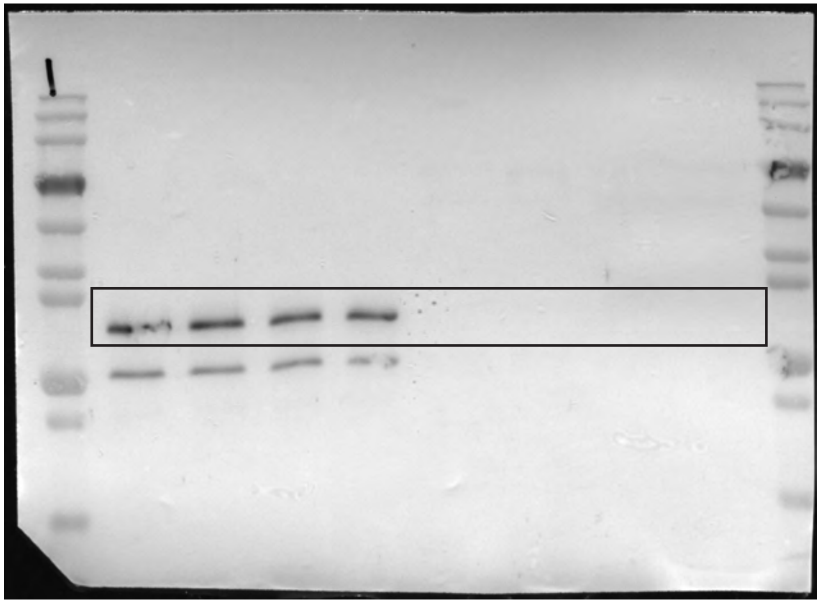

Same blot as left without membrane image overlay, increased constract

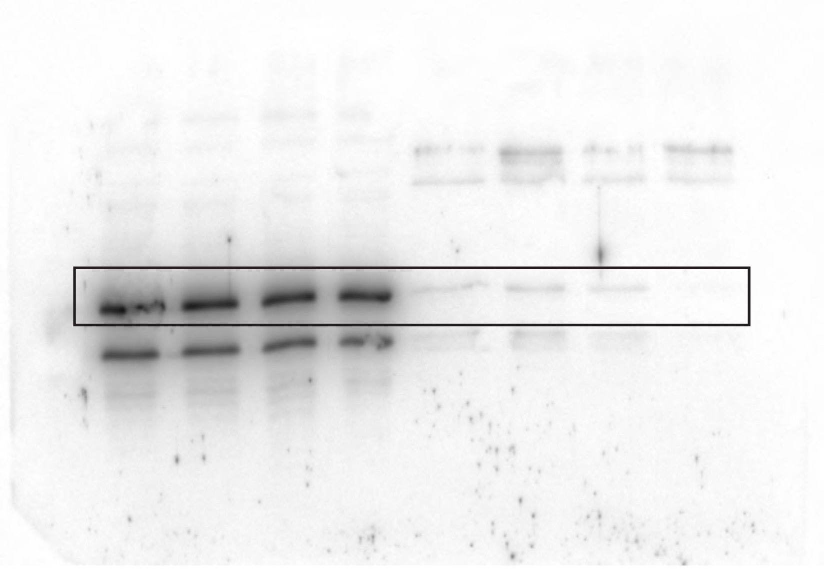

mCherry input overlaid with membrane image (left) and not (right)

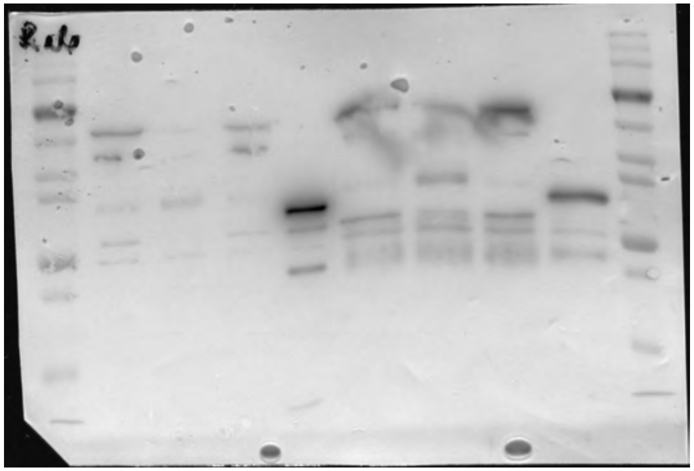

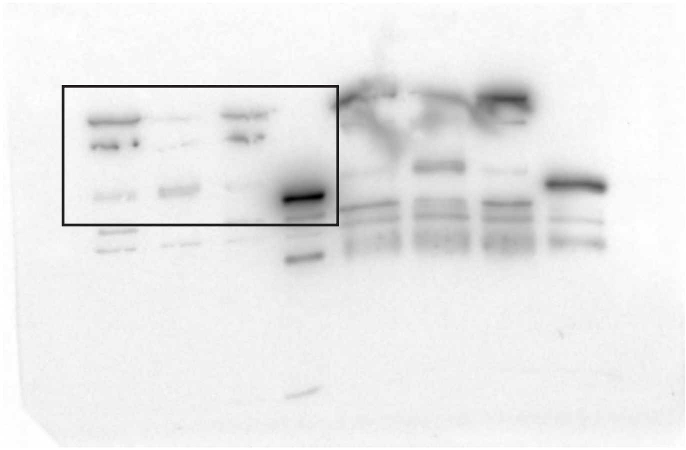

mCherry IP

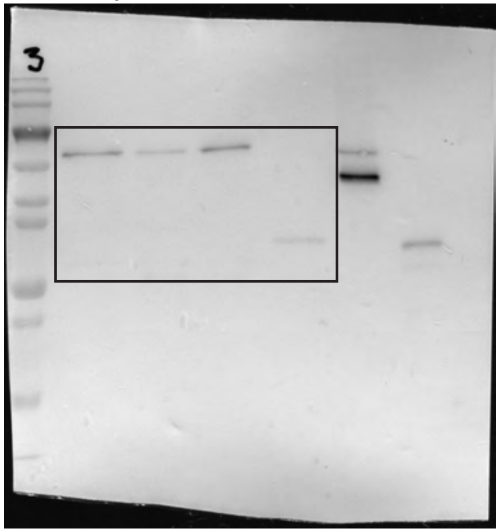

Full unedited blots for Figure 9D

RAB28

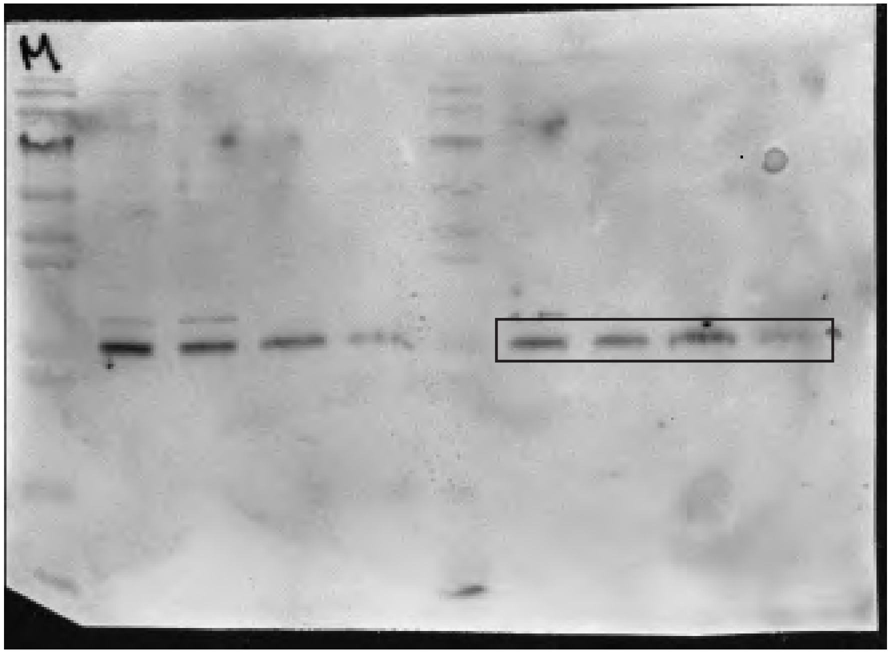

TOR1A

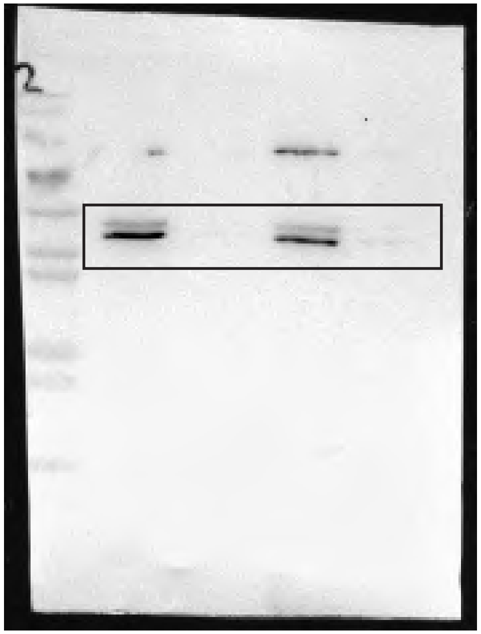

SLC12A5

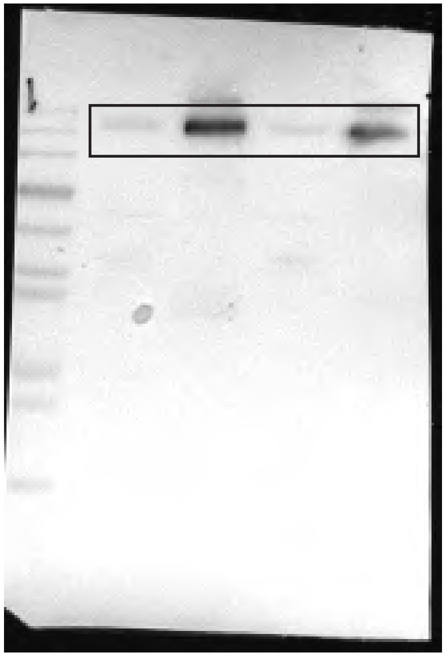

CFAP418

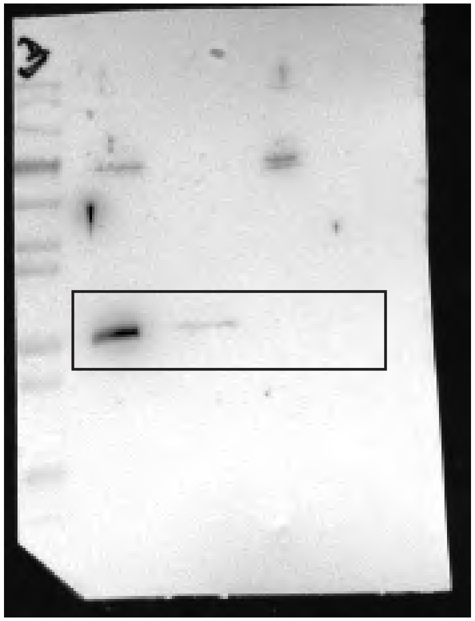

Full unedited blots for Figure 9E

RAB28

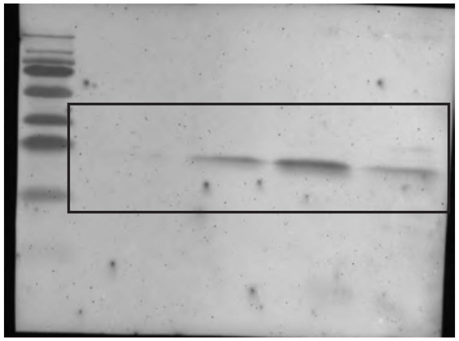

CFAP418

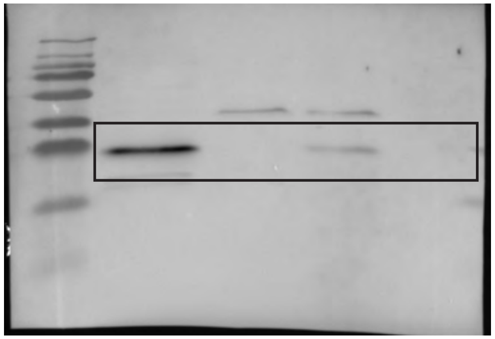

Full unedited gels for Figure S9A

P12 RHO

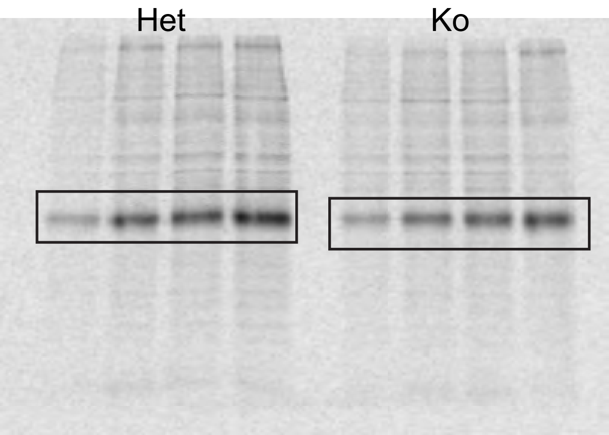

P12 DPYSL2

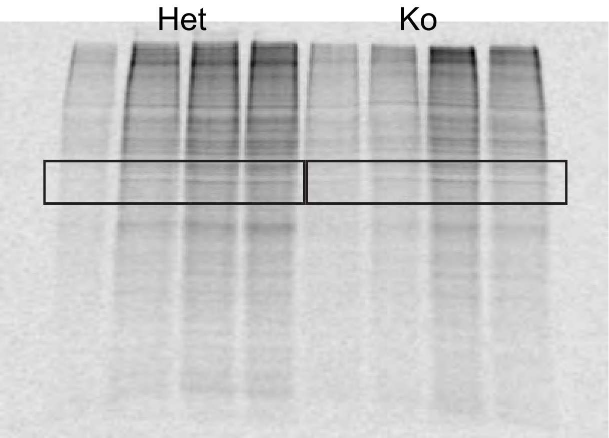

Full unedited gels for Figure S9B

P12 RHO and GNAT1 3hr

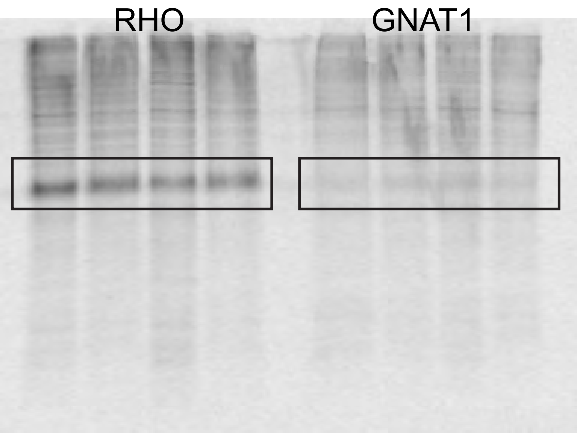

P8 RHO and GNAT1 ON

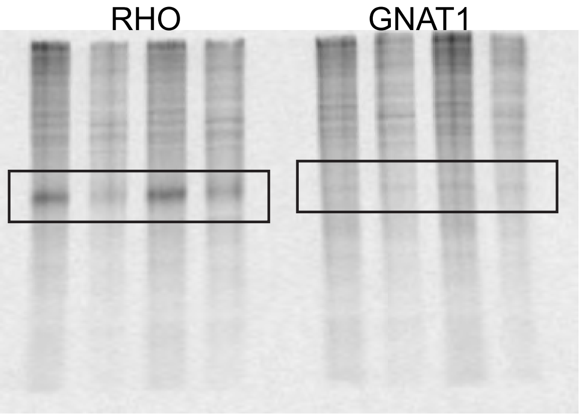

Full unedited blots for Figure S9D

P16 and P30 ATF6 P16 and P30 phospho-IRE1α

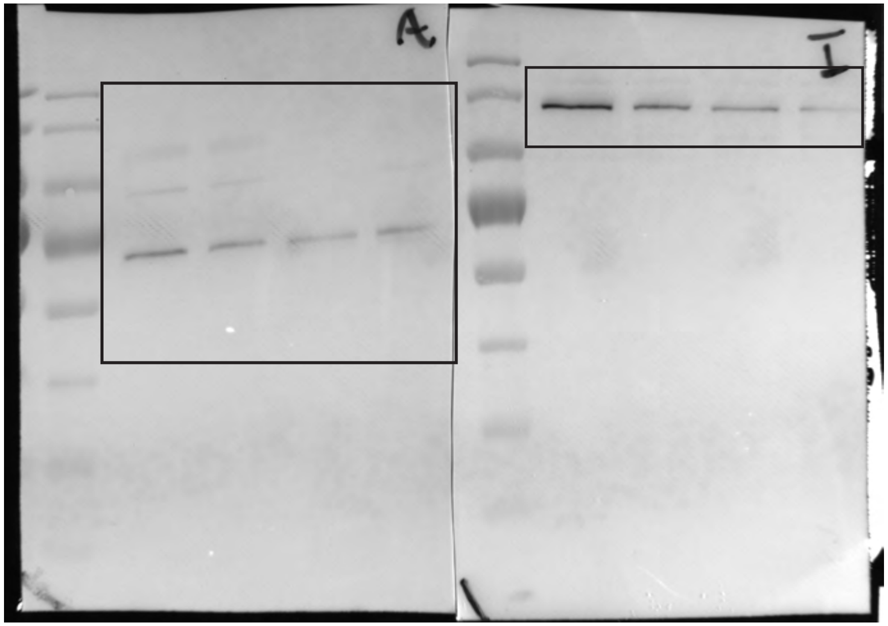

P16 and P30 γ-tubulin

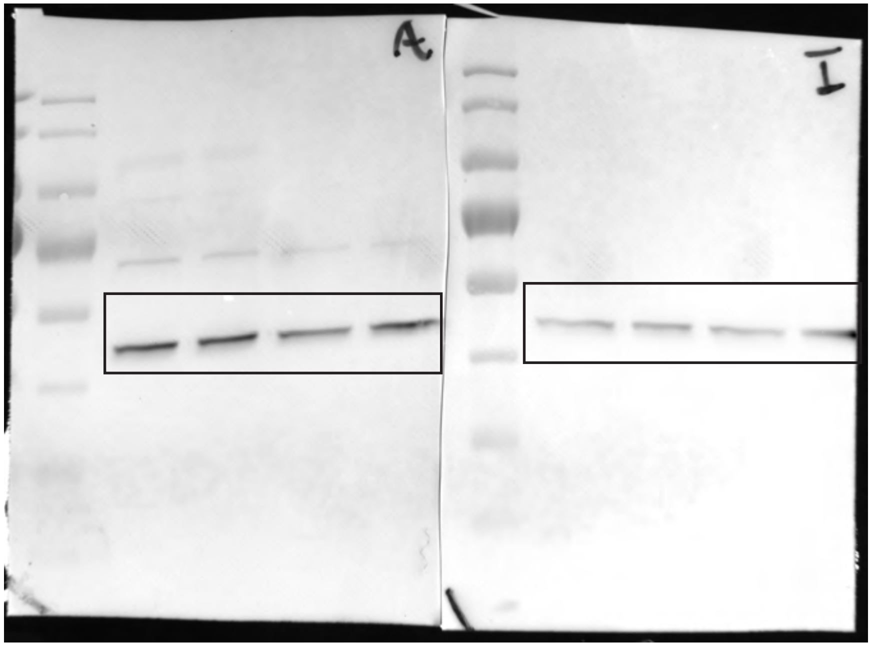

Supplement: Supplemental data [file jciinsight-9-162621-s010.pdf]
